# In-Situ Catalytic Pyrolysis of Polystyrene with Sewage Sludge-Derived Catalysts: Sewage Sludge Ash, Sewage Sludge Char, and Fe-Impregnated Acid-Leached Sewage Sludge Char

**DOI:** 10.3390/polym18192372

**Published:** 2026-09-29

**Authors:** Fedor Pogorov, Diyar Tokmurzin, Yelena Vitoshnova, Nazar Zabara, Anara Omarova, Olga Ibragimova, Aigerim Kaiaidarova, Sergey Nechipurenko, Sergey Efremov, Grigoriy Mun, Mukhambetkali Burkitbayev

**Affiliations:** 1 Center of Physical-Chemical Methods of Research and Analysis, Farabi University, Almaty 050012, Kazakhstan; fpogorov@gmail.com (F.P.); helenvit4@gmail.com (Y.V.); nzr.zabara@gmail.com (N.Z.); omarova@cfhma.kz (A.O.); aigerim_ko@list.ru (A.K.); nechipurenkos@mail.ru (S.N.); efremsa@mail.ru (S.E.); grigoriy.mun@gmail.com (G.M.); mukhambetkali.burkitbayev@kaznu.edu.kz (M.B.); 2 Laboratory for Chemical Technology, Ghent University, Industriele Scheikunde, Technologiepark-Zwijnaarde 125, 9052 Ghent, Belgium

**Keywords:** polystyrene, catalytic pyrolysis, sewage sludge-derived catalysts, sewage sludge ash, sewage sludge char, Fe impregnation, pyrolysis products

## Abstract

Sewage sludge (SS)-derived materials were investigated as low-cost heterogeneous catalytic materials for polystyrene (PS) pyrolysis, focusing on the effects of thermal treatment, HCl leaching, and Fe impregnation. Sewage sludge ash (SSA), sewage sludge char (SSC), and HCl-leached Fe-impregnated sewage sludge char (Fe-LSSC) were evaluated as in-situ catalytic materials. Catalyst-to-PS ratios of 1:1–1:10 were investigated in a horizontal semi-batch tubular reactor operated in an in-situ catalyst–polymer contact mode at 800 °C. Thermal pyrolysis produced 63.9 wt.% PyOil and 12.6 wt.% PyGas. SSA increased the PyOil yield to 78.0 wt.% at 1:10 and promoted lighter aromatics; at 1:1, light aromatics increased to 66.8 wt.%, BTEX from 5.8 to 24.58 wt.%, and PAHs decreased to 11.8 wt.%. SSC showed a weaker, non-monotonic effect, with PyOil decreasing to 54.4 wt.% and PyChar increasing to 4.7 wt.% at 1:2. Fe-LSSC produced the strongest changes, increasing PyGas to 28.0 wt.% at 1:1 and H_2_ to 75.3 vol.% at 1:2. The gasoline-range fraction reached 82.1 wt.% at 1:2, while PAHs decreased to 8.79 wt.% at 1:1. Catalyst reuse altered gas composition, whereas thermal regeneration partially restored catalytic behavior. Overall, SS-derived materials exhibited distinct, loading-dependent catalytic functions, while HCl leaching and Fe impregnation enhanced the conversion of PS pyrolysis vapors toward lighter aromatic and gaseous products.

## 1. Introduction

Plastic waste represents an environmental challenge, causing severe impacts on terrestrial and aquatic ecosystems and consequently posing risks to human health [1]. ARIMA model predictions estimate that global plastic waste generation will increase to 377 million metric tons (Mt) by 2030 and 576 Mt by 2050 [2]. However, mechanical recycling has proven largely insufficient to offset the growth in global plastic production, with only approximately 9% of plastic waste currently being recycled [3]. The current linear model of plastic production and consumption is therefore considered unsustainable, highlighting the need to transition toward circular approaches to plastic waste management [4]. Polystyrene (PS), along with other polymers, is widely used in major industrial sectors, including packaging, construction, and medical equipment manufacturing, making it a significant contributor to plastic waste generation [5].

Conventional polymer waste management relies on mechanical recycling, downcycling, energy recovery through incineration, and landfilling [6,7]. However, mechanical recycling is limited by sorting and contamination issues and progressive deterioration of polymer properties, while incineration may generate toxic gases and particulate emissions [6]. Landfilling causes long-term environmental impacts, and downcycling generally results in declining material quality over successive cycles [7]. These limitations have increased interest in thermochemical valorization, including hydrothermal liquefaction, hydrothermal carbonization, gasification, and pyrolysis, which can convert plastic waste into fuels, chemicals, and valuable gaseous products [8]. Integration with carbon capture, utilization, and storage (CCUS) technologies may further enhance the environmental benefits of these resource-recovery pathways [9].

Among thermochemical approaches, pyrolysis has attracted considerable attention as a promising route for plastic waste valorization. Thermal pyrolysis can produce valuable hydrocarbon products, including gasoline- and diesel-range fractions, pyrolysis oil, waxes, and pyrolysis gas [10]. The distribution of these products during polymer degradation depends primarily on the operating temperature, residence time, reactor configuration, and nature of the feedstock, allowing the product yields to be controlled through appropriate process conditions. However, these parameters alone may be insufficient to achieve high yields of targeted products; therefore, catalysts are employed to enhance process efficiency, control product selectivity, and improve product quality [11]. Depending on the catalyst type, the degradation pathway may be altered through enhanced secondary cracking, deoxygenation, isomerization, cyclization, or aromatization reactions. Commonly employed catalysts for improving the thermal degradation of plastics include zeolites (HUSY, HZSM-5, and Hβ), fluid catalytic cracking (FCC) catalysts, silica–alumina catalysts, and natural clays [12]. However, conventional catalysts can be relatively unavailable, require regeneration or additional preparation, and may be susceptible to deactivation.

The aromatic structure of PS makes its pyrolysis particularly attractive, as the process predominantly yields styrene, BTEX, and other valuable aromatic hydrocarbons, with the product distribution strongly dependent on the reaction conditions and catalyst employed [13]. The use of commercial catalysts has several limitations, including rapid catalyst deactivation associated with high acidity in zeolitic systems and a shift in product selectivity toward gaseous products and light aromatic compounds when FCC catalysts are used [14]. Consequently, increasing attention has been directed toward alternative catalysts that can provide comparable catalytic functionality while offering greater availability, lower cost, and reduced environmental impact. Waste-derived catalysts are of particular interest because their use can potentially reduce catalyst costs while simultaneously contributing to waste valorization. For example, biochar-based catalysts derived from heavy-metal-contaminated manure waste provided a high liquid product yield of 88 wt.%, while increasing the styrene content to 61 wt.% and promoting the formation of monocyclic aromatic hydrocarbons [15]. In another study, activated carbon derived from solid organic waste increased the selectivity toward BTEX compounds [16]. Calcium-based catalysts derived from waste mollusk shells accelerated PS depolymerization at a relatively low temperature of 350 °C and increased the styrene yield to 56.5 wt.% [17].

Among major waste streams, SS requires particular attention because it accumulates pharmaceutical residues, heavy metals, microplastics, and other organic pollutants, whose degradation products may pose significant environmental risks [18]. Global SS production increased from ~45 Mt of dry solids in 2017 [19], to nearly 53 Mt in 2022 [20], and may reach 130 Mt by 2030 [21], along with population growth [22]. Conventional management methods, such as landfilling, agricultural application, composting, and incineration, are associated with emissions, high energy demand, long processing times, and land constraints [23,24]. Consequently, thermochemical valorization is increasingly attractive because it can simultaneously reduce waste volume, recover energy, and generate value-added products [25]. Sewage sludge ash (SSA) from incineration and sewage sludge char (SSC) from thermochemical treatment are potential functional catalytic materials. SSA is a mineral-rich material dominated by SiO_2_, CaO, Al_2_O_3_, Fe_2_O_3_, MgO, and P_2_O_5_, with quartz, whitlockite-like calcium phosphates, and anhydrite as major crystalline phases [26,27], whereas SSC is a carbonaceous–mineral composite whose composition depends strongly on pyrolysis temperature [24]. Their mineral composition and structure govern their catalytic behavior: alkaline and alkaline-earth species provide basic sites that promote cracking, deoxygenation, and reforming [28], while the carbon matrix of SSC can enhance reactant accessibility and active-species dispersion through its porous structure and surface functional groups [29]. Acid treatment further modifies both materials by leaching P, Ca, and Mg, producing leached SSC (LSSC) and SSA (LSSA) with substantially altered catalytic properties [30]. To enhance the activity and selectivity of these element-depleted materials, impregnation with Fe(III) salts can be employed as an effective modification strategy owing to their high reactivity, redox properties, availability, and environmental compatibility [31]. However, studies investigating the catalytic pyrolysis of PS using SSA, SSC, LSSC, LSSA, and acid-leached Fe-impregnated SS-derived materials remain very limited.

Despite growing interest in SS-derived materials as low-cost catalysts, their application in plastic pyrolysis, particularly for PS, remains limited and underdeveloped. SSA and SSC can promote cracking, dehydrogenation, aromatization, and other secondary reactions, but their performance strongly depends on chemical composition, surface properties, and preparation history. The effects of acid leaching and subsequent metal incorporation have received comparatively little attention. Acid leaching can remove phosphorus- and calcium-containing phases and alter surface properties, whereas Fe incorporation may introduce additional active species and modify acidity and reaction pathways [31]. However, the relationship between these modifications and PS pyrolysis product distribution remains insufficiently established, and direct comparisons of untreated and modified SS-derived catalysts under identical conditions are lacking. Therefore, a key knowledge gap concerns the systematic evaluation of ash-, char-, acid-leached-, and Fe-impregnated SS-derived materials for PS pyrolysis. In particular, it remains unclear how these modifications affect catalytic activity and the distribution of gaseous and liquid products. Establishing these relationships is essential to determine whether SS-derived materials can serve not only as low-cost alternatives to conventional catalysts but also as tunable catalysts whose properties and product selectivity can be tailored through chemical modification.

Therefore, this study investigates the catalytic pyrolysis of PS using SS-derived materials, including SSA, SSC, and Fe-LSSC. Particular emphasis is placed on elucidating how acid leaching and Fe incorporation modify the catalytic performance and influence the distribution and composition of pyrolysis products, providing a basis for the valorization of SS-derived materials as tunable catalysts for plastic waste conversion.

## 2. Materials and Methods

### 2.1. Sample Preparation

The SS used in this study was collected from the aeration station of the State Municipal Enterprise “Almaty Su” (Almaty, Kazakhstan). Figure S1 provides a schematic summary of the catalyst preparation process. The original material was first dried in a drying oven at 105 °C for 24 h. In a porcelain mortar, the sludge was ground and homogenized. The dried SS was sieved to a particle size of <0.315 mm, with losses occurring in the form of organic and plant residues.

The original SS was subjected to incineration at 900 °C in a muffle furnace in an air atmosphere for 1 h for dry ashing in order to obtain SSA samples. Another sample was carbonized in an inert (Ar) atmosphere with a gas flow of 50 mL·min^−1^ under the same temperature regime and it was designated as SSC.

SSC was leached using 3 M HCl: SSC was mixed with acid in a 1:20 ratio and agitated for 5 h at 80 °C. Following the acid treatment, the solution was cooled to ambient temperature before being filtered using a Büchner funnel and subsequently washed with distilled water to remove residual acid. After washing, LSSC was dried for 3 h at 105 °C in a laboratory drying oven. Iron(III) nitrate nonahydrate was used to impregnate the acid-leached char: after dissolving 7.21 g of Fe(NO_3_)_3_ · 9H_2_O in 50 mL of isopropyl alcohol per 10 g of LSSC and mixing for 30 min, 10 g of LSSC was added, and the mixture was further mixed for 5 h at 80 °C until a slurry formed [32,33]. After that, the slurry was oven-dried at 105 °C for 12 h. The dried sample was then calcined at 900 °C for 1 h in a muffle furnace in an inert atmosphere. Catalyst samples were dried and then pulverized into a powder. The impregnated catalytic material sample was designated as Fe-LSSC. To evaluate catalyst reusability, solid residues after pyrolysis experiments containing pyrolysis char (PyChar) and Fe-LSSC were collected and designated as Fe-LSSC^i^, where “i” stands for the number of applications of this catalyst. After each pyrolysis cycle, the solid residue was recovered, weighed, and reused as the catalyst in the subsequent cycle. Since the recovered solid mass was slightly higher than the initial catalyst loading, the amount of recovered catalyst required to maintain the nominal Fe-LSSC/PS ratio of 1:4 was used for each subsequent experiment. One sample of the PyChar residue Fe-LSSC was subjected to incineration in an air atmosphere at 900 °C for 20 min until all deposited carbon volatilized. This sample was designated as Fe-LSSC-red.

### 2.2. Characterization

The PS feedstock used in this study was obtained from waste packaging material. Prior to pyrolysis, the packaging was washed to remove surface contaminants, the labels were manually removed, and the material was dried. Then, PS was ground using a rotor-blade mill equipped with a 1 mm sieve. Elemental composition of the PS and raw SS was determined using a CHNS/O analyzer (Vario MicroCube, Elementar Analysensysteme GmbH, Hanau, Germany). Volatile matter, ash content, and fixed carbon were determined gravimetrically according to the standard proximate analysis procedure. The catalytic materials were characterized by X-ray diffraction (XRD), X-ray fluorescence (XRF), scanning electron microscopy (SEM), and Brunauer–Emmett–Teller (BET) surface area analysis. XRF was employed to determine the elemental composition of the catalytic samples, using an Axios 1 kW wavelength-dispersive X-ray fluorescence spectrometer (PANalytical, Malvern, UK). Data processing and interpretation were performed with SuperQ version 5.0 software (PANalytical, Malvern, UK). The relative error of the semi-quantitative XRF analysis was within ±20%. XRD analysis was conducted to identify the mineral phases present in SS and to evaluate their transformations following incineration, calcination, and impregnation. Measurements were carried out using a Bruker D8 Advance diffractometer (Bruker AXS SE, Karlsruhe, Germany). The porous structure of the samples was investigated using nitrogen adsorption at 77 K on a Quantachrome Autosorb iQ (Quantachrome Instruments, Boynton Beach, FL, USA) gas sorption analyzer. Prior to analysis, the samples were degassed under dynamic vacuum at 150 °C for 6 h. Nitrogen adsorption–desorption isotherms were recorded over a relative pressure range of 10^−6^ to 0.995. The specific surface area was calculated using both BET and density functional theory (DFT) models. Surface morphology and microstructure were examined by scanning electron microscopy using a SEM5000Pro instrument (CIQTEK, Hefei, China), equipped with an Oxford Instruments X-Plore 30 energy-dispersive X-ray spectroscopy (EDS) system with a silicon drift detector (SDD). The surface acidity and basicity of the catalysts were evaluated using the methods of temperature-programmed desorption of ammonia (NH_3_-TPD) and temperature-programmed desorption of carbon dioxide (CO_2_-TPD) on a USGA-1 analyzer (“UNISIT”, Moscow, Russia).

### 2.3. Thermogravimetric Analysis (TGA)

TGA (Netzsch STA 449, Selb, Germany) was used to determine the characteristics of the thermal degradation of plastic samples mixed with catalysts at catalyst–plastic ratios of 1:1, 1:2, 1:4. The mass of the samples ranged from 40 to 70 mg. The samples were heated in a nitrogen atmosphere up to 850 °C at a rate of 10 °C·min^−1^, with a gas flow rate of 50 mL·min^−1^. This temperature is significantly higher than the temperature of complete decomposition of the PS sample. To eliminate the contribution of the inorganic catalyst mass, the TGA and DTG profiles of the catalyst-containing samples were normalized to the initial PS mass on a catalyst-free basis.

### 2.4. Horizontal Semi-Batch Tubular Reactor Setup

The pyrolysis experiments were conducted in a horizontal semi-batch tubular reactor operated in an in-situ catalyst–polymer contact mode under slow pyrolysis conditions. The schematic of the experimental setup is shown in Figure 1. The PS and catalyst were premixed and placed as a static batch (6) in an open ceramic boat (10 cm in length and 1 cm in width) (5), which was positioned inside an externally heated quartz tube (4) located within a horizontal tube furnace (3). The quartz tube had an inner diameter of 5 cm and a total length of 95 cm, with approximately 35 cm located within the heated zone of the furnace, corresponding to a geometric empty-section volume of approximately 690 mL. Argon was continuously supplied from a gas cylinder (1), passed through a flow meter (2), and entered the quartz tube as the carrier gas. During pyrolysis, the generated volatile products were continuously transported by the Ar flow from the heated reaction zone to the downstream condensation and gas-collection system. The exposed sections of the quartz tube were thermally insulated with glass wool. The reactor outlet was connected through an insulated line to a cold-trap system (7) for the collection of condensable products. The cold-trap system consisted of three impinger bottles placed in a cold-water bath, each containing 50 mL of isopropyl alcohol. After passing through the cold traps, the non-condensable gaseous products were collected in a Tedlar bag. The collected PyGas and liquid fractions were subsequently analyzed as described below.

### 2.5. Experimental Procedure

The catalyst-to-PS ratios of 1:1–1:10 were selected to systematically evaluate the effect of catalyst loading on the pyrolysis behavior and product distribution. The highest catalyst loading was included as an experimental condition to characterize the catalytic response at a high catalyst concentration and does not represent a proposed industrial operating ratio. Catalysts based on SS (SSA, SSC, Fe-LSSC) were mixed with PS at mass ratios of 1:1 (SSA or SSC or Fe-LSSC/PS 1:1), 1:2 (SSA or SSC or Fe-LSSC/PS 1:2), 1:4 (SSA or SSC or Fe-LSSC or Fe-LSSC^i^ or Fe-LSSC^ii^ or Fe-LSSC^iii^ or Fe-LSSC-red/PS 1:4), and 1:10 (SSA or SSC or Fe-LSSC/PS 1:10), obtaining samples with a total mass of 10 g. The prepared mixtures were placed into ceramic boats and then loaded into a quartz tubular reactor. At the beginning of the experiment, argon was supplied and maintained throughout the entire pyrolysis process. The carrier gas (Ar) flow was controlled using a needle valve and introduced into the reactor at a rate of 120 mL·min^−1^. Figure S2 shows the four-stage heating program used for pyrolysis. In stage (a), the temperature was increased from 25 to 300 °C over 28 min (9.8 °C·min^−1^), followed by a 5 min isothermal hold at 300 °C (stage b). During this stage, a Tedlar gas bag was connected for collection of volatile products and remained connected until the end of the experiment. In stage (c), the temperature was raised from 300 to 800 °C over 50 min (10 °C·min^−1^), followed by a 15 min isothermal hold at 800 °C (stage d). After completion of the heating program, the furnace was switched off and allowed to cool to ambient temperature. The Tedlar bag and impinger bottles were then disconnected. Liquid products collected in the three impingers were combined into a single flask, while the solid residue, consisting mainly of coked catalyst, was recovered after the reactor had cooled. Residual pyrolysis Heavy PyOil was dissolved in isopropanol and separated by filtration. The Heavy PyOil formation was attributed to condensation in colder regions near the reactor outlet. All experiments were performed in triplicate, and the results are reported as mean ± standard deviation (SD).

In this study, the terms catalyst and catalytic material refer to the heterogeneous SS-derived solids used to promote secondary transformation of PS-derived pyrolysis vapors. The reported catalyst-to-PS ratios represent bulk mass ratios of the entire solid material to PS and do not represent stoichiometric ratios between catalytically active sites and polymer molecules. SSA, SSC, and Fe-LSSC are chemically heterogeneous materials containing mineral, acid–base, and carbonaceous components and, for Fe-LSSC, Fe-containing functionalities, of which only a fraction constitutes accessible catalytic sites. For example, NH_3_-TPD determined total acid-site concentrations of 175, 274, and 167 μmol g^−1^ for SSA, SSC, and Fe-LSSC, respectively. Consequently, the relatively high bulk-solid loadings employed in this study were selected to provide sufficient contact between evolving PS vapors and the accessible surface of these low-cost waste-derived materials rather than to provide stoichiometric quantities of active species.

### 2.6. Product Collection and Analysis

Figure 2 schematically illustrates the distribution of products obtained during catalytic pyrolysis of PS into three main fractions: non-condensable gases, condensable liquids, and solid residue. The condensable products comprised an oil fraction (PyOil), consisting mainly of aromatic and low-molecular-weight hydrocarbons, and a Heavy PyOil fraction containing predominantly high-molecular-weight oligomers that condensed on a quartz tube. After each experiment, the solid residue was collected in pre-weighed zip-lock bags and weighed using an analytical balance (±0.0001 g). Condensable products were recovered by passing the volatile stream through isopropanol. Heavy PyOil deposited on the inner wall of the quartz reactor was removed, weighed separately, redissolved in the PyOil with stirring for ~30 min, and the resulting filtrate was subjected to GC-MS analysis. The remaining insoluble residue was weighed and included in the overall mass balance. Approximately 5 mL of the mixed oil fraction (PyOil + Heavy PyOil) was collected for GC–MS analysis, while the remainder was evaporated in a STEGLER RI-213 rotary evaporator at 60 °C under reduced pressure to constant mass. The mass of Heavy PyOil was then subtracted from the resulting total mass to determine the mass of PyOil, which was included in the material balance. PyGas samples collected in Tedlar bags were analyzed by gas chromatography. Their total volume was determined using a series-connected pump (220-1000TC PocketPump, SKC, Eighty Four, PA, USA) with controlled flow and a Restek ProFLOW 6000 flowmeter (Restek Corporation, Bellefonte, PA, USA) for flow monitoring and calibration.

Because solvent removal under reduced pressure can simultaneously remove low-boiling pyrolysis compounds, the gravimetrically determined oil yield represents the recovered non-volatile condensable fraction rather than the absolute total condensable yield. An aliquot of the IPA-trapped liquid was therefore withdrawn before evaporation for GC–MS characterization, avoiding solvent-removal losses during compositional analysis. In addition, a minor fraction of the permanent and light gases may have dissolved in the IPA during collection and subsequently been released during reduced-pressure evaporation. This dissolved-gas fraction was not independently quantified and was therefore not included in the measured PyGas yield. Consequently, the difference between the summed product yields and 100 wt.% is reported as the overall mass-balance deficit, incorporating losses of volatile condensables, possible dissolved-gas losses, and other collection/handling uncertainties.

### 2.7. GC-MS Analysis of the Isopropanol-Trapped Fraction

Before analysis, the liquid fraction collected in the cold traps was filtered through a 0.22 μm membrane and analyzed by gas chromatography–mass spectrometry (GC–MS; Agilent 7890A/5975C, Wilmington, DE, USA). Samples trapped in isopropanol (0.5 μL) were injected at 240 °C in split mode (5:1). Separation was performed on a DB-624 Ultra Inert capillary column (60 m × 0.25 mm, 1.4 μm; Agilent, USA) using helium (1.0 mL·min^−1^) as the carrier gas. The oven temperature was held at 40 °C for 5 min, increased to 240 °C at 10 °C·min^−1^, and maintained for 15 min (total analysis time: 40 min). Mass spectra were acquired in scan mode (*m*/*z* 10–550). The ion source, quadrupole, and interface temperatures were set at 230, 150, and 250 °C, respectively. To eliminate chromatographic interference from isopropanol, MS detection was disabled between 7 and 10 min. Gaseous products (500 μL) collected in 15-L Tedlar bags were analyzed by GC with a thermal conductivity detector (GC-TCD; Agilent, Wilmington, DE, USA) using two analytical methods. Separation was performed on a Carboxen^®^ 1010 PLOT capillary column (30 m × 0.53 mm, 30 μm; Supelco, Bellefonte, PA, USA) for the determination of H_2_, CO, CH_4_, CO_2_, C_2_H_2_, C_2_H_4_, C_2_H_6_, C_3_H_6_, and C_3_H_8_. The injector temperature was maintained at 200 °C with split ratios of 2:1 for hydrogen and 50:1 for hydrocarbons. The TCD operated at 230 °C with reference and auxiliary flows of 18 and 7 mL·min^−1^, respectively. Helium served as the carrier gas for hydrocarbon analysis and nitrogen for hydrogen analysis. For hydrocarbon analysis, the oven temperature was held at 35 °C for 5 min, increased to 240 °C at 24 °C·min^−1^, and held for 23 min (total analysis time: 36.5 min). For hydrogen analysis, the oven was held at 35 °C for 7 min, heated to 240 °C at 60 °C·min^−1^ (total analysis time: 10.4 min), and the TCD operated in negative polarity mode from 1 to 6.5 min. Calibration was performed using certified standard gas mixtures supplied by LLC Monitoring (Saint-Petersburg, Russia): 10 vol.% H_2_ in N_2_ and a hydrocarbon mixture containing 10 vol.% of each component with 20% N_2_. The certified uncertainty was 2.5–3% per component. Standard gases were introduced through a four-port valve equipped with a 500 μL sample loop at different split ratios.

### 2.8. Statistical Analysis

All pyrolysis experiments were independently performed in triplicate (n=3), and results are reported as mean ± standard deviation (SD). Statistical differences among experimental conditions were evaluated separately for each response variable using one-way analysis of variance (ANOVA), followed by Tukey’s honestly significant difference (HSD) post-hoc test for multiple pairwise comparisons. Differences were considered statistically significant at p<0.05. For PyGas data, each gaseous component was analyzed independently across the corresponding catalyst-loading or catalyst-reuse conditions; different gaseous species were not pooled into a common statistical comparison.

## 3. Results and Discussion

### 3.1. PS and Raw Sewage Sludge Characterization

The results of the proximate and CHNS analyses of the PS and raw sewage sludge used in this study are presented in Table 1. According to the obtained data, PS is characterized by a high content of carbon and hydrogen with the near-complete absence of other elements, which confirms its chemical structure. In addition, the analysis results showed a negligible nitrogen content (0.03%), the presence of which is likely associated with the use of additives introduced during the polymer production process [34]. The raw SS contained 34.2 wt.% volatile matter and 60.7 wt.% ash, indicating a predominantly inorganic composition with a comparatively smaller combustible fraction. The elemental analysis further showed relatively low contents of CHNS, consistent with the mineral-rich nature of the precursor used for catalyst preparation.

### 3.2. XRF, XRD, and BET Analysis Results

According to the XRF results (Table S1), SSA, SSC, and Fe-LSSC consist predominantly of oxygen- and silicon-containing components. Oxygen contents were 47.6, 46.0, and 49.4 wt.%, while silicon accounted for 17.8, 18.0, and 24.5 wt.% in SSA, SSC, and Fe-LSSC, respectively. Their high abundance reflects the predominance of inorganic mineral phases in SS, including quartz (SiO_2_) and aluminosilicates such as albite, microcline, and gehlenite. Acid leaching of SSC substantially reduced the contents of Ca, P, S, and Mg. Sulfur removal is important to prevent poisoning of metallic active sites through the formation of thermally stable sulfides [35,36,37], whereas Ca neutralizes Brønsted acid sites, reducing cracking activity and promoting irreversible degradation of the zeolite framework and pore blockage [38,39]. Acid leaching followed by Fe impregnation increased the Fe content from 3.7 to 8.9 wt.%.

XRD analysis (Table 2, Figure S3) confirmed that, besides quartz, SSA and SSC contained abundant calcium-bearing and aluminosilicate phases, together with crystalline Fe_2_O_3_ (5.7 and 6.2 wt.%, respectively). Heating dried SS to 900 °C can successfully convert CaO into Ca_2_Al_2_SiO_7_, CaSO_4_, Ca_4_P_2_O_9_ phase structures, but the conversion efficiency varies significantly for each compound based on thermodynamic limits. At 900 °C, the calcium oxide (CaO) matrix reacts aggressively with the volatilizing sulfur, phosphorus, and aluminosilicates inherent to the sludge [40]. CaO acts as an effective in situ desulfurization agent. As the organic sulfur in the SS combusts and volatilizes into SO_2_ or SO_3_, it reacts directly with the free lime [41]. At 900 °C, CaSO_4_ can remain detectable in thermally treated sewage-sludge-derived materials, although its stability depends on the initial composition and treatment conditions [42]. In CaO–Al_2_O_3_–SiO_2_-containing systems, gehlenite (Ca_2_Al_2_SiO_7_) has been reported to form at high temperatures, including within the 850–1050 °C range [43].

In contrast, Fe-LSSC showed no crystalline gehlenite, calcium sulfate, or calcium phosphate, confirming their removal during acid treatment. Hydrochloric acid decomposes Ca_2_Al_2_SiO_7_ by selectively removing Ca and part of Al, producing an amorphous silica-rich residue, consistent with the XRF and XRD results [44]. Despite the higher Fe content, no crystalline Fe_2_O_3_ was detected in Fe-LSSC, indicating that iron oxide was dispersed within the amorphous matrix and therefore remained undetectable by conventional XRD [45].

XRD analysis of char residues after catalytic pyrolysis of PS with SS-derived catalysts (Tables S2–S4) revealed significant changes in phase composition compared with the fresh catalysts, indicating solid-state transformations between catalyst minerals and polymer decomposition products. The albite content remained nearly unchanged, whereas gehlenite and calcium sulfate in spent SSA and SSC catalysts markedly decreased or disappeared. Gehlenite is known to transform into calcium silicate and calcium aluminate phases during heating through Ca, Al, and Si redistribution [46].

A major change was the disappearance of maghemite (Fe_2_O_3_) and the formation of magnetite (Fe_3_O_4_). Magnetite contents reached 2.7, 1.0, and 1.3 wt.% for SSA/PS ratios of 1:1, 1:2, and 1:4, respectively, and 8.1, 6.6, and 1.3 wt.% for Fe-LSSC/PS. This transformation results from the reduction of Fe_2_O_3_ by carbon formed during polymer degradation and subsequent reaction with elemental iron under oxygen-free conditions (2Fe_2_O_3_ + C = Fe_3_O_4_ + CO_2_ + Fe, 4Fe_2_O_3_ + Fe = 3Fe_3_O_4_) [47,48,49]. In contrast, neither maghemite nor magnetite was detected in spent SSC, likely because iron oxides were present in the amorphous phase.

The surface characteristics of SS-derived catalysts (Table 3) showed low specific surface area and pore volume, indicating limited porosity prior to modification. These values agree with previous research for non-activated sewage-sludge-derived materials, where the BET surface area was 2.043 m^2^ g^−1^ in raw SS [50]. Acid leaching and Fe impregnation increased the surface area to 8.04 m^2^ g^−1^, pore volume to 0.034 cm^3^ g^−1^, and average pore size to 1.38 nm, indicating improved pore development and accessibility of active sites. However, the resulting catalytic material exhibited substantially lower BET surface area than that reported for SSC derived from SS collected at the Jiaxing Sewage Treatment Plant (Jiaxing, Zhejiang, China), which exhibited a specific surface area of approximately 140 m^2^ g^−1^ [51]. This difference may be attributed to variations in the origin and composition of the SS feedstock [52].

### 3.3. TPD Analysis

The TPD analysis results are presented in Figure 3. The CO_2_-TPD profiles of the investigated samples are presented in Figure 3a. All samples exhibit weak CO_2_ desorption below ~400 °C, corresponding to weak and partially medium-strength basic sites of the Ca–Si–Al matrix, whereas the dominant desorption at 500–700 °C is associated with strong basic sites and stable carbonate species. Fe-LSSC shows moderate low- and medium-temperature desorption but a pronounced high-temperature region extending to 700–750 °C, indicating the predominance of strong basic sites associated mainly with Fe oxide phases. SSC exhibits an intense maximum at ~644 °C, consistent with its high Ca content (13.17 wt.%) and the presence of Si, Al, P, and S, which promote stable Ca-containing carbonate and mixed oxide phases. In contrast, SSA displays a lower-temperature maximum (~590 °C), indicating less stable carbonate species. The position of the high-temperature CO_2_ desorption maximum differed considerably among the investigated materials, occurring at ~590 °C for SSA, ~644 °C for SSC, and ~737 °C for Fe-LSSC. The Fe contents of SSA (3.41 wt.%) and SSC (3.72 wt.%) are comparable within the uncertainty of the semi-quantitative XRF analysis. Instead, the distinct CO_2_-TPD behavior of SSA and SSC is likely associated with differences in their mineral and surface composition, including the distribution of Ca-containing and aluminosilicate phases and the presence of a carbonaceous matrix in SSC. The higher-temperature desorption observed for Fe-LSSC may reflect the combined effects of Fe incorporation and the substantial modification of the mineral and surface environment caused by acid leaching and subsequent impregnation. Overall, Fe-LSSC possesses the strongest basic sites, SSC is distinguished by intense Ca-related basicity, and SSA exhibits comparatively weak CO_2_ fixation.

The NH_3_-TPD profiles demonstrate the presence of weak, medium, and strong acid sites in all investigated SS-derived materials, confirming the heterogeneous nature of their surface acidity (Figure 3b). The total concentration of acid sites was 175 μmol·g^−1^ for SSA, 274 μmol·g^−1^ for SSC, and 167 μmol·g^−1^ for Fe-LSSC (Table 4), indicating that SSC exhibited the highest overall acidity among the investigated samples. The distribution of acid sites also differed substantially between the materials. For SSA and SSC, the profiles indicated a predominant contribution of strong acid sites. In contrast, Fe-LSSC exhibited a different acidity distribution, with acid leaching and Fe modification shifting the contribution toward medium-strength sites, accompanied by a lower contribution of strong acid sites compared with the pristine SSC.

The high-temperature NH_3_ desorption regions further indicate the presence of strongly bound acid sites in the SS-derived materials. The predominance of strong acidity in SSC may be associated with its carbon–mineral matrix and the presence of acid-forming inorganic components, whereas the redistribution of acid sites in Fe-LSSC may be related to modification of the surface chemical environment by iron-containing species. A similar redistribution of acidity upon Fe modification was previously reported by Chen et al. [53]. From a catalytic perspective, the coexistence of acid sites with different strengths may be relevant to the transformation of polymer-derived vapors: weak and medium-strength sites can contribute to the adsorption and initial conversion of reactive intermediates, whereas strong acid sites promote secondary hydrocarbon transformations [54]. Thus, the higher total acidity and greater contribution of strong acid sites in SSA and SSC indicate a higher density of acidic functionalities available for catalytic interactions, whereas the greater contribution of medium-strength sites in Fe-LSSC reflects a modification of the surface acidity distribution rather than a simple increase in the overall number of acid sites.

### 3.4. SEM-EDX Analysis Results

Figure 4 demonstrates SEM analysis for SSA, SSC and Fe-LSSC. For SSA (Figure 4a), the SEM shows aggregates of irregularly angular particles with a wide range of sizes; the particles have a dense structure and relatively clear boundaries. For SSC (Figure 4b), compared with the SSA sample, the particles show a rougher surface texture and a more fragmented morphology, reflecting the release of volatile compounds during pyrolysis and the formation of a carbonaceous matrix. Fe-LSSC retains the irregular morphology of the original SSC while exhibiting a widespread distribution of iron across the particle surfaces (Figure 4c). Elemental mapping indicates that Fe is dispersed throughout the sample without the formation of large isolated agglomerates, suggesting successful impregnation of the carbonized sludge. Calcium-, silicon-, and oxygen-rich domains remain visible, while iron-containing regions are superimposed on the existing mineral matrix. The relatively homogeneous Fe distribution implies effective interaction between the iron species and the char surface. More detailed images of the catalysts are shown in Figure S4.

### 3.5. TGA Results

Figure 5 presents the results of normalized thermogravimetric analysis (TGA) and normalized differential thermogravimetry (DTG) of mixtures of the investigated catalysts obtained from SS with PS in comparison with the original polymer. Mixtures of PS with SSA were prepared at mass ratios of 1:1 (SSA/PS 1:1), 1:2 (SSA/PS 1:2), and 1:4 (SSA/PS 1:4). Similarly, mixtures of PS with SSC (SSC/PS 1:1; SSC/PS 1:2; SSC/PS 1:4) and Fe-LSSC (Fe-LSSC/PS 1:1; Fe-LSSC/PS 1:2; Fe-LSSC/PS 1:4) were prepared.

The TGA and DTG profiles of the catalyst-containing samples were normalized to the initial PS mass after correction for the catalyst mass contribution, allowing direct comparison of PS degradation behavior at different catalyst loadings. In all three catalyst series, a slight increase in the degradation onset temperature (T_onset) was observed compared with pristine PS. For SSA and SSC, the shift was approximately 4–9 °C, whereas Fe-LSSC/PS 1:1 exhibited a more pronounced shift of approximately 20 °C. Rather than indicating a simple increase in thermal stability, these shifts suggest that interactions between PS and the catalyst modify the onset and progression of polymer degradation, potentially through differences in mass transfer, polymer–surface contact, and accessibility of catalytic sites [55,56,57]. The normalized DTG profiles showed moderate changes in the position and shape of the main decomposition peak, with the most pronounced shift observed for Fe-LSSC/PS 1:1. This behavior indicates modification of the PS degradation pathway in the presence of Fe-containing and mineral phases [32]. This effect is consistent with the influence of the mineral matrix, which suppresses the rapid primary depolymerization stage while simultaneously promoting secondary cracking and altering the pathways of polymer thermal decomposition on the catalyst surface [58].

### 3.6. Product Distribution and Yield

Figure 6 shows the product yields from catalytic PS pyrolysis calculated relative to the initial plastic mass. The overall mass-balance closure ranged from approximately 82 to 99 wt.%. The unaccounted fraction is attributed primarily to loss of low-boiling condensable compounds during removal of IPA under reduced pressure, although release of gases dissolved in the trapping solvent and other collection or handling losses may also contribute. Accordingly, the gravimetrically determined PyOil yields should be considered recovered condensable yields rather than absolute total condensable yields. Non-catalytic PS pyrolysis produced 63.9 wt.% PyOil, 12.6 wt.% PyGas, and 0.9 wt.% solid residue.

For SSA (Figure 6a), decreasing the catalyst loading increased the PyOil yield from 57.7 wt.% (1:1) to 71.8 wt.% (1:2), 70.7 wt.% (1:4), and 78.0 wt.% (1:10), while PyGas and PyChar yields decreased. This indicates enhanced depolymerization and suppression of secondary cracking, likely due to Ca–Si–Al phases and the surface acid–base properties. Mineral components and surface functional groups of SS-derived chars are known to promote dehydrogenation, hydrogen transfer, ring opening, and aromatization, thereby affecting liquid product formation [51]. A similar trend was observed for SSC (Figure 6b), although the effect depended on catalyst loading. At an SSC/PS ratio of 1:1, the PyOil yield slightly exceeded that of thermal pyrolysis, whereas at 1:2 it decreased to 54.4 wt.% with an increase in PyChar yield to 4.7 wt.%. Enhanced PyChar formation may result from local overheating within catalyst pores and intensified secondary and tertiary reactions [59,60]. Catalyst agglomeration at high loadings may further promote coking, consistent with the behavior observed for all catalysts at 1:1 and 1:2 ratios [61].

The strongest catalytic effect was obtained with Fe-LSSC (Figure 6c). At a Fe-LSSC/PS ratio of 1:1, the PyGas yield increased from 12.6 to 28.0 wt.%, reflecting enhanced C–C and C–H bond cleavage by Fe-containing active sites and subsequent formation of low-molecular-weight gaseous products [62]. In addition, Fe_2_O_3_ may act as a redox-active phase, promoting gas formation while stabilizing aromatic compounds through oxygen and hydrogen transfer [63]. In contrast, the Fe-LSSC/PS ratio of 1:10 produced a material balance similar to thermal pyrolysis. Catalyst reuse substantially altered the overall product distribution (Figure 6d). At the nominal Fe-LSSC/PS ratio of 1:4, the PyOil yield was 59.06 wt.% after the first reuse and subsequently increased to 69.34 and 82.73 wt.% after the second and third reuse cycles, respectively, while the solid residue decreased to 0.19 wt.% by the third cycle. These changes indicate progressive modification/deactivation of the catalyst during repeated use, which reduced secondary cracking and favored recovery of condensable products. Following thermal regeneration (Fe-LSSC-red), the product distribution shifted again, confirming that removal of deposited carbon partially restored catalyst functionality.

### 3.7. Pyrolysis Gases Yield and Composition

The yield (wt.% relative to the plastic feed) and composition (vol.%) of the PyGas are presented in Figure 7 and Figure 8. The gaseous products obtained from the catalytic pyrolysis of PS consisted predominantly of H_2_ and CH_4_ (Figure 8), as well as CO, CO_2_, and light hydrocarbons. In comparison with non-catalytic pyrolysis, which was characterized by predominant formation of CH_4_ (4.6 wt.%), CO (2.7 wt.%), and C_2_H_4_ (2.5 wt.%), the introduction of SS-derived catalysts resulted in a redistribution of the gas composition. For SSA (Figure 7a and Figure 8a), the most pronounced effect was observed at an SSA/PS ratio of 1:1, where the yields of CO_2_ and CO increased significantly to 7.8 and 5.3 wt.%, respectively, accompanied by a high PyGas yield. As the catalyst loading decreased, the CH_4_ content gradually increased, whereas the overall PyGas yield and the yields of its individual components decreased markedly, with the gas composition approaching that of thermal pyrolysis. SSC (Figure 7b and Figure 8b) exhibited a more pronounced increase in gas production at a ratio of 1:1; however, the concentrations of C_2_H_4_ and CH_4_ progressively increased to 15.4 and 31.7 vol.%, respectively, at a ratio of 1:4, indicating greater retention of hydrocarbon fragments under less intensive secondary conversion.

The most pronounced changes were observed for Fe-LSSC (Figure 7c and Figure 8c). With decreasing catalyst loading, the concentrations of CH_4_ and C_2_H_4_ decreased markedly. At a Fe-LSSC/PS ratio of 1:2, the H_2_ concentration increased to 75.3 vol.%, whereas the relative concentrations of the other PyGas components decreased. It should be emphasized that this value represents the composition of the non-condensable gas rather than the overall H_2_ yield relative to the PS feed. The pronounced enrichment of the gas phase in H_2_ suggests enhanced secondary dehydrogenation and cracking/reforming reactions of PS-derived vapors over Fe-containing sites, which can facilitate C–H bond activation and molecular hydrogen formation, in agreement with previous studies [64,65]. At the lowest catalyst loading (1:10), C_2_H_4_ was not detected, while C_2_H_2_ and C_3_H_6_ appeared, and the C_2_H_6_ concentration increased substantially to 6.3 vol.%. This redistribution may reflect the lower availability of Fe active sites, resulting in simultaneous dehydrogenation and hydrogenation of C_2_-C_3_ intermediates and the formation of C_2_H_2_, C_2_H_6_, and C_3_H_6_. Similar effects of active-site concentration on the distribution of light hydrocarbons have been reported for metal-containing and acidic catalysts [66].

Repeated use of Fe-LSSC (Figure 7d and Figure 8d) resulted in a gradual increase in CH_4_ concentration, reaching 42.3 vol.% after the third cycle, accompanied by a decrease in H_2_ content, suggesting catalyst deactivation associated with carbon deposition. Following regeneration (Fe-LSSC-red), the H_2_ concentration increased to 68.3 vol.%, while the CH_4_ concentration decreased to 13.3 vol.%, indicating partial restoration of the active surface after carbon removal.

One-way ANOVA followed by Tukey’s HSD test confirmed statistically significant treatment effects (p<0.05) for the PyGas yield and composition data shown in Figure 7 and Figure 8. For the Fe-LSSC series, the total PyGas yield obtained at a catalyst-to-PS ratio of 1:1 was significantly higher than those obtained at the other Fe-LSSC loadings (p<0.05). In contrast, the H_2_ concentration reached 75.3±3.4 vol.% at Fe-LSSC/PS 1:2 and was significantly higher than the H_2_ concentrations measured for PS and the other Fe-LSSC loadings (p<0.05). During catalyst reuse, progressive changes in the gas composition were also statistically supported. In particular, regeneration increased the H_2_ concentration to 68.3±3.8 vol.%, which was significantly higher than the values obtained after the successive reuse cycles (p<0.05). The complete ANOVA and Tukey’s HSD comparisons for the individual PyGas components are provided in Table S22.

### 3.8. Pyrolysis Oil Characteristics

Figure 9 presents the relative composition of PyOil obtained during catalytic PS pyrolysis using SS-derived catalysts compared with thermal pyrolysis. The liquid fraction consisted predominantly of aromatic compounds (~95%). Detailed GC–MS results are summarized in Tables S5–S21. For comparison, hydrocarbons were classified into light aromatics (benzene, toluene, ethylbenzene, styrene, α-methylstyrene, xylene, and vinylbenzene derivatives), oligomers, PAHs, cyclic unsaturated compounds, and oxygen-containing compounds. Oligomers were considered indicators of secondary repolymerization and coupling reactions [67], whereas PAHs reflected secondary aromatization, char formation, and cracking intensity [68]. Thermal PS pyrolysis produced mainly light aromatics and PAHs with a lower oligomer content. The PyOil consisted predominantly of gasoline-range hydrocarbons (<C12), while the remaining fraction corresponded to diesel-range compounds (up to C28); heavier hydrocarbons were not detected. The heavy oil fraction included in the PyOil GC–MS analysis was mainly associated with high-boiling aromatic compounds, particularly PAHs (e.g., naphthalene derivatives, fluorene, anthracene derivatives, and acenaphthylene), as well as higher-molecular-weight aromatic oligomeric species such as biphenyls, stilbenes, bibenzyl, and related condensed aromatic compounds.

SSA induced a pronounced shift toward lighter aromatic products (Figure 9a). At a 1:1 ratio, the light aromatic fraction increased to 66.8 wt.% and PAHs decreased to 11.8 wt.%, while BTEX increased from 5.8 wt.% in thermal pyrolysis to 24.58 wt.%, mainly due to benzene increasing to 20.25 wt.% (Tables S6–S9). Styrene remained close to the thermal value, whereas at 1:10 its content increased to 47.23 wt.% with PAHs decreasing to 7.92 wt.%. Thus, SSA suppressed heavy aromatic formation while either favoring light aromatics or preserving styrene depending on catalyst loading, accompanied by an increase in the gasoline-range fraction.

SSC showed a weaker and non-monotonic effect (Figure 9b). At 1:1, styrene decreased to 26.62 wt.% while BTEX increased to 20.07 wt.%, indicating enhanced secondary conversion, although PAH content remained largely unchanged (Tables S10–S13). At 1:4, styrene increased to 42.6 wt.% but PAHs remained relatively high, confirming the weaker ability of SSC to suppress heavy aromatics compared with SSA.

Fe-LSSC produced the strongest redistribution of PyOil toward light aromatics and gasoline-range products (Figure 9c). At 1:1, light aromatics increased from 50.72 wt.% for SSC to 64.25 wt.%, while PAHs decreased from 24.42 to 8.79 wt.% (Tables S14–S17). At 1:2, the gasoline-range fraction reached 82.1 wt.%, compared with 59.3 wt.% for SSC and 69.8 wt.% for thermal PS pyrolysis. These results demonstrate that HCl treatment and Fe impregnation substantially enhanced the ability of SSC-derived material to promote secondary conversion toward lighter aromatics while suppressing PAHs. The effect remained strongly loading-dependent: although 1:2 produced the strongest shift toward light and gasoline-range products, reducing the loading to 1:10 increased styrene to 46.8 wt.% while maintaining a high PAH fraction, indicating that catalyst loading controls the extent of secondary transformation of PS-derived aromatic intermediates.

After multiple reuse cycles, the coked Fe-LSSC/PS 1:4 catalysts (Figure 9d) showed increased oligomer formation and a slight increase in light aromatics compared with both pristine PS and fresh Fe-LSSC/PS 1:4. The regenerated Fe-LSSC-red catalyst produced a hydrocarbon distribution and fuel fraction composition similar to non-catalytic pyrolysis, except for an approximately twofold increase in oligomer content.

### 3.9. Structure–Activity Relationships and Catalytic Pathways

The principal structure–activity insight obtained in this study is that catalytic performance of the SS-derived materials is not governed by total acidity or BET surface area alone, but by the combined effects of surface accessibility, acid–base site distribution, mineral composition, and Fe-containing functionality. Relative to unmodified SSC, acid leaching followed by Fe impregnation increased the BET surface area from 1.41 to 8.04 m^2^ g^−1^ and the pore volume from 0.007 to 0.034 cm^3^ g^−1^, while substantially removing Ca-, P-, S-, and Mg-containing phases. XRD further showed disappearance of gehlenite, CaSO_4_, and calcium phosphate phases after modification, together with enrichment of the residual silicate matrix. Fe incorporation increased the Fe content from 3.72 to 8.92 wt.% and SEM-EDX indicated widespread Fe distribution across the modified surface. At the same time, total NH_3_-TPD acidity decreased from 274 to 167 μmol g^−1^, whereas the acid-site distribution shifted from predominantly strong sites toward a greater relative contribution of medium-strength sites. CO_2_-TPD also indicated formation of a substantially different high-temperature basic-site population. Thus, Fe-LSSC should not be regarded simply as a more acidic SSC, but rather as a chemically restructured multifunctional surface with improved accessibility, redistributed acid–base properties, and Fe-containing functionality.

This distinction is important because Fe-LSSC exhibited substantially stronger secondary conversion than SSC despite having lower total acidity. The enhanced H_2_ formation, higher gasoline-range fraction, and stronger PAH suppression therefore demonstrate that the chemical identity and accessibility of active sites are more important descriptors than total NH_3_ uptake alone. This behavior differs from conventional synthetic catalysts such as zeolites, where catalytic behavior is commonly dominated by a well-defined microporous framework and Brønsted/Lewis acid-site distribution. In contrast, the SS-derived materials investigated here operate through coupled mineral, carbonaceous, acid–base, and Fe-mediated functionalities. The present results therefore show that waste-derived chars can be transformed from chemically heterogeneous solids into more selective catalytic materials through targeted removal and reintroduction of surface functionalities.

Comparing the physicochemical properties of the SS-derived catalysts with their catalytic performance reveals that product selectivity does not depend solely on total acidity. SSC had the highest total acidity among the studied materials (274 μmol g^−1^), far exceeding SSA (175 μmol g^−1^) and Fe-LSSC (167 μmol g^−1^). However, Fe-LSSC caused the most significant changes in both PyGas and PyOil composition. This indicates that the accessibility and specific chemical nature of the active sites, rather than total acidity, control the secondary conversion of PS-derived intermediates. Acid leaching and subsequent Fe impregnation expanded the BET surface area from 1.41 m^2^ g^−1^ (SSC) to 8.04 m^2^ g^−1^ (Fe-LSSC) and increased the pore volume from 0.007 to 0.034 cm^3^ g^−1^. Concurrently, the acid site distribution shifted from mostly strong sites in SSC to a higher proportion of medium-strength sites in Fe-LSSC. Improved mineral surface accessibility, acidity redistribution, and the integration of Fe species therefore work together during PS pyrolysis vapor conversion. This dual role of carbonaceous and mineral functions in SSC aligns with findings that SS-derived char can direct plastic-derived vapors towards aromatic products [51].

H_2_-rich gas production occurs with other transition metal-functionalized SSCs. Wang et al. used municipal SSC-supported Fe/Ni catalysts for toluene reforming and reached an H_2_ concentration of 73.3% under optimized conditions [69]. Although their feedstock, bimetallic Fe–Ni catalyst, and steam-reforming system differ from the PS pyrolysis system, the outcome confirms that transition metals supported on SS-derived carbon can promote hydrogen-generation reactions. The high H_2_ concentration observed for Fe-LSSC suggests a contribution of Fe-containing species to secondary reactions and dehydrogenation during PS pyrolysis, but verification of the precise Fe redox cycle requires in-situ or post-reaction spectroscopy.

Catalyst loading highlights the balance between primary depolymerization and secondary catalytic pathways. At the highest Fe-LSSC loading (1:1), contact between PS vapors and active sites promoted cracking and gas formation. At 1:2, the gasoline-range fraction reached 82.1 wt.% and H_2_ 75.3 vol.%, indicating efficient secondary conversion. At 1:10, styrene increased to 46.8 wt.%, approaching primary PS depolymerization. Thus, higher catalyst-to-polymer ratios shift selectivity from styrene preservation toward dealkylation, cracking, dehydrogenation, and lighter aromatics, whereas lower loadings favor styrene.

These trends agree with the PS–SS co-conversion literature. Xu et al. reported that adding 3 wt.% ZSM-5 during co-pyrolysis of dewatered SS and PS increased aromatic hydrocarbons while reducing wax and slag [70]. Although their system used SS as a co-feedstock and ZSM-5 as a catalyst, both studies show that active solids redirect PS condensables toward lighter aromatics. Here, SS-derived materials provide this upgrading function directly.

The contrast between SSA and SSC shows that BET surface area alone does not determine catalytic activity. Despite its low surface area, SSA increased BTEX and reduced PAHs, indicating catalysis by external mineral phases. SSC possessed the highest total acidity but produced a weaker, non-linear change in oil composition. Its carbon matrix may facilitate adsorption and contact with mineral sites while also retaining aromatic intermediates and promoting condensation. Sun et al. showed that SS-derived char can enhance aromatic production during waste-plastic pyrolysis, supporting the combined role of carbonaceous and inorganic SSC components [51].

Carbon deposition helps explain deactivation. Lu et al. reported that coke formation during toluene cracking over SSC reduced BET surface area from 74.213 to 51.782 m^2^ g^−1^ under N_2_, demonstrating pore blockage during conversion [71]. CO_2_ and steam mitigated this loss through carbon gasification. Although the present experiments used neither gas, aromatic PS intermediates can condense, making coke deposition and pore blockage plausible.

Reuse results support this interpretation. Across Fe-LSSC cycles, H_2_ decreased while CH_4_ rose to 42.3 vol.%, whereas PyOil increased from 59.06 wt.% after the first reuse to 82.73 wt.% after the third. This indicates suppression of secondary cracking and dehydrogenation, leaving more primary vapors condensable. Carbon deposition on Fe and acid–base sites can explain both declining H_2_ and increasing liquid recovery, consistent with coke-induced pore loss reported previously [71].

Thermal regeneration restored H_2_ to 68.3 vol.% and reduced CH_4_ to 13.3 vol.%, indicating partial recovery after coke removal. However, regenerated Fe-LSSC did not fully reproduce fresh-catalyst performance, suggesting irreversible changes in Fe speciation, mineral phases, pore structure, or active-site distribution. The formation of crystalline Fe_3_O_4_ in the spent catalyst indicates that the Fe-containing fraction undergoes structural and/or redox transformation during pyrolysis, although the specific oxidation-state changes cannot be resolved by XRD alone.

Overall, the observed behavior reflects mineral acid–base sites, surface accessibility, carbon adsorption, and Fe-mediated secondary reactions. Acid leaching alters the residual mineral framework, while Fe impregnation introduces redox-active functionality. Previous reports of aromatic upgrading by SSC [51], coke-induced deactivation during aromatic cracking [71], and enhanced H_2_ production over metal-loaded sludge char [69] support these observations. Together, these findings explain why Fe-LSSC shows stronger selectivity than SSC despite lower total acidity and demonstrate that SS-derived catalysts can tune PS pyrolysis toward liquid aromatics or H_2_-rich gas. Although these multi-cycle product trends provide evidence of progressive catalyst deactivation and partial regeneration, dedicated BET, SEM-EDX, TPD, and XRD characterization was not performed after each individual reuse cycle; therefore, the specific evolution of surface area, acidity/basicity, Fe dispersion, and coke coverage across consecutive cycles cannot be quantitatively resolved from the present data.

From a process-scale perspective, the waste-derived origin of Fe-LSSC should not by itself be interpreted as evidence of lower cost or environmental impact relative to conventional catalysts. Its preparation involves several energy- and chemical-intensive operations, including SSC production at 900 °C under inert atmosphere, leaching with 3 M HCl at a 1:20 solid-to-liquid ratio for 5 h at 80 °C, washing and drying, Fe(NO_3_)_3_·9H_2_O impregnation in isopropanol, a further 5 h treatment at 80 °C, drying at 105 °C, and final calcination at 900 °C for 1 h. Consequently, the practical burden of Fe-LSSC production includes thermal energy, inert gas, acid, washing water, solvent, and Fe precursor consumption, together with treatment or recovery of the resulting liquid streams. The magnitude of this burden depends strongly on the system boundary. If SSC is produced specifically for catalyst manufacture, the energy required for initial carbonization must be assigned to the catalyst; however, if SSC is available as a residue or co-product of an existing SS pyrolysis process, only the additional modification steps would contribute directly to catalyst preparation. Industrial implementation would therefore benefit from heat integration, acid recycling, solvent recovery, and reduced liquid-to-solid ratios.

Catalyst loading is an additional scale-up consideration. The 1:1 catalyst-to-PS ratio employed in this study represents a high-loading condition selected to reveal the maximum catalytic response and should not be regarded as an optimized industrial ratio. Lower catalyst loadings produced substantially different product distributions and, in some cases, higher liquid and styrene yields, indicating that catalyst inventory must be selected according to the desired product slate. High catalyst consumption would increase catalyst preparation, handling, regeneration, and replacement requirements, potentially offsetting the economic advantage of using a waste-derived precursor. Reuse tests showed that Fe-LSSC retained catalytic functionality, while thermal regeneration partially restored its H_2_-promoting behavior; however, repeated regeneration also introduces an energy penalty. Therefore, the present results establish catalytic feasibility and tunability rather than a demonstrated net economic or environmental advantage. A complete assessment would require TEA and LCA incorporating catalyst lifetime, regeneration frequency, chemical recovery, thermal integration, and product value.

Conventional turnover number (TON) and turnover frequency (TOF) were not calculated for the present system because a unique population of catalytically active sites cannot be rigorously defined. NH_3_-TPD quantifies total acid sites, but the observed activity also reflects basic and mineral sites, carbonaceous surfaces, Fe-containing phases, and differences in site accessibility. Indeed, Fe-LSSC produced substantially stronger product redistribution than SSC despite its lower total acidity, demonstrating that total NH_3_ uptake cannot be equated with the number of reaction-specific active sites. In addition, PS undergoes extensive thermal depolymerization in the absence of catalyst; therefore, assigning total PS conversion to catalytic turnover would overestimate TON. The non-isothermal experimental protocol further prevents determination of a meaningful steady-state TOF because temperature, PS vapor generation, reactant concentration, and catalyst state vary throughout the experiment. Accordingly, catalytic performance is evaluated here through changes in product yield and selectivity relative to thermal pyrolysis, loading dependence, and catalyst reuse/deactivation/regeneration behavior.

## 4. Conclusions

This study demonstrated the catalytic potential of SS-derived materials for the conversion of polystyrene through pyrolysis and highlighted the strong influence of catalyst composition and modification on product distribution. SSA, SSC, and Fe-LSSC exhibited distinct catalytic behaviors, reflecting differences in their mineral composition, carbonaceous structure, and surface acidity. Thermal analysis showed that the incorporation of SS-derived catalysts modified the degradation pathway of PS, with the most pronounced shift in the DTG profile observed for Fe-LSSC at a 1:1 ratio.

The product distribution was strongly dependent on both catalyst type and loading. SSA favored the formation of lighter aromatic compounds and suppressed the formation of heavier aromatic products, while SSC showed a weaker and non-monotonic effect. Fe-LSSC exhibited the strongest catalytic influence, substantially increasing gas formation at high catalyst loading and promoting the transformation of PS-derived vapors toward lighter aromatic and gasoline-range products. In particular, the Fe-LSSC/PS ratio of 1:2 resulted in an H_2_ concentration of 75.3 vol.% in the pyrolysis gas and an 82.1 wt.% gasoline-range fraction in the PyOil. These effects indicate that acid leaching followed by Fe incorporation can substantially modify the catalytic functionality of SSC and alter the pathways of secondary conversion of PS-derived vapors. Although the use of waste-derived PS provides greater practical relevance than experiments based exclusively on virgin polymer, further studies using more heterogeneous PS waste streams containing representative fillers, dyes, stabilizers, and other additives are required to evaluate the robustness of the catalytic system under realistic waste-processing conditions.

Catalyst reuse demonstrated that Fe-LSSC retained catalytic functionality, although progressive changes in gas composition indicated partial deactivation, most likely associated with carbon deposition. Regeneration partially restored the gas-phase distribution, confirming the potential for catalyst regeneration. Overall, the results demonstrate that SS-derived materials can serve as low-cost and chemically tunable catalysts for polystyrene pyrolysis. More importantly, the comparison of SSA, SSC, and Fe-LSSC shows that controlled modification of SS-derived materials provides a means of tailoring the balance between liquid production, gas formation, and the composition of aromatic products, thereby supporting the simultaneous valorization of SS and plastic waste.

## Figures and Tables

**Figure 1:**
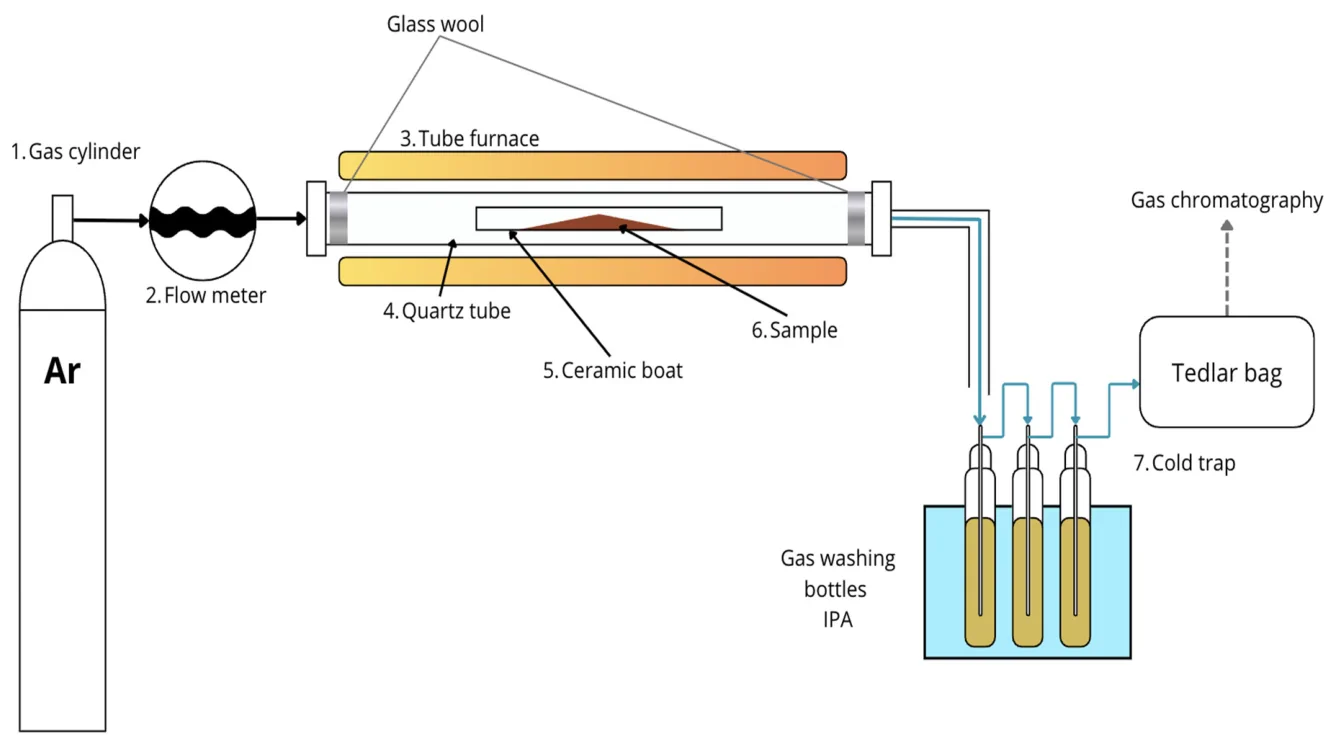
Experimental setup of the horizontal semi-batch tubular reactor used for in-situ catalytic slow pyrolysis of PS.

**Figure 2:**
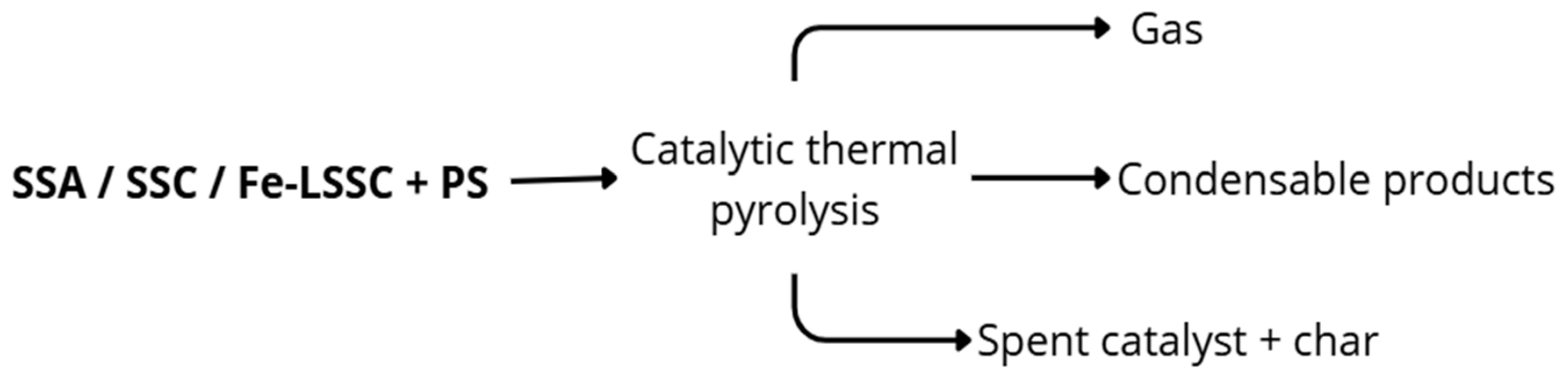
Mass balance structure of PS.

**Figure 3:**
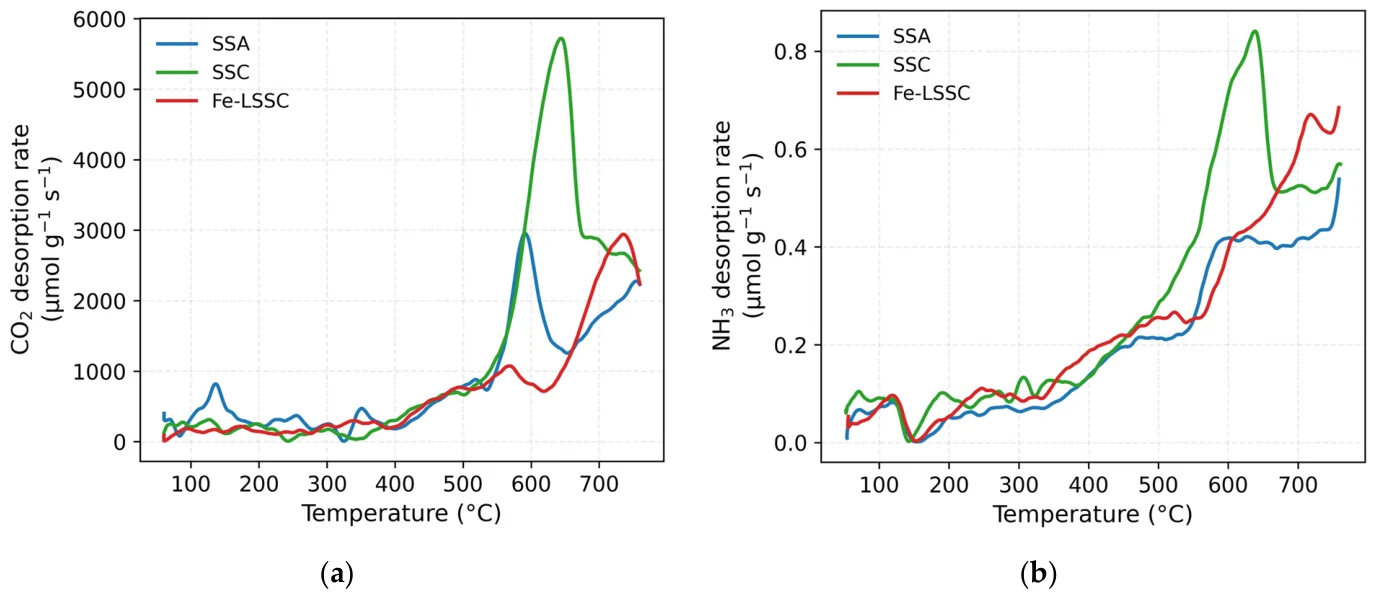
TPD analysis of SS-derived catalysts: (**a**) CO_2_-TPD. (**b**) NH_3_-TPD.

**Figure 4:**
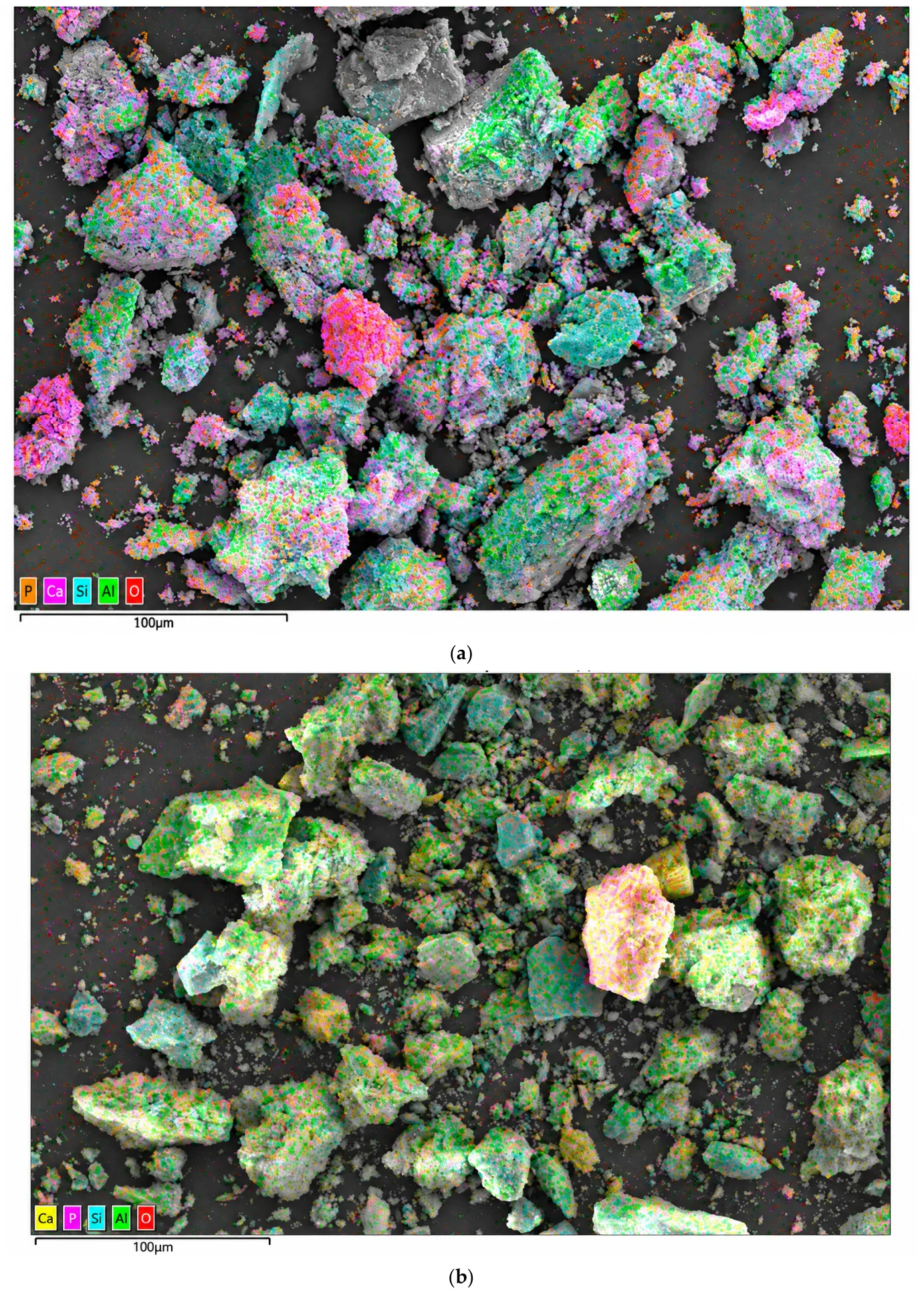

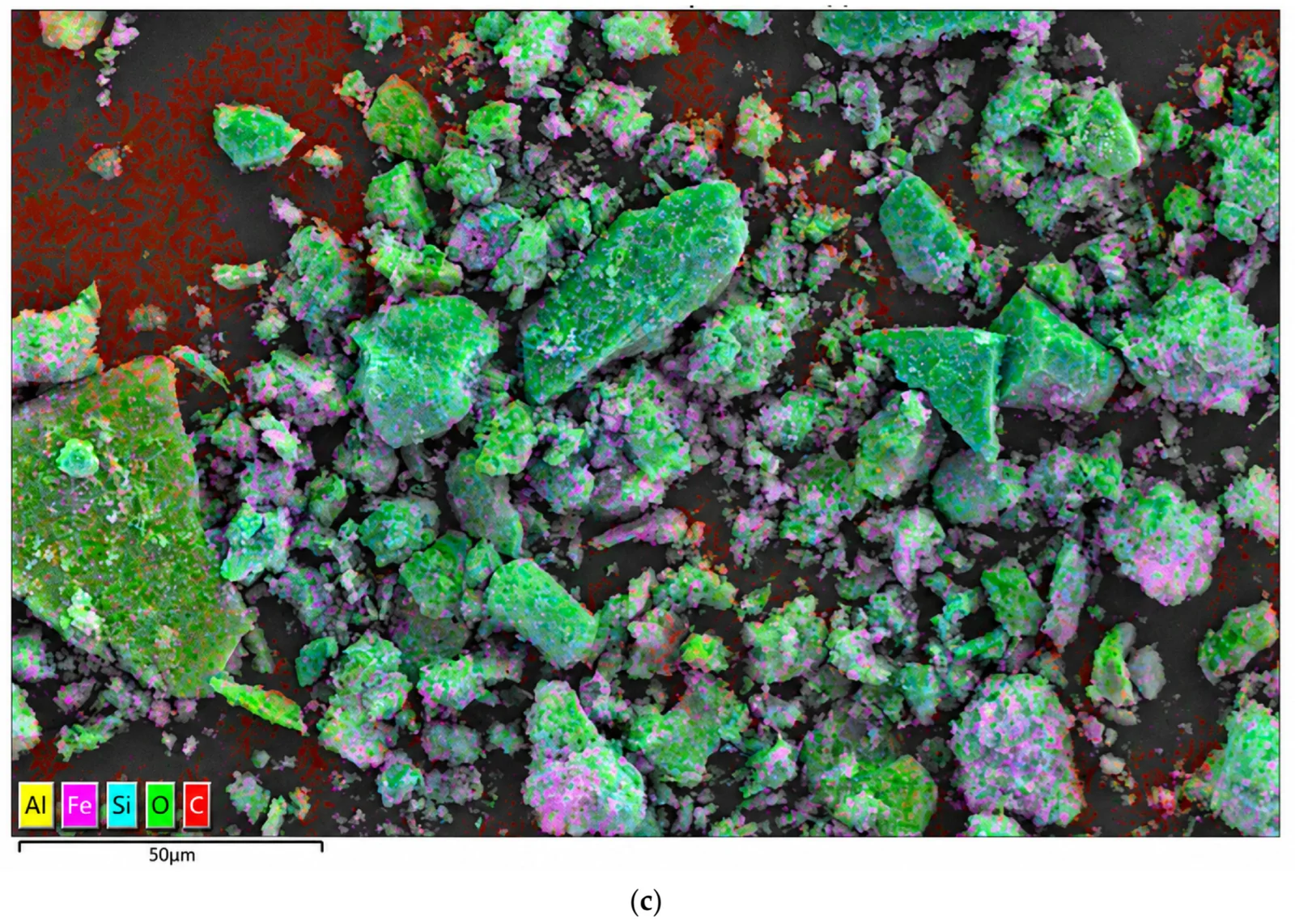
SEM-EDX analysis of SS-derived catalysts: (**a**) SSA. (**b**) SSC. (**c**) Fe-LSSC.

**Figure 5:**
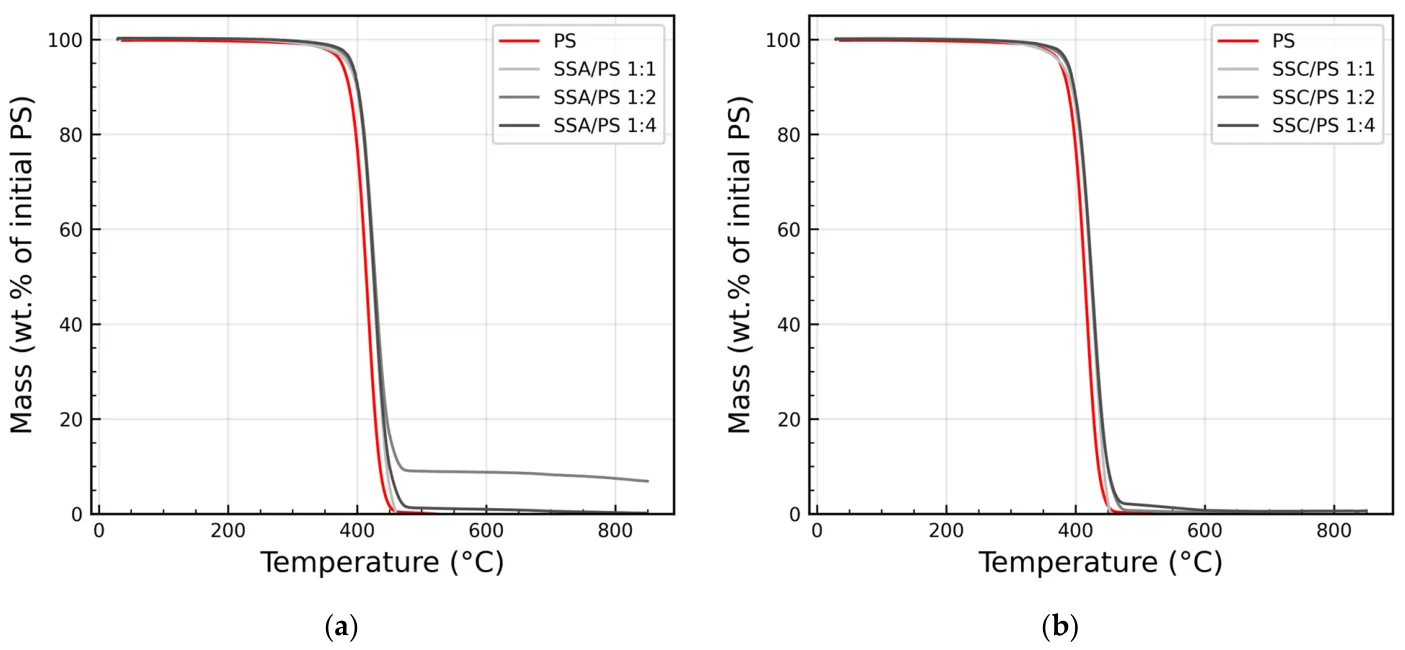

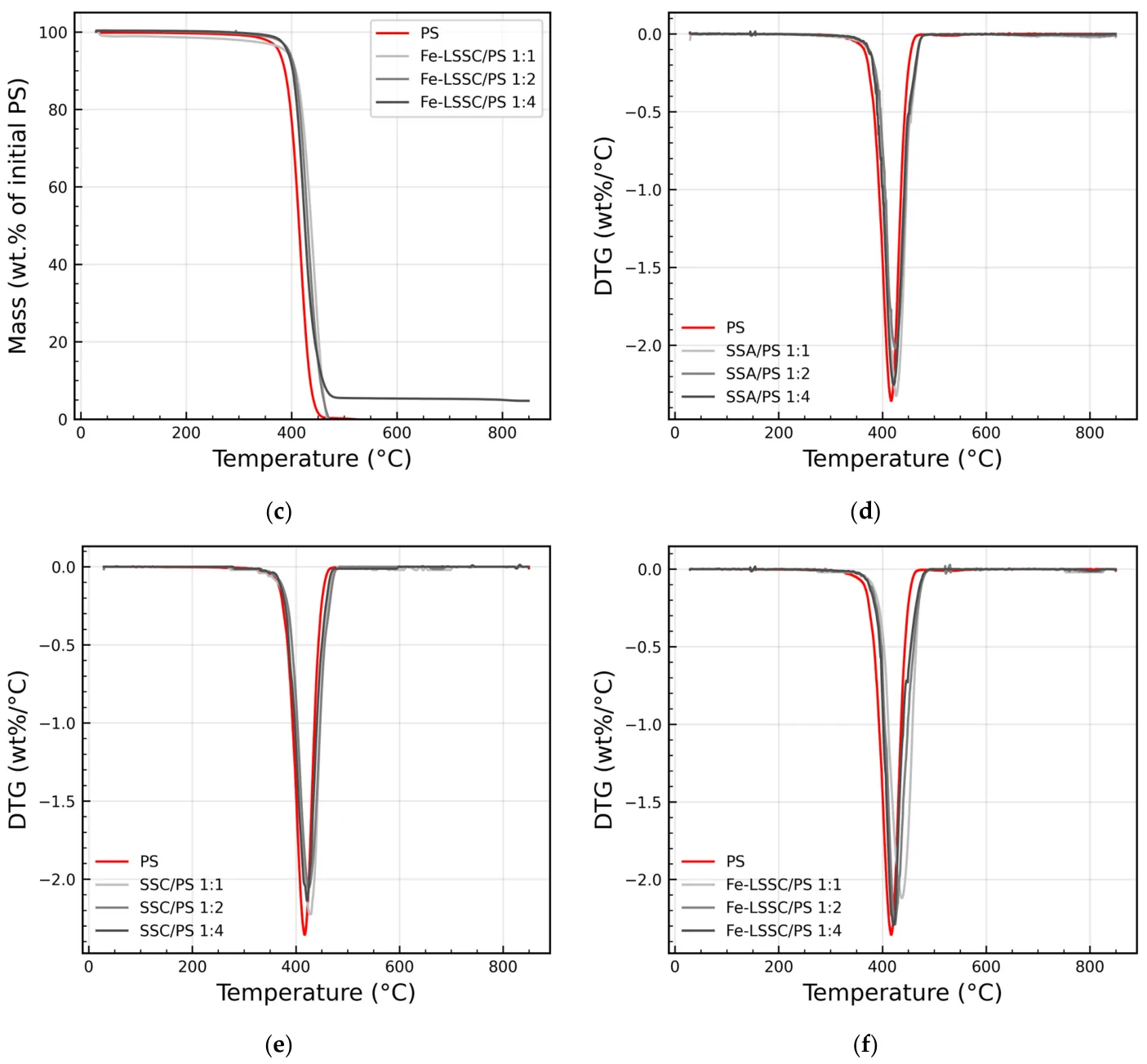
Normalized TGA and DTG curves of PS with SS-derived catalysts on an initial PS mass basis: (**a**) TGA: PS with SSA. (**b**) TGA: PS with SSC. (**c**) TGA: PS with Fe-LSSC. (**d**) DTG: PS with SSA. (**e**) DTG: PS with SSC. (**f**) DTG: PS with Fe-LSSC.

**Figure 6:**
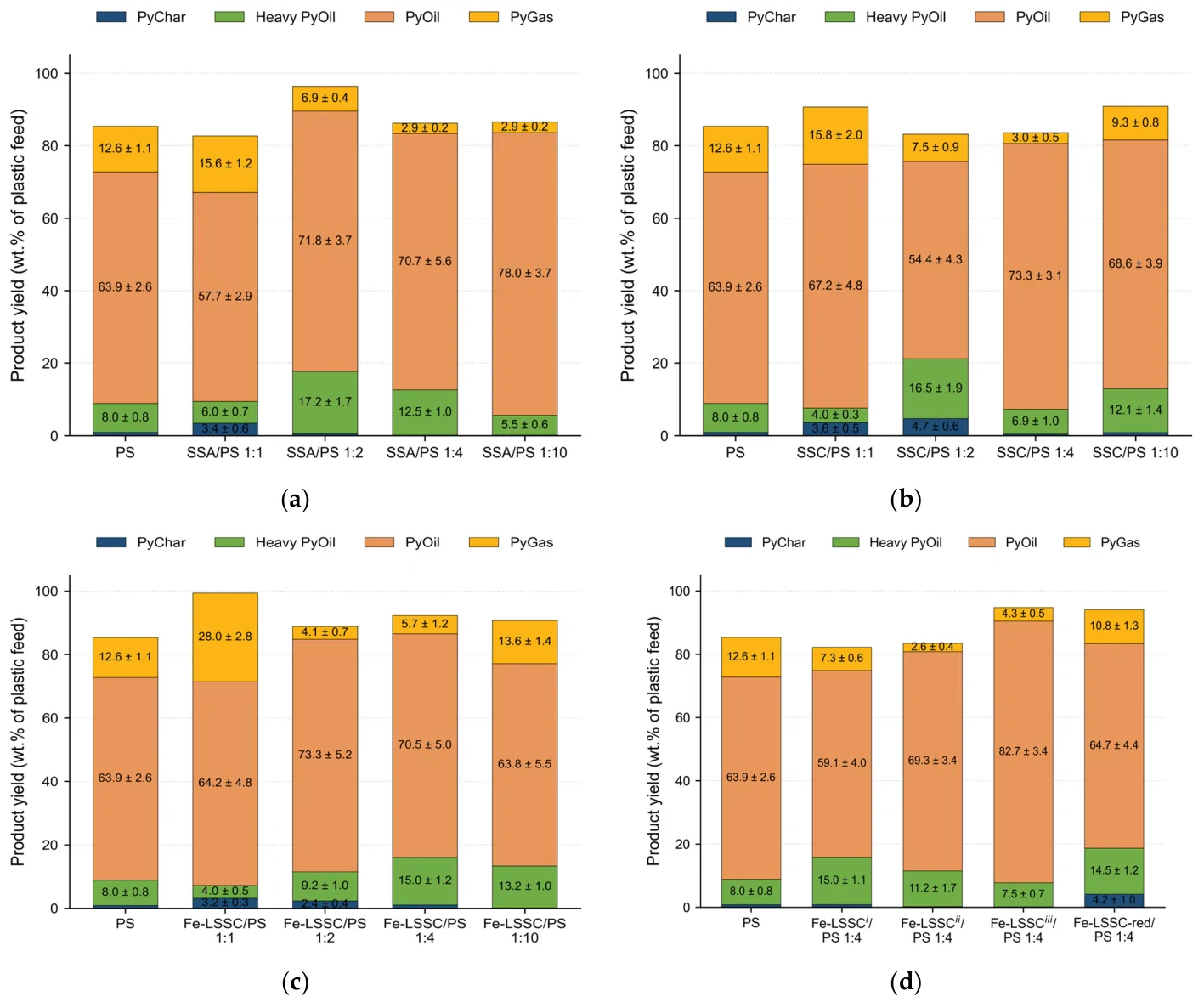
Balance of catalytic pyrolysis of PS (wt.%): (**a**) SSA. (**b**) SSC. (**c**) Fe-LSSC. (**d**) Fe-LSSC reuse cycles and thermal regeneration at Fe-LSSC/PS = 1:4. Error bars represent standard deviations (SD) from triplicate experiments (n = 3).

**Figure 7:**
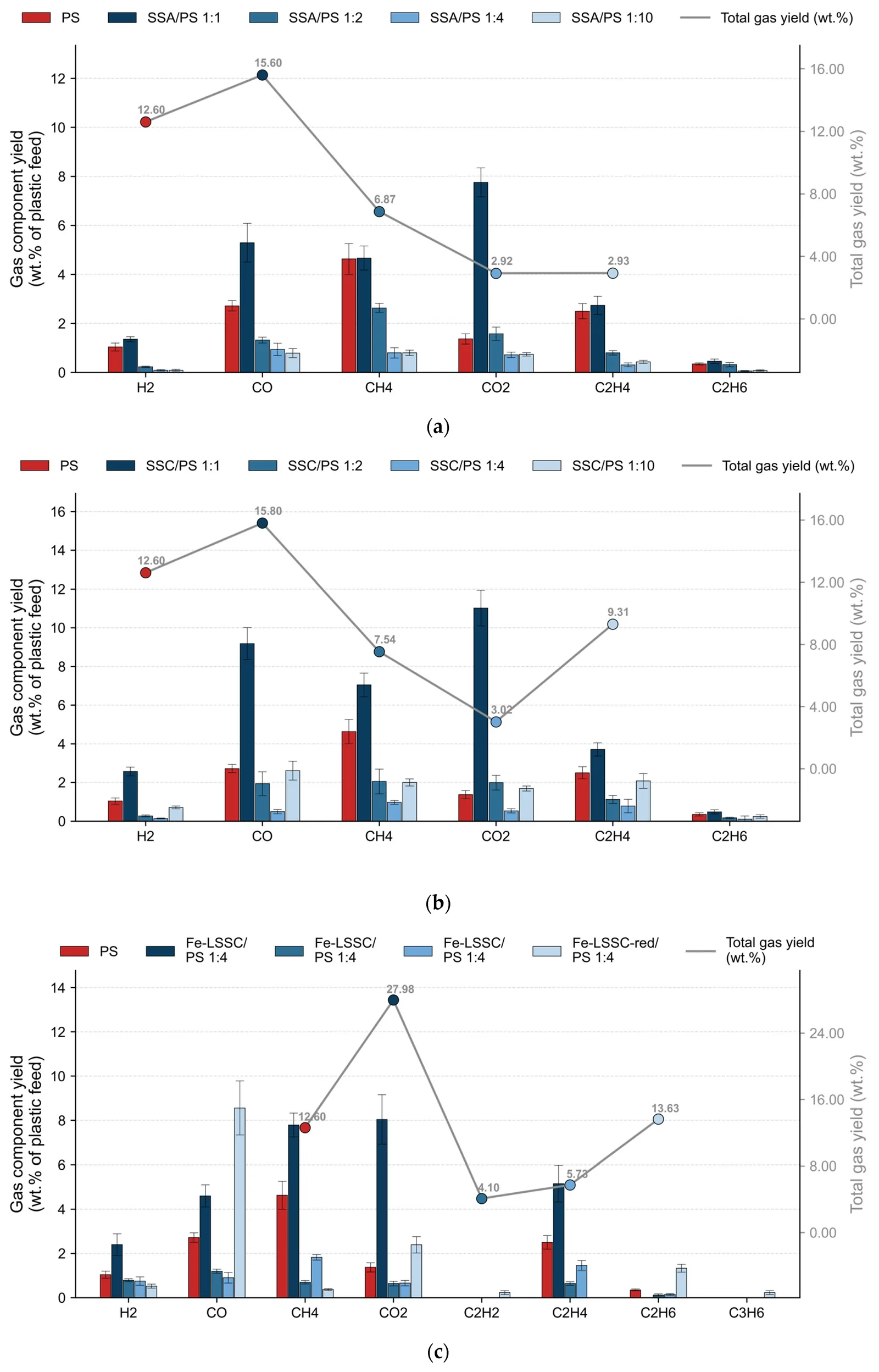

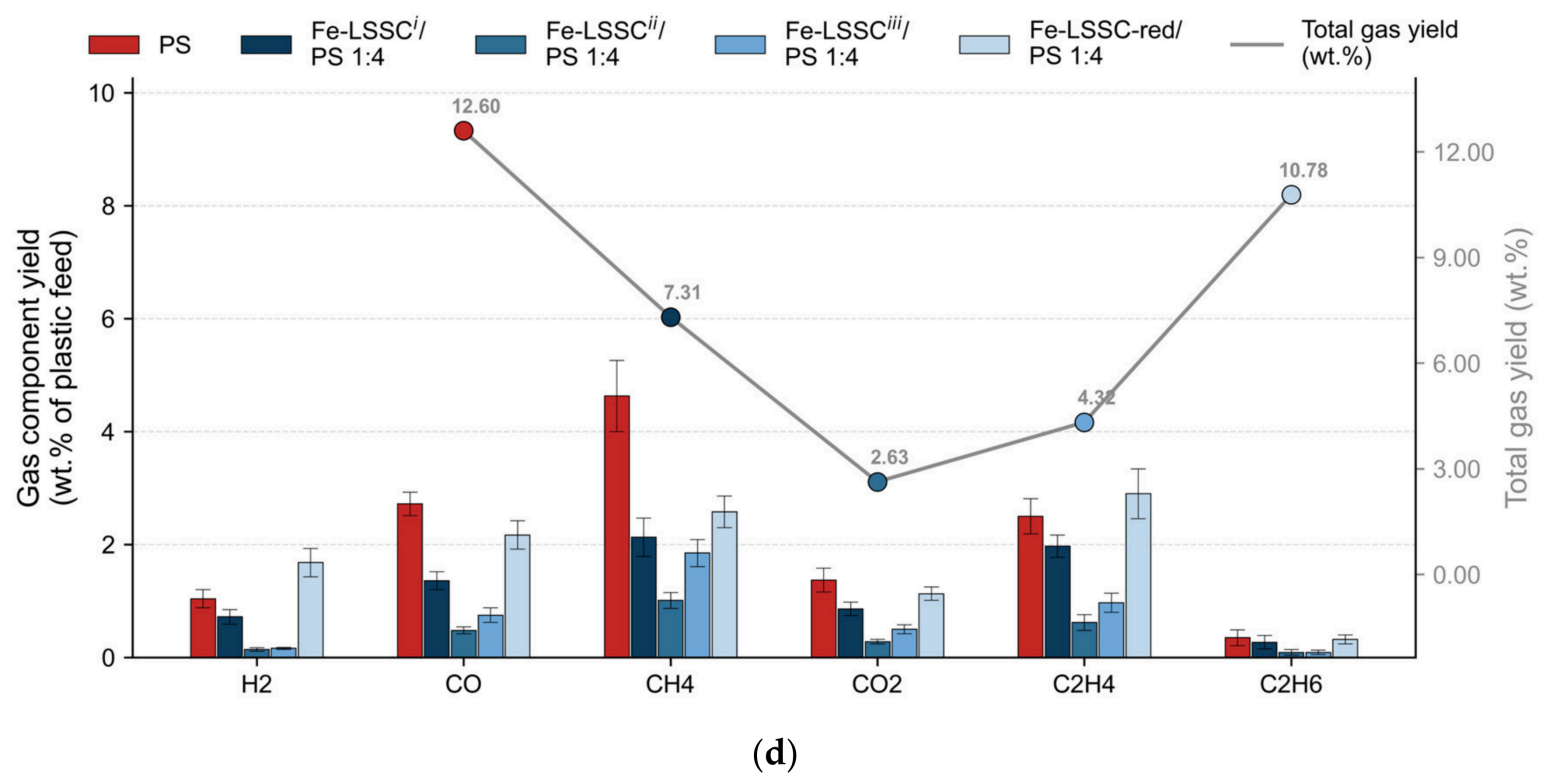
Yield of PyGas and its components (wt.% of plastic feed): (**a**) from SSA/PS; (**b**) from SSC/PS; (**c**) from Fe-LSSC/PS. (**d**) PyGas component yields during Fe-LSSC reuse cycles and after thermal regeneration. Error bars represent standard deviations (SD) from triplicate experiments (n = 3).

**Figure 8:**
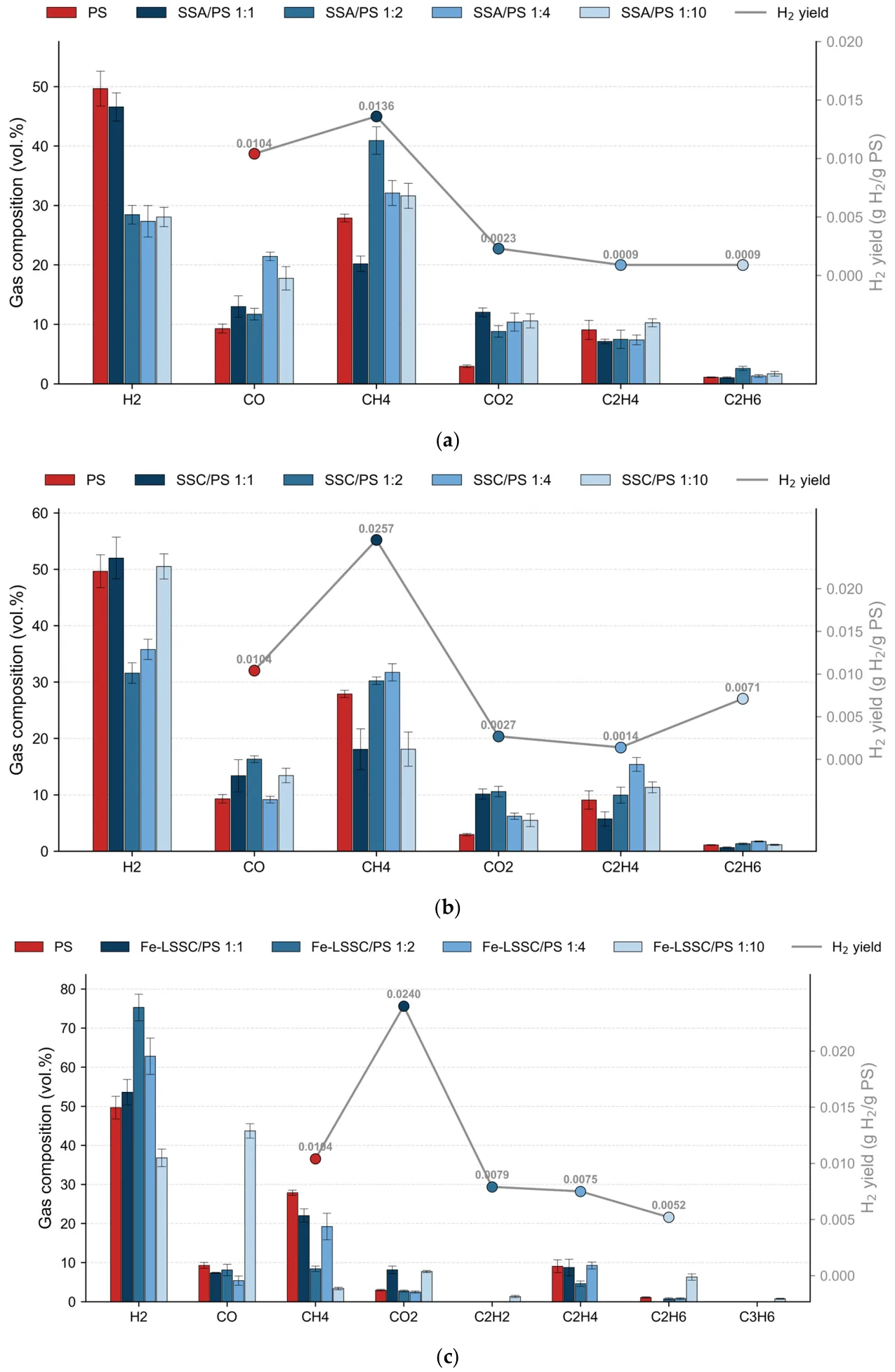

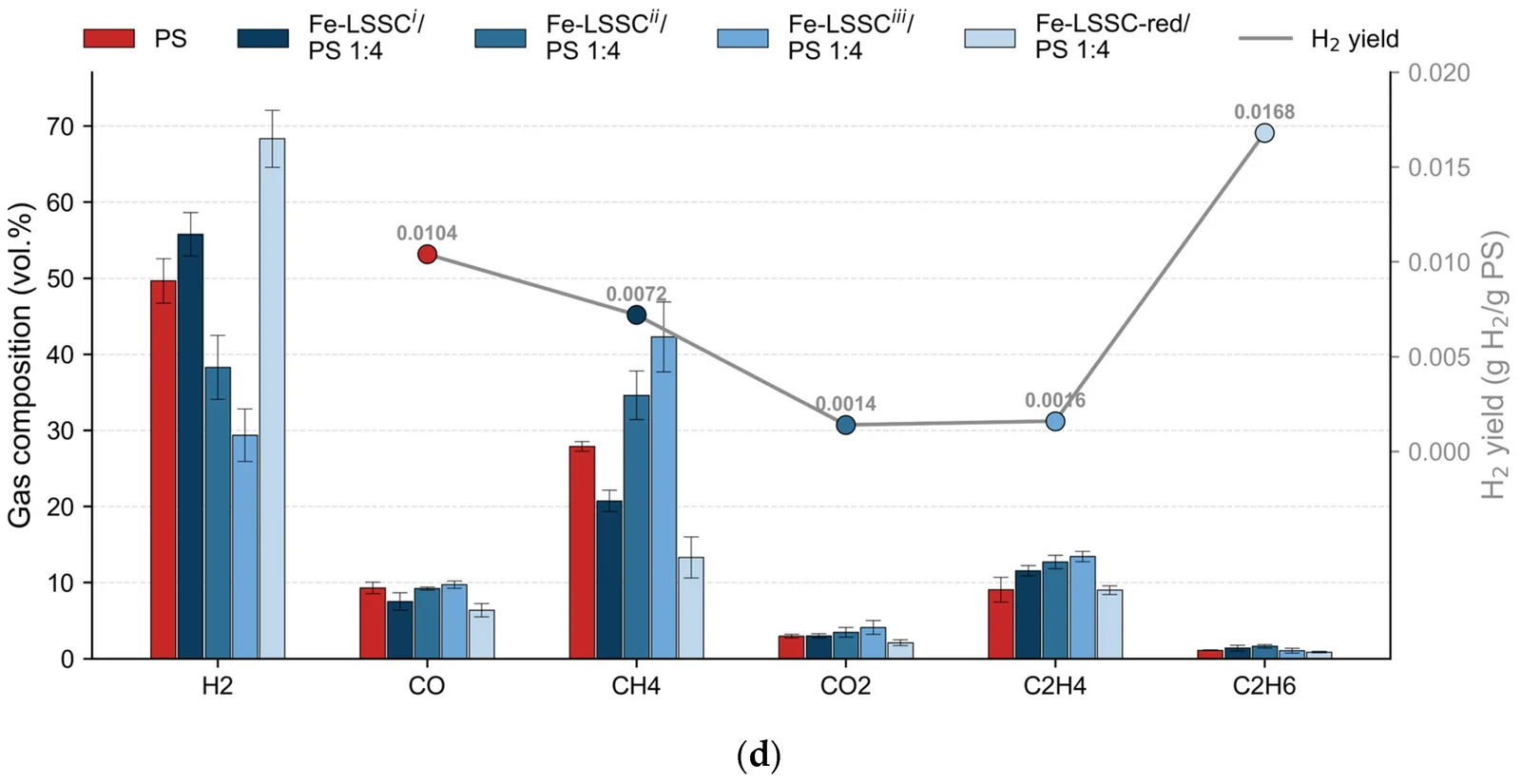
Composition of PyGas (vol.%): (**a**) from SSA/PS; (**b**) from SSC/PS; (**c**) from Fe-LSSC/PS. (**d**) PyGas composition during Fe-LSSC reuse cycles and after thermal regeneration. Error bars represent standard deviations (SD) from triplicate experiments (n = 3).

**Figure 9:**
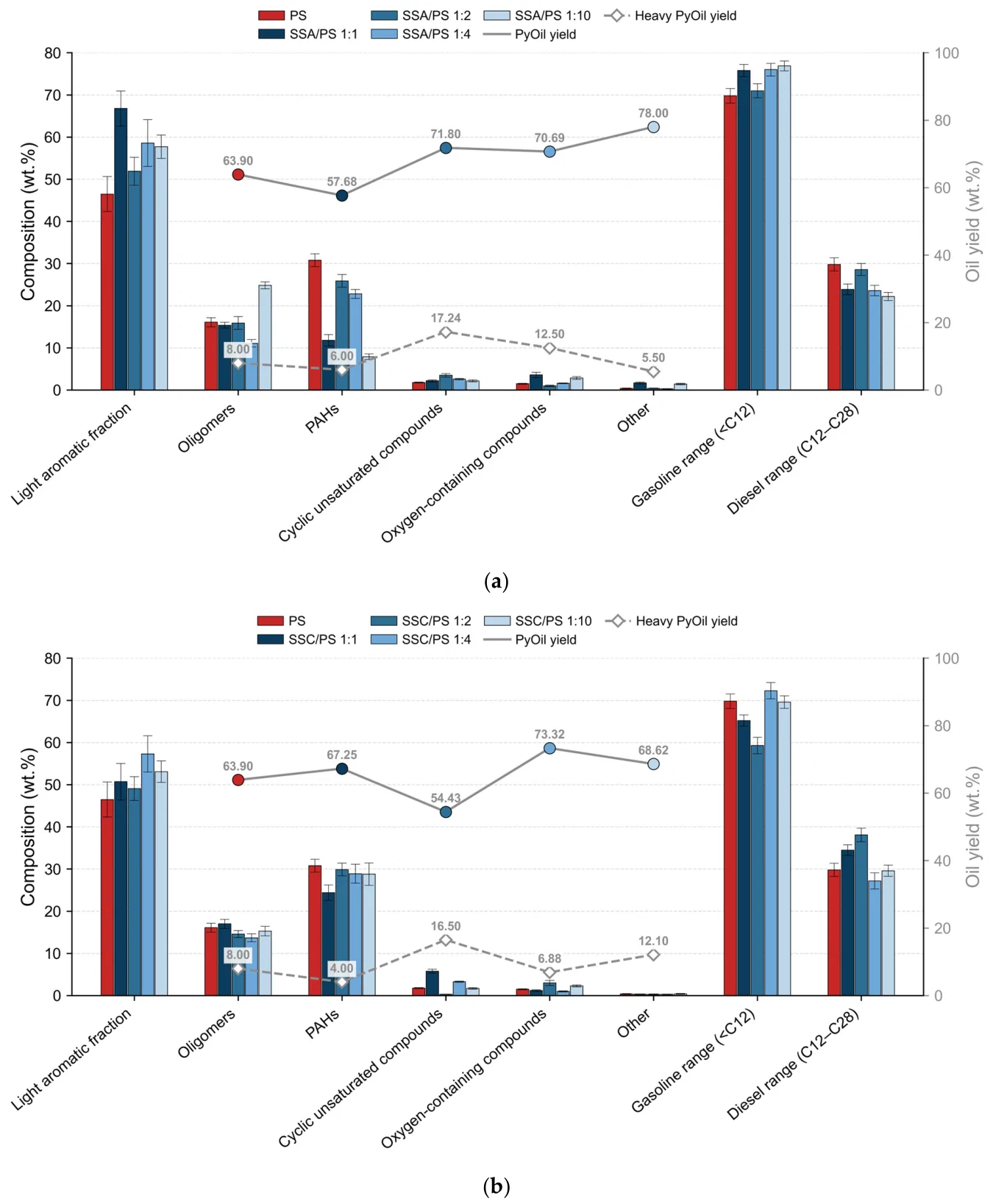

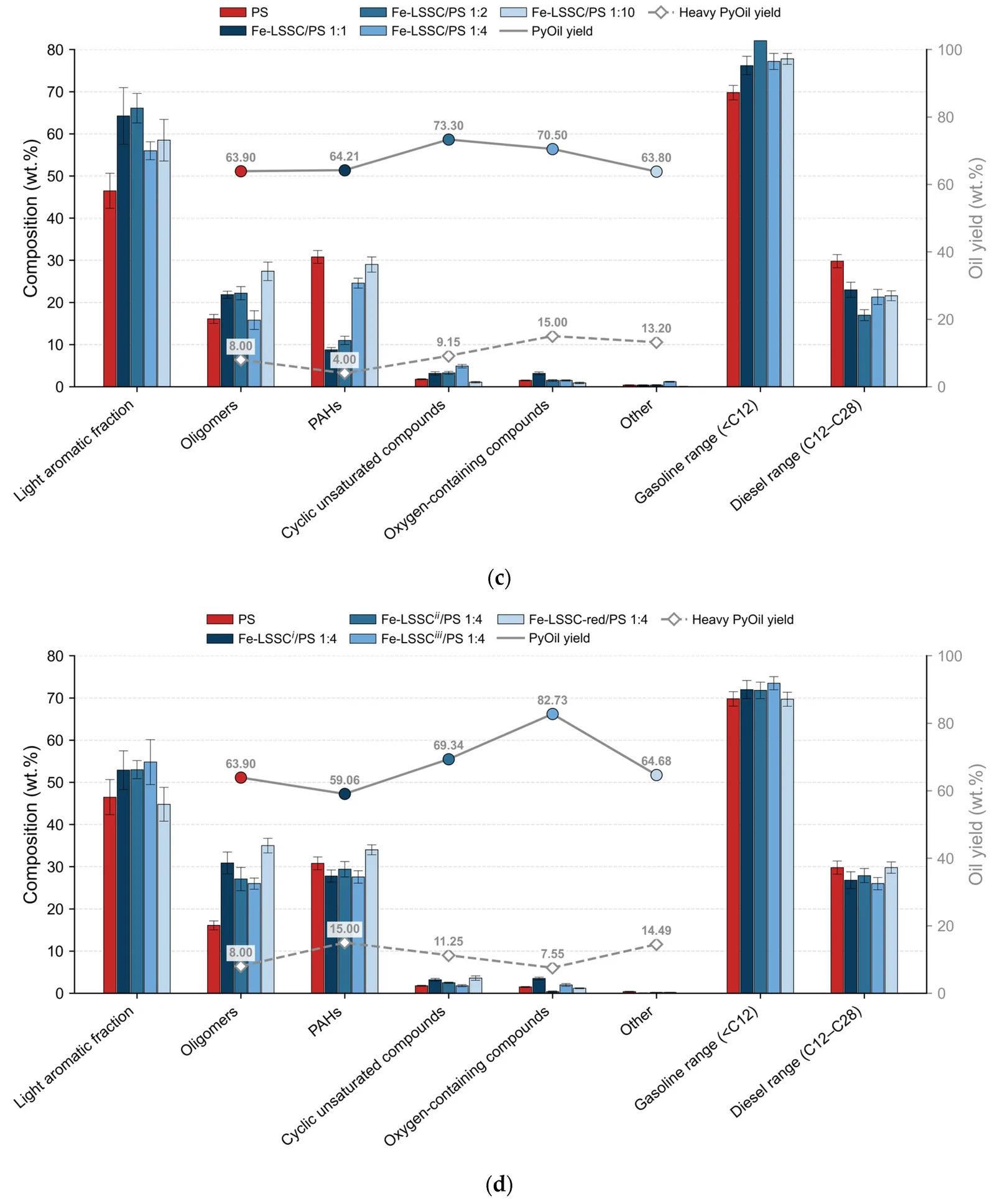
GC-MS analysis summary of catalytic pyrolysis of PS: (**a**) SSA catalytic PyOil. (**b**) SSC catalytic PyOil. (**c**) Fe-LSSC catalytic PyOil. (**d**) PyOil composition during Fe-LSSC reuse cycles and after thermal regeneration. Error bars represent standard deviations (SD) from triplicate experiments (n = 3).

**Table 1:** Proximate and elemental analyses of PS and raw sewage sludge.

Name	Proximate Analysis (wt.%)			Elemental Analysis (wt.%)				
	Volatiles	Ash Content	Fixed Carbon	C	H	N	S	O
PS	-	-	-	91.25 ± 2.74	8.75 ± 0.10	0.03	0.00	0.00
SS	34.2 ± 0.47	60.7 ± 1.26	5.2 ± 0.11	17.80 ± 0.43	2.80 ± 0.07	2.04 ± 0.05	1.07 ± 0.03	15.63 ± 0.38

**Table 2:** XRD analysis results of SSA, SSC, and Fe-LSSC catalysts.

	Analyte	SSA, wt.%	SSC, wt.%	Fe-LSSC, wt.%
Quartz	SiO_2_	25.4	26.0	58.7
Albite	NaAlSi_3_O_8_	23.4	21.8	19.0
Maghemite	Fe_2_O_3_	5.7	6.2	
Gehlenite	Ca_2_Al_2_SiO_7_	20.4	18.6	
Calcium Sulfate	CaSO_4_	17.0	14.7	
Calcium Phosphate	Ca_4_P_2_O_9_	8.0	9.2	
Calcium Oxide	CaO		3.5	
Microcline	(K,Na)AlSi_3_O_8_			22.3

**Table 3:** Analysis of SS-derived material surface characteristics.

	SSA			SSC			Fe-LSSC		
	BET, m^2^·g^−1^	Pore V, cm^3^·g^−1^	Pore S, nm	BET, m^2^·g^−1^	Pore V, cm^3^·g^−1^	Pore S, nm	BET, m^2^·g^−1^	Pore V, cm^3^·g^−1^	Pore S, nm
SS	0.94	0.004	1.06	1.41	0.007	1.06	8.04	0.034	1.38

**Table 4:** Analysis of SS-derived material acidic characteristics.

Sample	Weak Acidic Sites, μmol·g^−1^	Medium Acidic Sites, μmol·g^−1^	Strong Acidic Sites, μmol·g^−1^	Total Number of Acidic Sites, μmol·g^−1^
SSA	38	22	115	175
SSC	18	64	192	274
Fe-LSSC	33	74	60	167

## Data Availability

The original contributions presented in this study are included in the article/Supplementary Materials. Further inquiries can be directed to the corresponding author.

## Supplementary Materials

The following supporting information can be downloaded at https://www.mdpi.com/article/10.3390/polym18192372/s1: Figure S1. Preparation process of an SS-derived catalyst. Figure S2. Heating program used for the slow pyrolysis process. Figure S3. XRD analysis of SS-derived catalysts: (a) Sewage sludge ash, wt.%. (b) Sewage sludge char, wt.%. (c) Acid-leached Fe-impregnated sewage sludge char, wt.%. Figure S4. SEM-EDX analysis of SS-derived catalysts: (a) Sewage sludge ash. (b) Sewage sludge char. (c) Acid-leached Fe-impregnated sewage sludge char. Table S1. Summary of the XRF analysis of the catalysts (wt.%). Table S2. XRD analysis results of sewage sludge ash catalyst before and after PS pyrolysis experiments. Table S3. XRD analysis results of sewage sludge char catalyst before and after PS pyrolysis experiments. Table S4. XRD analysis results of acid-leached Fe-impregnated sewage sludge char catalyst before and after PS pyrolysis experiments. Table S5. GC–MS analysis results for the oil produced during thermal pyrolysis of PS. Table S6. GC–MS analysis results for the oil produced during catalytic pyrolysis of sewage sludge ash with PS at a catalyst-to-PS ratio of 1:1. Table S7. GC–MS analysis results for the oil produced during catalytic pyrolysis of sewage sludge ash with PS at a rate of 1:2. Table S8. GC–MS analysis results for the oil produced during catalytic pyrolysis of sewage sludge ash with PS at a rate of 1:4. Table S9. GC–MS analysis results for the oil produced during catalytic pyrolysis of sewage sludge ash with PS at a catalyst-to-PS ratio of 1:10. Table S10. GC–MS analysis results for the oil produced during catalytic pyrolysis of sewage sludge char with PS at a catalyst-to-PS ratio of 1:1. Table S11. GC–MS analysis results for the oil produced during catalytic pyrolysis of sewage sludge char with PS at a rate of 1:2. Table S12. GC–MS analysis results for the oil produced during catalytic pyrolysis of sewage sludge char with PS at a rate of 1:4. Table S13. GC–MS analysis results for the oil produced during catalytic pyrolysis of sewage sludge char with PS at a catalyst-to-PS ratio of 1:10. Table S14. GC–MS analysis results for the oil produced during catalytic pyrolysis of acid-leached Fe-impregnated sewage sludge char with a PS rate of 1:1. Table S15. GC–MS analysis results for the oil produced during catalytic pyrolysis of acid-leached Fe-impregnated sewage sludge char with a PS rate of 1:2. Table S16. GC–MS analysis results for the oil produced during catalytic pyrolysis of acid-leached Fe-impregnated sewage sludge char with a PS rate of 1:4. Table S17. GC–MS analysis results for the oil produced during catalytic pyrolysis of acid-leached Fe-impregnated sewage sludge char with a PS rate of 1:10. Table S18. GC–MS analysis results for the oil produced during catalytic pyrolysis of acid-leached Fe-impregnated sewage sludge char after one cycle of use with a PS rate of 1:4. Table S19. GC–MS analysis results for the oil produced during catalytic pyrolysis of acid-leached Fe-impregnated sewage sludge char after two cycles of use with a PS rate of 1:4. Table S20. GC–MS analysis results for the oil produced during catalytic pyrolysis of acid-leached Fe-impregnated sewage sludge char after three cycles of use with a PS rate of 1:4. Table S21. GC–MS analysis results for the oil produced during catalytic pyrolysis of acid-leached Fe-impregnated sewage sludge char after regeneration at a catalyst-to-PS ratio of 1:4. Table S22. One-way ANOVA and Tukey’s HSD analysis of PyGas yield and composition presented in Figure 7 and Figure 8. Values are mean ± SD from three independent experiments (n = 3). Different lowercase letters within the same row indicate statistically significant differences according to Tukey’s HSD test (*p* < 0.05); values sharing at least one letter are not significantly different.

## Abbreviations

The following abbreviations are used in this manuscript:

SS	Sewage sludge
SSA	Sewage sludge ash
SSC	Sewage sludge char
LSSA	Leached sewage sludge ash
LSSC	Leached sewage sludge char
Fe-LSSC	Fe-impregnated leached sewage sludge char
PS	Polystyrene
FCC	Fluid catalytic cracking
PyOil	Pyrolysis oil
PyGas	Pyrolysis gas
PyChar	Pyrolysis char
XRD	X-ray diffraction
XRF	X-ray fluorescence
SEM	Scanning electron microscopy
BET	Brunauer–Emmett–Teller
TPD	Temperature-programmed desorption
TGA	Thermogravimetric analysis
DTG	Differential thermogravimetry
GC-MS	Gas chromatography–mass spectrometry
GC-TCD	Gas chromatography thermal conductivity detector
PAHs	Polycyclic aromatic hydrocarbons
