# Supplementary material for: In-Situ Catalytic Pyrolysis of Polystyrene with Sewage Sludge-Derived Catalysts: Sewage Sludge Ash, Sewage Sludge Char, and Fe-Impregnated Acid-Leached Sewage Sludge Char
Source: Polymers (Basel). 2026 Sep 29;18(19):2372. doi: 10.3390/polym18192372 (PMC13666879; doi:10.3390/polym18192372)
Supplement: Supplementary file 1 [file polymers-18-02372-s001.zip › polymers-4560890-supplementary.pdf]

polymers

MDPI

Supplementary Materials: In-Situ Catalytic Pyrolysis of Polystyrene with Sewage Sludge-Derived Catalysts: Sewage Sludge Ash, Sewage Sludge Char, and Fe-Impregnated Acid-Leached Sewage Sludge Char

Fedor Pogorov [1], Diyar Tokmurzin [2,*], Yelena Vitoshnova [1], Nazar Zabara [1], Anara Omarova [1], Olga Ibragimova [1], Aigerim Kaiaidarova [1], Sergey Nechipurenko [1], Sergey Efremov [1], Grigoriy Mun [1] and Mukhambetkali Burkitbayev [1]

[1] Center of Physical-Chemical Methods of Research and Analysis, Farabi University, Almaty 050012, Kazakhstan; fpogorov@gmail.com (F.P.); helenvit4@gmail.com (Y.V.); nzr.zabara@gmail.com (N.Z.); omarova@cfhma.kz (A.O.); aigerim_ko@list.ru (A.K.); nechipurenkos@mail.ru (S.N.); efremsa@mail.ru (S.E.); grigoriy.mun@gmail.com (G.M.); mukhambetkali.burkitbayev@kaznu.edu.kz (M.B.)
[2] Laboratory for Chemical Technology, Ghent University, Industriele Scheikunde, Technologiepark-Zwijnaarde 125, 9052 Ghent, Belgium
* Correspondence: diyar.tokmurzin@ugent.be

Table S1. Summary of the XRF analysis onto the catalyst (wt. %).

Analyte	SSA	SSC	Fe-LSSC
O	47.584	46.033	49.375
Na	0.709	0.708	0.483
Mg	1.311	1.352	0.674
Al	5.014	5.232	3.109
Si	17.797	17.981	24.539
P	3.451	3.022	0.152
S	1.575	1.533	0.145
Cl	0.060	0.081	1.974
K	0.755	0.809	0.959
Ca	12.703	13.172	1.082
Ti	0.817	0.835	1.021
Cr	0.078	0.061	0.045
Mn	0.045	0.039	0.031
Fe	3.413	3.721	8.918
Ni	0.018	0.011	
Cu	0.059	0.035	0.024
Zn	0.114	0.131	0.093
Ga		0.003	
Rb	0.004	0.006	0.006
Sr	0.019	0.021	0.007
Zr	0.007	0.009	0.011
Nb		0.003	

Table S2. XRD analysis results of sewage sludge ash catalyst before and after PS pyrolysis experiments.

.	Analyte	SSA, wt%	SSA After PS Pyrolysis 1:1, wt%	SSA After PS Pyrolysis 1:2, wt%	SSA After PS Pyrolysis 1:4, wt%
Quartz	SiO_2	25.4	27.1	33.1	30.8
Albite	$NaAlSi_3O_8$	23.4	21.1	21.3	27.5
Maghemite	Fe_2O_3	5.7			
Magnetite	Fe_3O_4		2.7	1.0	1.3
Gehlenite	$Ca_2Al_2SiO_7$	20.4		10.6	
Calcium Sulfate	$CaSO_4$	17.0	4.1	2.9	2.6
Calcium phosphate	$Ca_4P_2O_9$	8.0	18.0	7.8	7.8
K-oxide / peroxide	KO_2		12.1		11.8
Microcline	$(K,Na)AlSi_3O_8$		14.8	13.7	18.2
Sapphirine-2M	$Mg_{3.78}Al_{8.31}Si_{1.91}O_{20}$			9.7	

Table S3. XRD analysis results of sewage sludge char catalyst before and after PS pyrolysis experiments.

.	Analyte	SSC, wt%	SSC After PS Pyrolysis 1:1, wt%	SSC After PS Pyrolysis 1:2, wt%	SSC After PS Pyrolysis 1:4, wt%
Quartz	SiO_2	26.0	53.1	31.9	34.7
Albite	$NaAlSi_3O_8$	21.8	18.5	26.2	29.1
Maghemite	Fe_2O_3	6.2			
Gehlenite	$Ca_2Al_2SiO_7$	18.6	4.4	11.7	9.0
Calcium Sulfate	$CaSO_4$	14.7			
Calcium Phosphate	$Ca_4P_2O_9$	9.2	5.4	9.8	7.4
Calcium Oxide	CaO	3.5			
Microcline	$(K,Na)AlSi_3O_8$		15.2	15.6	15.1
Sapphirine-2M	$Mg_{3.78}Al_{8.31}Si_{1.91}O_{20}$		3.4	4.9	4.7

Table S4. XRD analysis results of acid-leached Fe-impregnated sewage sludge char catalyst before and after PS pyrolysis experiments.

.	Analyte	Fe-LSSC, wt%	Fe-LSSC After PS Pyrolysis 1:1, wt%	Fe-LSSC After PS Pyrolysis 1:2, wt%	Fe-LSSC After PS Pyrolysis 1:4, wt%
Quartz	SiO_2	58.7	43.6	42.6	45.8
Albite	$NaAlSi_3O_8$	19.0	17.5	20.2	20.8
Magnetite	Fe_3O_4		8.1	6.6	1.3
Microcline	$(K,Na)AlSi_3O_8$	22.3	22.1	20.6	20.6
Sapphirine-2M	$Mg_{3.78}Al_{8.31}Si_{1.91}O_{20}$		8.7	10.0	11.5

Table S5. GC-MS analysis result of oil produced during thermal pyrolysis of PS.

RT	Name	Area	ω, %	Formula	Category	Group	Oxygen content
4.5	Water	102323	0.2	H2O	Inorganic solvent	Other	0.0017762
12.3	Benzene	1739306	3.0	C6H6	Aromatic hydrocarbon	Light aromatic fraction	0

15.2	Toluene	982363	1.7	C7H8	Aromatic hydrocarbon	Light aromatic fraction	0
17.4	Ethylbenzene	645634	1.1	C8H10	Aromatic hydrocarbon	Light aromatic fraction	0
18.2	Styrene	20065828	34.5	C8H8	Alkene	Light aromatic fraction	0
18.8	Benzene, (1-methylethyl)-	42504	0.1	C9H12	Aromatic hydrocarbon	Light aromatic fraction	0
19.3	Tricyclo[3.2.1.0(2,,4)]oct-6-ene, 8-methylene-, (1.alpha.,2.alpha.,4.alpha.,5.alpha.)-	66363	0.1	C9H10	Polycyclic unsaturated hydrocarbon	Cyclic unsaturated compounds	0
19.3	Benzeneethanol, .beta.-ethenyl-	63136	0.1	C10H12	Aromatic alcohol	Oxygen-containing compounds	0
20.0	.alpha.-Methylstyrene	2130460	3.7	C9H10	Vinyl aromatic hydrocarbon	Light aromatic fraction	0
20.2	Succinic acid, di(3-phenylpropyl) ester	32617	0.1	C22H26O4	Aromatic ester	Oxygen-containing compounds	0.0001806
20.2	Benzaldehyde	379413	0.7	C7H6O	Aromatic aldehyde	Oxygen-containing compounds	0.0010553
20.3	Diglycolic acid, di(3-phenylpropyl) ester	49773	0.1	C22H26O5	Aromatic ester	Oxygen-containing compounds	0.0002159
20.4	Pimelic acid, di(3-phenylpropyl) ester	18699	0.0	C25H32O4	Aromatic ester	Oxygen-containing compounds	0
20.4	3-Phenyl-1-propanol, acetate	18989	0.0	C11H14O2	Aromatic ester	Oxygen-containing compounds	0
20.9	Benzene, cyclopropyl-	225701	0.4	C9H10	Alkylbenzene	Light aromatic fraction	0
20.9	Benzene, 2-propenyl-	223129	0.4	C9H10	Alkenylbenzene	Light aromatic fraction	0
21.2	Fumaric acid, di(3-phenylpropyl) ester	36028	0.1	C22H24O4	Aromatic ester	Oxygen-containing compounds	0.0001816
21.6	Benzene, 1-propynyl-	748165	1.3	C9H8	Alkynylbenzene	Light aromatic fraction	0
22.0	Oxirane, phenyl-	37640	0.1	C8H8O	Aromatic epoxide	Oxygen-containing compounds	0.0001332
22.3	Acetophenone	51618	0.1	C8H8O	Aromatic ketone	Oxygen-containing compounds	0.0001332
22.6	2-Methylindene	91770	0.2	C10H10	Alkyl-substituted indene	Cyclic unsaturated compounds	0
22.8	1H-Indene, 3-methyl-	48098	0.1	C10H10	Alkyl-substituted indene	Cyclic unsaturated compounds	0

23.4	Cycloprop[a]indene, 1,1a,6,6a-tetrahydro-	90444	0.2	C10H10	Fused cyclic unsaturated hydrocarbon	Cyclic unsaturated compounds	0
23.5	Benzene, 1-methyl-4-(1-propynyl)-	134468	0.2	C10H10	Alkynyl-substituted alkylbenzene	Light aromatic fraction	0
23.7	Naphthalene, 1,2-dihydro-	139632	0.2	C10H10	Partially hydrogenated PAH	PAHs	0
24.3	Naphthalene	3010668	5.2	C10H8	PAH	PAHs	0
25.0	Benzeneethanol, .beta.-methylene-	142959	0.2	C9H10O	Aromatic alcohol	Oxygen-containing compounds	0.0002385
25.7	Succinic acid, 2,4,6-trichlorophenyl 2-naphthylmethyl ester	58930	0.1	C21H13Cl3O4	Halogenated aromatic ester	Oxygen-containing compounds	0.0001469
26.0	Naphthalene, 2-methyl-	149143	0.3	C11H10	Alkyl-substituted PAH	PAHs	0
26.4	1H-Indene, 1-ethylidene-	131698	0.2	C11H10	Alkenyl-substituted indene	Cyclic unsaturated compounds	0
27.3	Biphenyl	3020705	5.2	C12H10	Biaryl hydrocarbon	Oligomers	0
28.1	Diphenylmethane	220748	0.4	C13H12	Diaryl alkane	Oligomers	0
28.5	Naphthalene, 2-ethenyl-	112158	0.2	C12H10	Alkenyl-substituted PAH	PAHs	0
29.2	1,1'-Biphenyl, 3-methyl-	247329	0.4	C13H12	Alkyl-substituted biaryl hydrocarbon	Oligomers	0
29.4	Acenaphthylene	182080	0.3	C12H8	PAH	PAHs	0
29.6	cis-Stilbene	345350	0.6	C14H12	Diaryl alkene	Oligomers	0
29.9	(E)-Stilbene	188470	0.3	C14H12	Diaryl alkene	Oligomers	0
30.0	Bibenzyl	624768	1.1	C14H14	Diaryl alkane	Oligomers	0
30.6	Benzene, 1,1'-(1,2-dimethyl-1,2-ethanediyl)bis-, (R*,S*)-	402651	0.7	C16H18	Diaryl alkane	Oligomers	0
30.6	Benzene, 1,1'-(1-methyl-1,2-ethanediyl)bis-	418127	0.7	C15H16	Diaryl alkane	Oligomers	0
31.3	1H-Indene, 1-(phenylmethylene)-	557829	1.0	C16H12	Diaryl-substituted indene	Cyclic unsaturated compounds	0
32.1	1,1'-Biphenyl, 4-ethenyl-	412613	0.7	C14H12	Alkenylbiphenyl	Oligomers	0
32.4	Fluorene	780351	1.3	C13H10	PAH	PAHs	0
32.9	Phenanthrene, 9,10-dihydro-	227146	0.4	C14H12	Partially hydrogenated PAH	PAHs	0
33.2	Benzene, 1,1'-(1,3-propanediyl)bis-	3734685	6.4	C15H16	Diaryl alkane	Oligomers	0

34.0	Benzene, 1,1'-(1-methyl-1,3-propanediyl)bis-	200538	0.3	C16H18	Diaryl alkane	Oligomers	0
35.3	Naphthalene, 1,2,3,4-tetrahydro-2-phenyl-	10628471	18.3	C16H16	Partially hydrogenated PAH	PAHs	0
35.6	Anthracene, 9,10-dihydro-	2850976	4.9	C14H12	Partially hydrogenated PAH	PAHs	0
35.8	1,2-Diphenylcyclopropane	661632	1.1	C15H14	Diaryl cycloalkane	Oligomers	0
36.4	Ethanol, 2-[bis(phenylmethyl)amino]-	76106	0.1	C16H19 NO	Aromatic amine	Oxygen-containing compounds	0.0000663
37.8	1H-Indene, 2-phenyl-	176562	0.3	C15H12	Aryl-substituted indene	Cyclic unsaturated compounds	0
39.4	Benzene, 1,1'-(1-butene-1,4-diyl)bis-, (Z)-	406743	0.7	C16H16	Diaryl alkene	Oligomers	0
		58133533					0.0041277

Table S6. GC-MS analysis result of oil produced during catalytic pyrolysis of sewage sludge ash with PS with rate 1:1.

RT	Name	Area	ω, %	Formula	Category	Group	Oxygen content
3.50	Nitrogen	98938.2	0.19	N2	Inorganic gas	Other	0
3.51	Oxygen	42265.5	0.08	O2	Inorganic gas	Other	0.0008000
4.51	Water	1377009	2.71	H2O	Inorganic solvent	Other	0.0240673
10.03	Formamide	37831.3	0.07	CH3NO	Amide	Oxygen-containing compounds	0.0002486
12.36	Benzene	10016713	19.70	C6H6	Aromatic hydrocarbon	Light aromatic fraction	0
15.26	Toluene	2141672	4.21	C7H8	Alkylbenzene	Light aromatic fraction	0
17.51	Benzene, 1,3-dimethyl-	496880.5	0.98	C8H10	Alkylbenzene	Light aromatic fraction	0
18.30	Styrene	17183104	33.80	C8H8	Vinyl aromatic hydrocarbon	Light aromatic fraction	0
19.34	Benzeneethanol, .beta.-ethenyl-	53358.7	0.10	C10H12	Aromatic alcohol	Oxygen-containing compounds	0
20.05	.alpha.-Methylstyrene	2308684	4.54	C9H10	Vinyl aromatic hydrocarbon	Light aromatic fraction	0

20.26	3-Phenyl-1-propanol, acetate	26323.3	0.05	C11H14O2	Aromatic ester	Oxygen-containing compounds	0.0000898
20.31	Benzaldehyde	890169	1.75	C7H6O	Aromatic aldehyde	Oxygen-containing compounds	0.0026383
20.39	Diglycolic acid, di(3-phenylpropyl) ester	31124.7	0.06	C22H26O5	Aromatic ester	Oxygen-containing compounds	0.0001296
20.47	Acetic acid, chloro-, 3-phenylpropyl ester	9740.3	0.02	C11H13ClO2	Halogenated aromatic ester	Oxygen-containing compounds	0.0000301
21.01	Benzene, cyclopropyl-	158956.1	0.31	C9H10	Alkylbenzene	Cyclic unsaturated compounds	0.0000000
21.29	2-Vinylfuran	4780.8	0.01	C6H6O	Oxygenated aromatic heterocycle	Oxygen-containing compounds	0.0000170
21.65	Indene	629302.9	1.24	C9H8	Indene	Cyclic unsaturated compounds	0
21.88	Benzeneacetaldehyde	127908	0.25	C8H8O	Aromatic aldehyde	Oxygen-containing compounds	0.0003329
22.32	Acetophenone	122667	0.24	C8H8O	Aromatic ketone	Oxygen-containing compounds	0.0003196
22.51	1-Methyl-2-phenylcyclopropane	16565.3	0.03	C10H12	Alkyl-substituted cycloalkane	Cyclic unsaturated compounds	0
22.65	Naphthalene, 1,2-dihydro-	26590	0.05	C10H10	Partially hydrogenated PAH	Cyclic unsaturated compounds	0
23.47	1H-Indene, 3-methyl-	59337.4	0.12	C10H10	Alkyl-substituted indene	Cyclic unsaturated compounds	0
23.63	2-Methylindene	50607.8	0.10	C10H10	Alkyl-substituted indene	Cyclic unsaturated compounds	0
23.84	1,4-Dihydronaphthalene	184239.2	0.36	C10H10	Partially hydrogenated PAH	Cyclic unsaturated compounds	0
24.38	Naphthalene	2995828	5.89	C10H8	PAH	PAHs	0

26.15	Naphthalene, 2-methyl-	146829.9	0.29	C11H10	Alkyl-substituted PAH	PAHs	0
26.49	1H-Indene, 1-ethylidene-	127186.6	0.25	C11H10	Alkenyl-substituted indene	Cyclic unsaturated compounds	0
27.40	Biphenyl	3435889	6.76	C12H10	Biaryl hydrocarbon	Oligomers	0
28.24	Diphenylmethane	158395	0.31	C13H12	Diaryl alkane	Oligomers	0
28.63	Naphthalene, 2-ethenyl-	66730.4	0.13	C12H10	Alkenyl-substituted PAH	PAHs	0
29.33	1,1'-Biphenyl, 3-methyl-	202244	0.40	C13H12	Alkyl-substituted biaryl hydrocarbon	Oligomers	0
29.51	Acenaphthylene	153809.6	0.30	C12H8	PAH	PAHs	0
29.78	(E)-Stilbene	157635.4	0.31	C14H12	Diaryl alkene	Oligomers	0
30.08	cis-Stilbene	98732	0.19	C14H12	Diaryl alkene	Oligomers	0
30.20	Benzyl sulfone	665788.2	1.31	C14H14 O2S	Sulfur-containing aromatic compound	Oxygen-containing compounds	0.0017017
30.82	Benzene, 1,1'-(1-methyl-1,2-ethanediyl)bis-	383310.9	0.75	C15H16	Diaryl alkane	Oligomers	0.0000000
31.69	1-Octanol, 2-butyl-	486903.7	0.96	C12H26 O	Aliphatic alcohol	Oxygen-containing compounds	0.0008243
32.34	1,1'-Biphenyl, 4-ethenyl-	190873.3	0.38	C14H12	Alkenylbiphen yl	Oligomers	0
32.62	Fluorene	554789	1.09	C13H10	PAH	PAHs	0
33.10	Phenanthrene, 9,10-dihydro-	77217	0.15	C14H10	Partially hydrogenated PAH	PAHs	0
33.41	Benzene, 1,1'-(1,3-propanediyl)bis-	2123231	4.18	C15H16	Diaryl alkane	Oligomers	0
34.82	3,4-Hexanedione, 2,2,5-trimethyl-	28374.5	0.06	C9H16O 2	Aliphatic ketone	Oxygen-containing compounds	0.0001229
35.86	Anthracene, 9,10-dihydro-	1777623	3.50	C14H12	Partially hydrogenated PAH	PAHs	0
36.04	1,2-Diphenylcyclopropane	636930.7	1.25	C15H14	Diaryl cycloalkane	Oligomers	0

36.69	Ethanol, 2-[bis(phenylmethyl)amino]-	49015	0.10	C16H19NO	Aromatic amine	Other	0.0000663
39.66	Benzene, 1,1'-(1-butene-1,4-diyl)bis-, (Z)-	232384	0.46	C16H16	Diaryl alkene	Oligomers	0
		50844487					0.0313884

Table S7. GC-MS analysis result of oil produced during catalytic pyrolysis of sewage sludge ash with PS with rate 1:2.

RT	Name	Area	ω, %	Formula	Category	Group	Oxygen content
4.5	Water	232169	0.4	H2O	Inorganic solvent	Other	0.0035524
12.3	Benzene	1728280	2.8	C6H6	Aromatic hydrocarbon	Light aromatic fraction	0
15.2	Toluene	1502094	2.4	C7H8	Alkylbenzene	Light aromatic fraction	0
17.4	Ethylbenzene	942067	1.5	C8H10	Alkylbenzene	Light aromatic fraction	0
18.2	Styrene	24920740	40.5	C8H8	Vinyl aromatic hydrocarbon	Light aromatic fraction	0
19.3	Indane	63968	0.1	C9H10	Indane	Cyclic unsaturated compounds	0
19.4	Benzene, propyl-	19640	0.0	C9H12	Alkylbenzene	Light aromatic fraction	0
20.0	.alpha.-Methylstyrene	2609998	4.2	C9H10	Vinyl aromatic hydrocarbon	Light aromatic fraction	0
20.2	Benzeneethanol, .beta.-ethenyl-	16213	0.0	C10H12O	Aromatic alcohol	Oxygen-containing compounds	0
20.2	Benzaldehyde	238568	0.4	C7H6O	Aromatic aldehyde	Oxygen-containing compounds	0.0006030
20.9	Benzene, cyclopropyl-	179243	0.3	C9H10	Alkylbenzene	Cyclic unsaturated compounds	0
21.6	Indene	572737	0.9	C9H8	Indene	Cyclic unsaturated compounds	0
22.3	Acetophenone	70984	0.1	C8H8O	Aromatic ketone	Oxygen-containing compounds	0.0001332
22.6	Naphthalene, 1,2-dihydro-	42694	0.1	C10H10	Partially hydrogenated PAH	Cyclic unsaturated compounds	0

22.8	Naphthalene, 1-phenyl-	357651	0.6	C16H12	Aryl-substituted PAH	PAHs	0
23.4	2-Methylindene	58902	0.1	C10H10	Alkyl-substituted indene	Cyclic unsaturated compounds	0
23.5	1H-Indene, 3-methyl-	99323	0.2	C10H10	Alkyl-substituted indene	Cyclic unsaturated compounds	0
23.7	Cycloprop[a]indene, 1,1a,6,6a-tetrahydro-	107343	0.2	C10H10	Fused cyclic unsaturated hydrocarbon	Cyclic unsaturated compounds	0
24.3	Naphthalene	3103492	5.0	C10H8	PAH	PAHs	0
25.7	Naphthalene, 2-methyl-	77185	0.1	C11H10	Alkyl-substituted PAH	PAHs	0
26.0	1H-Indene, 1-ethylidene-	178485	0.3	C11H10	Alkenyl-substituted indene	Cyclic unsaturated compounds	0
26.4	Naphthalene, 1-methyl-	145667	0.2	C11H10	Alkyl-substituted PAH	PAHs	0
27.3	Biphenyl	3797427	6.2	C12H10	Biaryl hydrocarbon	Oligomers	0
28.1	1,1'-Biphenyl, 2-methyl-	194155	0.3	C13H12	Alkyl-substituted biaryl hydrocarbon	Oligomers	0
28.5	Naphthalene, 2-ethenyl-	144759	0.2	C12H10	Alkenyl-substituted PAH	PAHs	0
28.5	Acenaphthene	143711	0.2	C12H10	PAH	PAHs	0
29.2	1,1'-Biphenyl, 3-methyl-	235536	0.4	C13H12	Alkyl-substituted biaryl hydrocarbon	Oligomers	0
29.3	Acenaphthylene	255020	0.4	C12H8	PAH	PAHs	0
29.6	(E)-Stilbene	274478	0.4	C14H12	Diaryl alkene	Oligomers	0
29.9	cis-Stilbene	194887	0.3	C14H12	Diaryl alkene	Oligomers	0
30.0	Bibenzyl	514769	0.8	C14H14	Diaryl alkane	Oligomers	0
30.6	Benzene, 1,1'-(1,2-dimethyl-1,2-ethanediyl)bis-, (R*,S*)-	304848	0.5	C16H18	Diaryl alkane	Oligomers	0

31.3	1H-Indene, 1-(phenylmethylene)-	925659	1.5	C16H12	Diaryl-substituted indene	Cyclic unsaturated compounds	0
32.1	1,1'-Biphenyl, 4-ethenyl-	355788	0.6	C14H12	Alkenylbiphenyl	Oligomers	0
32.4	Fluorene	706328	1.1	C13H10	PAH	PAHs	0
32.9	Stilbene	210247	0.3	C14H12	Diaryl alkene	Oligomers	0
33.2	Benzene, 1,1'-(1,3-propanediyl)bis-	3233463	5.3	C15H16	Diaryl alkane	Oligomers	0
34.5	Dodecyl heptyl ether	267673	0.4	C19H40O	Aliphatic ether	Oxygen-containing compounds	0.0002249
35.3	Naphthalene, 1,2,3,4-tetrahydro-2-phenyl-	9179789	14.9	C16H16	Partially hydrogenated PAH	PAHs	0
35.6	Anthracene, 9,10-dihydro-	1986668	3.2	C14H12	Partially hydrogenated PAH	PAHs	0
35.8	1,2-Diphenylcyclopropane	668475	1.1	C15H14	Diaryl cycloalkane	Oligomers	0
36.4	Ethanol, 2-[bis(phenylmethyl)amino]-	69434	0.1	C16H19NO	Aromatic amine	Other	0.0000663
37.8	1H-Indene, 1-phenyl-	169997	0.3	C15H12	Aryl-substituted indene	Cyclic unsaturated compounds	0
39.3	Benzene, 1,1'-(1-butene-1,4-diyl)bis-, (Z)-	369673	0.6	C16H16	Diaryl alkene	Oligomers	0
		61470226					0.0045798

Table S8. GC-MS analysis result of oil produced during catalytic pyrolysis of sewage sludge ash with PS with rate 1:4.

RT	Name	Area	ω, %	Formula	Category	Group	Oxygen content
4.5	Water	121998.2	0.2	H2O	Inorganic solvent	Other	0.0017762
12.3	Benzene	1000310.1	2.0	C6H6	Aromatic hydrocarbon	Light aromatic fraction	0
15.2	Toluene	1200827.4	2.4	C7H8	Alkylbenzene	Light aromatic fraction	0
17.4	Ethylbenzene	827158.3	1.6	C8H10	Alkylbenzene	Light aromatic fraction	0
18.2	Styrene	24267477.5	47.5	C8H8	Vinyl aromatic hydrocarbon	Light aromatic fraction	0
19.3	Indane	73430.5	0.1	C9H10	Indane	Cyclic unsaturated compounds	0

19.3	Benzene, 2-propenyl-	75045.1	0.1	C9H10	Alkenylbenzene	Light aromatic fraction	0
20.0	.alpha.-Methylstyrene	2257574.7	4.4	C9H10	Vinyl aromatic hydrocarbon	Light aromatic fraction	0
20.2	Pimelic acid, di(3-phenylpropyl) ester	31299.4	0.1	C20H24O4	Aromatic ester	Oxygen-containing compounds	0.0001949
20.2	Benzaldehyde	383237	0.8	C7H6O	Aromatic aldehyde	Oxygen-containing compounds	0.0012061
20.3	Diglycolic acid, di(3-phenylpropyl) ester	41235.2	0.1	C22H26O5	Aromatic ester	Oxygen-containing compounds	0.0002159
20.4	Benzene, 1,1'-(1-ethenyl-1,3-propanediyl)bis-	14507	0.0	C17H18	Diaryl alkene	Oligomers	0
20.9	Benzene, cyclopropyl-	217346.6	0.4	C9H10	Alkylbenzene	Cyclic unsaturated compounds	0
21.6	Indene	555468.2	1.1	C9H8	Indene	Cyclic unsaturated compounds	0
21.8	Benzeneacetaldehyde	137179.4	0.3	C8H8O	Aromatic aldehyde	Oxygen-containing compounds	0.0003995
22.0	Oxirane, phenyl-	47023.9	0.1	C8H8O	Aromatic epoxide	Oxygen-containing compounds	0.0001332
22.2	Acetophenone	48200.9	0.1	C8H8O	Aromatic ketone	Oxygen-containing compounds	0.0001332
22.6	Cycloprop[a]indene, 1,1a,6,6a-tetrahydro-	49639	0.1	C10H10	Fused cyclic unsaturated hydrocarbon	Cyclic unsaturated compounds	0
22.8	Naphthalene, 1-phenyl-	209142.1	0.4	C16H12	Aryl-substituted PAH	PAHs	0
23.4	1H-Indene, 3-methyl-	66499.7	0.1	C10H10	Alkyl-substituted indene	Cyclic unsaturated compounds	0
23.5	Benzene, 1-methyl-4-(1-propynyl)-	77802.7	0.2	C10H10	Alkynyl-substituted alkylbenzene	Light aromatic fraction	0

23.7	Naphthalene, 1,2-dihydro-	97801.4	0.2	C10H10	Partially hydrogenated PAH	Cyclic unsaturated compounds	0
24.3	Naphthalene	2168884.9	4.2	C10H8	PAH	PAHs	0
25.0	Benzeneethanol, .beta.-methylene-	101078	0.2	C10H10O	Aromatic alcohol	Oxygen-containing compounds	0.0002189
26.0	1H-Indene, 1-ethylidene-	110209.2	0.2	C11H10	Alkenyl-substituted indene	Cyclic unsaturated compounds	0
26.4	Naphthalene, 2-methyl-	100746	0.2	C11H10	Alkyl-substituted PAH	PAHs	0
27.3	Biphenyl	1829994.4	3.6	C12H10	Biaryl hydrocarbon	Oligomers	0
28.1	Diphenylmethane	129998.2	0.3	C13H12	Diaryl alkane	Oligomers	0
28.5	Acenaphthene	67057.6	0.1	C12H10	PAH	PAHs	0
29.2	1,1'-Biphenyl, 4-methyl-	129931.8	0.3	C13H12	Alkyl-substituted biaryl hydrocarbon	Oligomers	0
29.3	Acenaphthylene	130772.4	0.3	C12H8	PAH	PAHs	0
29.6	(E)-Stilbene	168778.7	0.3	C14H12	Diaryl alkene	Oligomers	0
29.9	cis-Stilbene	78463.1	0.2	C14H12	Diaryl alkene	Oligomers	0
30.0	Bibenzyl	416757.2	0.8	C14H14	Diaryl alkane	Oligomers	0
30.6	Benzene, 1,1'-(1-methyl-1,2-ethanediyl)bis-	261223.4	0.5	C15H16	Diaryl alkane	Oligomers	0
30.6	Benzene, 1,1'-(1,2-dimethyl-1,2-ethanediyl)bis-, (R*,S*)-	264389.3	0.5	C16H18	Diaryl alkane	Oligomers	0
31.3	Acephenanthrylene, 4,5-dihydro-	548934.5	1.1	C16H12	Acephenanthrylene derivative	PAHs	0
32.1	1,1'-Biphenyl, 4-ethenyl-	185481	0.4	C14H12	Alkenylbiphenyl	Oligomers	0
32.4	Fluorene	362447.3	0.7	C13H10	PAH	PAHs	0
32.9	Benzene, 1,1',1'',1'''-(1,2,3,4-cyclobutanetetrayl)tetrakis-	91850.3	0.2	C28H28	Polyaryl hydrocarbon	Oligomers	0
33.2	Benzene, 1,1'-(1,3-propanediyl)bis-	2279111.1	4.5	C15H16	Diaryl alkane	Oligomers	0
35.3	Naphthalene, 1,2,3,4-tetrahydro-2-phenyl-	7744668	15.2	C16H16	Partially hydrogenated PAH	PAHs	0
35.6	Anthracene, 9,10-dihydro-	1322364.2	2.6	C14H12	Partially hydrogenated PAH	PAHs	0

35.8	1,2-Diphenylcyclopropane	452480.3	0.9	C15H14	Diaryl cycloalkane	Oligomers	0
39.3	Benzene, 1,1'-(1-butene-1,4-diyl)bis-, (Z)-	309788.6	0.6	C16H16	Diaryl alkene	Oligomers	0
		51055613.8					0.0042779

Table S9. GC-MS analysis result of oil produced during catalytic pyrolysis of sewage sludge ash with PS with rate 1:10.

RT	Name	Area	ω, %	Formula	Category	Group	Oxygen content
4.5	Water	238932	0.42	H2O	Inorganic solvent	Other	0.0037300
12.3	Benzene	792100	1.39	C6H6	Aromatic hydrocarbon	Light aromatic fraction	0
15.2	Toluene	1074603	1.88	C7H8	Alkylbenzene	Light aromatic fraction	0
17.4	Ethylbenzene	880060	1.54	C8H10	Alkylbenzene	Light aromatic fraction	0
18.2	Styrene	27004427	47.23	C8H8	Vinyl aromatic hydrocarbon	Light aromatic fraction	0
18.8	Benzene, (1-methylethyl)-	48386	0.08	C9H12	Alkylbenzene	Light aromatic fraction	0
19.3	Benzene, 2-propenyl-	84915	0.15	C9H10	Alkenylbenzene	Light aromatic fraction	0
19.3	Indane	78732	0.14	C9H10	Indane	Cyclic unsaturated compounds	0
20.0	.alpha.-Methylstyrene	2454203	4.29	C9H10	Vinyl aromatic hydrocarbon	Light aromatic fraction	0
20.2	Diglycolic acid, di(3-phenylpropyl) ester	31811	0.06	C22H26O5	Aromatic ester	Oxygen-containing compounds	0.0001296
20.2	Pimelic acid, di(3-phenylpropyl) ester	32882	0.06	C20H24O4	Aromatic ester	Oxygen-containing compounds	0.0001169
20.2	Benzaldehyde	353806	0.62	C7H6O	Aromatic aldehyde	Oxygen-containing compounds	0.0009347
20.3	Succinic acid, di(3-phenylpropyl) ester	37756	0.07	C20H22O4	Aromatic ester	Oxygen-containing compounds	0.0001372
20.4	Benzeneethanol, .beta.-ethenyl-	15889	0.03	C10H12O	Aromatic alcohol	Oxygen-containing compounds	0.0000324

20.9	Benzene, cyclopropyl-	238593	0.42	C9H10	Alkylbenzene	Cyclic unsaturated compounds	0
20.9	Benzene, 1-ethenyl-2-methyl-	244988	0.43	C10H10	Alkenyl-substituted alkylbenzene	Light aromatic fraction	0
21.2	Fumaric acid, di(3-phenylpropyl) ester	28953	0.05	C20H22O4	Aromatic ester	Oxygen-containing compounds	0.0000980
21.2	3-Phenyl-1-propanol, acetate	29632	0.05	C11H14O2	Aromatic ester	Oxygen-containing compounds	0.0000898
21.6	Indene	614229	1.07	C9H8	Indene	Cyclic unsaturated compounds	0
21.8	Benzeneacetaldehyde	130407	0.23	C8H8O	Aromatic aldehyde	Oxygen-containing compounds	0.0003063
22.0	Oxirane, phenyl-	49601	0.09	C8H8O	Aromatic epoxide	Oxygen-containing compounds	0.0001198
22.3	Acetophenone	40598	0.07	C8H8O	Aromatic ketone	Oxygen-containing compounds	0.0000932
22.6	Benzene, 1,4-diethenyl-	60339	0.11	C10H10	Alkenyl-substituted benzene	Light aromatic fraction	0
22.8	1-Methyl-1-phenylcyclobutane	20934	0.04	C11H14	Alkyl-substituted cycloalkane	Cyclic unsaturated compounds	0
23.4	Benzene, 1-methyl-4-(1-propynyl)-	71108	0.12	C10H10	Alkynyl-substituted alkylbenzene	Light aromatic fraction	0
23.5	2-Methylindene	65876	0.12	C10H10	Alkyl-substituted indene	Cyclic unsaturated compounds	0
23.7	Naphthalene, 1,2-dihydro-	109747	0.19	C10H10	Partially hydrogenated PAH	Cyclic unsaturated compounds	0
24.3	Naphthalene	2133489	3.73	C10H8	PAH	PAHs	0
24.7	Cyclooctasiloxane, hexadecamethyl-	507619	0.89	C16H48O8Si8	Siloxane	Other	0.0019202

25.0	Benzeneethanol, .beta.-methylene-	99395	0.17	C10H10O	Aromatic alcohol	Oxygen-containing compounds	0.0001860
26.0	1H-Indene, 1-ethylidene-	87277	0.15	C11H10	Alkenyl-substituted indene	Cyclic unsaturated compounds	0
26.4	Naphthalene, 2-methyl-	84688	0.15	C11H10	Alkyl-substituted PAH	PAHs	0
27.3	Biphenyl	2004507	3.51	C12H10	Biaryl hydrocarbon	Oligomers	0
28.1	Diphenylmethane	141619	0.25	C13H12	Diaryl alkane	Oligomers	0
28.5	Naphthalene, 2-ethenyl-	77003	0.13	C12H10	Alkenyl-substituted PAH	PAHs	0
29.2	1,1'-Biphenyl, 4-methyl-	150782	0.26	C13H12	Alkyl-substituted biaryl hydrocarbon	Oligomers	0
29.4	Acenaphthylene	136551	0.24	C12H8	PAH	PAHs	0
29.6	Ethylene, 1,1-diphenyl-	208433	0.36	C14H12	Diaryl-substituted alkene	Oligomers	0
29.9	Anthracene, 9,10-dihydro-9-(1-methylpropyl)-	80881	0.14	C18H20	Partially hydrogenated PAH	PAHs	0
30.0	Bibenzyl	581860	1.02	C14H14	Diaryl alkane	Oligomers	0
30.7	Benzene, 1,1'-(1-methyl-1,2-ethanediyl)bis-	323173	0.57	C15H16	Alkenyl-substituted biaryl hydrocarbon	Oligomers	0
32.1	1,1'-Biphenyl, 4-ethenyl-	242210	0.42	C14H12	Alkenylbiphenyl	Oligomers	0
32.4	Fluorene	496168	0.87	C13H10	Diaryl alkene	PAHs	0
32.9	(E)-Stilbene	124918	0.22	C14H12	Diaryl alkane	Oligomers	0
33.2	Benzene, 1,1'-(1,3-propanediyl)bis-	2691858	4.71	C15H16	Diaryl alkane	Oligomers	0
34.0	Benzene, 1,1'-(1-methyl-1,3-propanediyl)bis-	114852	0.20	C16H18	Partially hydrogenated PAH	Oligomers	0
35.3	Naphthalene, 1,2,3,4-tetrahydro-2-phenyl-	9544018	16.69	C16H16	Partially hydrogenated PAH	PAHs	0

35.6	Anthracene, 9,10-dihydro-	1652368	2.89	C14H12	Diaryl cycloalkane	PAHs	0
35.8	1,2-Diphenylcyclopropane	534703	0.94	C15H14	Diaryl alkene	Oligomers	0
39.4	Benzene, 1,1'-(1-butene-1,4-diyl)bis-, (Z)-	325937	0.57	C16H16	Aromatic hydrocarbon	Oligomers	0
		57177812					0.0078941

Table S10. GC-MS analysis result of oil produced during catalytic pyrolysis of sewage sludge char with PS with rate 1:1.

RT	Name	Area	ω, %	Formula	Category	Group	Oxygen content
3.62	Ethylene	7566	0.01	C2H4	Alkene	Other	0
3.95	Propene	8936.4	0.02	C3H6	Alkene	Other	0
4.51	Water	771457.8	1.39	H2O	Inorganic solvent	Other	0.0123445
10.03	4-Penten-2-ol	63475	0.11	C5H10O	Aliphatic alcohol	Oxygen-containing compounds	0.0002043
12.36	Benzene	9213227.1	16.57	C6H6	Aromatic hydrocarbon	Light aromatic fraction	0
15.26	Toluene	1243383.6	2.24	C7H8	Alkylbenzene	Light aromatic fraction	0
17.26	Phenanthrene	3223518.4	5.80	C14H10	PAH	PAHs	0
17.26	9H-Fluorene, 9-methylene-	3378902.3	6.08	C14H10	PAH	PAHs	0
17.51	Ethylbenzene	549730	0.99	C8H10	Alkylbenzene	Light aromatic fraction	0
18.29	Styrene	14598313	26.25	C8H8	Vinyl aromatic hydrocarbon	Light aromatic fraction	0
19.34	Benzeneethanol, .beta.-ethenyl-	38850.5	0.07	C10H12O	Aromatic alcohol	Oxygen-containing compounds	0.0000756
20.05	.alpha.-Methylstyrene	2226182.7	4.00	C9H10	Alkenylbenzene	Light aromatic fraction	0
20.25	3-Phenyl-1-propanol, acetate	25379.8	0.05	C11H14O2	Aromatic ester	Oxygen-containing compounds	0.0000898
20.31	Benzaldehyde	386792.1	0.70	C7H6O	Aromatic aldehyde	Oxygen-containing compounds	0.0010553
20.39	Diglycolic acid, di(3-phenylpropyl) ester	30535.9	0.05	C22H26O5	Diester	Oxygen-containing compounds	0.0001080

21.00	Indane	168252.6	0.30	C9H10	Indane	Cyclic unsaturated compounds	0
21.64	Indene	616142.9	1.11	C9H8	Indene	Cyclic unsaturated compounds	0
21.88	Benzeneacetaldehyde	26194.8	0.05	C8H8O	Aromatic aldehyde	Oxygen-containing compounds	0.0000666
22.32	Acetophenone	77687.6	0.14	C8H8O	Aromatic ketone	Oxygen-containing compounds	0.0001864
22.65	Naphthalene, 1,2-dihydro-	52094.8	0.09	C10H10	Partially hydrogenated PAH	Cyclic unsaturated compounds	0
23.33	1H-Indene, 1-(phenylmethylene)-	1082794.1	1.95	C16H12	Diaryl-substituted indene	Cyclic unsaturated compounds	0
23.47	1H-Indene, 3-methyl-	65083.2	0.12	C10H10	Alkyl-substituted indene	Cyclic unsaturated compounds	0
23.62	2-Methylindene	56670.6	0.10	C10H10	Alkyl-substituted indene	Cyclic unsaturated compounds	0
24.12	Naphthacene, 5,12-dihydro-	21776.8	0.04	C18H14	Partially hydrogenated PAH	PAHs	0
24.38	Naphthalene	2701073.4	4.86	C10H8	PAH	PAHs	0
24.67	1H-Indene, 2-phenyl-	951025.1	1.71	C15H12	Aryl-substituted indene	Cyclic unsaturated compounds	0
25.92	3,3-Diphenyl-5-methyl-3H-pyrazole	174914.3	0.31	C16H16 N2	Heterocyclic compound	Other compounds	0
26.14	Naphthalene, 2-methyl-	136632.1	0.25	C11H10	PAH	PAHs	0
26.48	1H-Indene, 1-ethylidene-	126534.2	0.23	C11H10	Alkenyl-substituted indene	Cyclic unsaturated compounds	0
27.39	Biphenyl	3266362.2	5.87	C12H10	Biphenyl	Oligomers	0
28.23	Diphenylmethane	150158.5	0.27	C13H12	Diarylmethane	Oligomers	0
28.64	Naphthalene, 2-ethenyl-	65559.4	0.12	C12H10	Alkenylnaphth alene	PAHs	0

28.64	Acenaphthene	64911	0.12	C12H10	Partially hydrogenated PAH	PAHs	0
29.32	1,1'-Biphenyl, 3-methyl-	244193.4	0.44	C13H12	Alkylbiphenyl	Oligomers	0
29.51	Acenaphthylene	140386.3	0.25	C12H8	PAH	PAHs	0
29.78	Ethylene, 1,1-diphenyl-	213591	0.38	C14H12	Diarylalkene	Oligomers	0
30.08	(E)-Stilbene	113811.3	0.20	C14H12	Diarylalkene	Oligomers	0
30.08	Anthracene, 9,10-dihydro-	118598.6	0.21	C14H12	Partially hydrogenated PAH	PAHs	0
30.20	Bibenzyl	684927.4	1.23	C14H14	Diarylalkane	Oligomers	0
30.81	Benzene, 1,1'-(1,2-dimethyl-1,2-ethanediyl)bis-, (R*,S*)-	382593.8	0.69	C16H18	Diarylalkane	Oligomers	0
31.80	Naphthalene, 2-phenyl-	2829659.1	5.09	C16H12	PAH	PAHs	0
32.33	1,1'-Biphenyl, 4-ethenyl-	208289.8	0.37	C14H12	Alkenylbiphenyl	Oligomers	0
32.61	Fluorene	604512.9	1.09	C13H10	PAH	PAHs	0
33.10	Phenanthrene, 9,10-dihydro-	111622.1	0.20	C14H12	Partially hydrogenated PAH	PAHs	0
33.41	Benzene, 1,1'-(1,3-propanediyl)bis-	1515499.5	2.72	C15H16	Diarylalkane	Oligomers	0
35.86	Stilbene	2052044	3.69	C14H12	Diarylalkene	Oligomers	0
36.04	1,2-Diphenylcyclopropane	548901.4	0.99	C15H14	Diarylcycloalkane	Oligomers	0
38.08	1H-Indene, 1-phenyl-	122842.5	0.22	C15H12	Fused aromatic hydrocarbon	Cyclic unsaturated compounds	0
39.66	Benzene, 1,1'-(1-butene-1,4-diyl)bis-, (Z)-	156282.8	0.28	C16H16	Diarylalkene	Oligomers	0
		55617874.1					0.0141305

Table S11. GC-MS analysis result of oil produced during catalytic pyrolysis of sewage sludge char with PS with rate 1:2.

RT	Name	Area	ω, %	Formula	Category	Group	Oxygen content
4.5	Water	329372.6	2.4	H2O	Inorganic solvent	Other	0.0213142
12.3	Benzene	652078.2	4.7	C6H6	Aromatic hydrocarbon	Light aromatic fraction	0
15.2	Toluene	298542.9	2.1	C7H8	Alkylbenzene	Light aromatic fraction	0
17.4	Ethylbenzene	124319.1	0.9	C8H10	Alkylbenzene	Light aromatic fraction	0

18.2	Styrene	5071883.7	36.2	C8H8	Vinyl aromatic hydrocarbon	Light aromatic fraction	0
20.0	.alpha.-Methylstyrene	537355.3	3.8	C9H10	Vinyl aromatic hydrocarbon	Light aromatic fraction	0
20.3	1,3-Benzenediol, monobenzoate	31369.3	0.2	C13H10 O3	Aromatic ester	Oxygen-containing compounds	0.0004481
21.6	Benzene, 1-ethynyl-4-methyl-	125513.9	0.9	C9H8	Alkynylalkylbe nzene	Light aromatic fraction	0
22.6	2-Methylindene	10730.2	0.1	C10H10	Alkyl-substituted indene	Cyclic unsaturated compounds	0
22.8	3,10B-Dihydrofluoranthene	237884.6	1.7	C16H12	Partially hydrogenated PAH	PAHs	0
23.7	Naphthalene, 1,2-dihydro-	22464.7	0.2	C10H10	Partially hydrogenated PAH	Cyclic unsaturated compounds	0
24.3	Naphthalene	664353.9	4.7	C10H8	PAH	PAHs	0
26.0	Succinic acid, 2,4,6-trichlorophenyl 2-naphthylmethyl ester	27644.7	0.2	C21H15C l3O4	Chlorinated aromatic ester	Oxygen-containing compounds	0.0002924
27.3	Biphenyl	778449.9	5.6	C12H10	Biphenyl	Oligomers	0
28.1	Succinic acid, 3-methylbut-2-en-1-yl diphenylmethyl ester	32998.4	0.2	C22H22 O4	Ester	Oxygen-containing compounds	0.0003653
29.6	(E)-Stilbene	67362.2	0.5	C14H12	Diarylalkene	Oligomers	0
29.9	cis-Stilbene	38427.2	0.3	C14H12	Diarylalkene	Oligomers	0
30.0	Bibenzyl	101867.7	0.7	C14H14	Diarylalkane	Oligomers	0
30.6	Benzene, 1,1'-(1,2-dimethyl-1,2-ethanediyl)bis-, (R*,S*)-	63314.9	0.5	C16H18	Diarylalkane	Oligomers	0
31.3	Acephenanthrylene, 4,5-dihydro-	641233.6	4.6	C16H12	Partially hydrogenated PAH	PAHs	0
32.1	1,1'-Biphenyl, 4-ethenyl-	75244.1	0.5	C14H12	Alkenylbiphen yl	Oligomers	0
32.4	Fluorene	147358.8	1.1	C13H10	PAH	PAHs	0
32.9	Benzene, 1,1',1'',1'''-(1,2,3,4-cyclobutanetetrayl)tetrakis-	37678	0.3	C28H28	Polyaryl hydrocarbon	Oligomers	0
33.2	Benzene, 1,1'-(1,3-propanediyl)bis-	792146	5.7	C15H16	Diarylalkane	Oligomers	0
35.3	Naphthalene, 1,2,3,4-tetrahydro-2-phenyl-	2470308.8	17.6	C16H16	Partially hydrogenated PAH	PAHs	0

35.6	Anthracene, 9,10-dihydro-	628681.6	4.5	C14H12	Partially hydrogenated PAH	PAHs	0
		14008584.3					0.0224200

Table S12. GC-MS analysis result of oil produced during catalytic pyrolysis of sewage sludge char with PS with rate 1:4.

RT	Name	Area	ω, %	Formula	Category	Group	Oxygen content
4.5	Water	200886	0.5	H2O	Inorganic solvent	Other	0.0044405
12.3	Benzene	1201115	2.9	C6H6	Aromatic hydrocarbon	Light aromatic fraction	0
15.2	Toluene	812179	2.0	C7H8	Alkylbenzene	Light aromatic fraction	0
17.4	Benzene, 1,3-dimethyl-	460113	1.1	C8H10	Alkylbenzene	Light aromatic fraction	0
18.2	Styrene	17618727	42.6	C8H8	Vinyl aromatic hydrocarbons	Light aromatic fraction	0
19.3	Benzeneethanol, .beta.-ethenyl-	42128	0.1	C10H12O	Aromatic alcohol	Oxygen-containing compounds	0.0001080
20.0	.alpha.-Methylstyrene	1755342	4.2	C9H10	Vinyl aromatic hydrocarbons	Light aromatic fraction	0
20.2	Benzaldehyde	52374	0.1	C7H6O	Aromatic aldehyde	Oxygen-containing compounds	0.0001508
20.2	Phenacylidene diacetate	53458	0.1	C12H12O4	Aromatic ester	Oxygen-containing compounds	0.0002906
20.3	Diglycolic acid, di(3-phenylpropyl) ester	25040	0.1	C22H26O5	Diester	Oxygen-containing compounds	0.0002159
20.9	Benzene, cyclopropyl-	156386	0.4	C9H10	Cycloalkylbenzene	Cyclic unsaturated compounds	0
21.6	Indene	412499	1.0	C9H8	Indene	Cyclic unsaturated compounds	0
22.6	Naphthalene, 1,2-dihydro-	49866	0.1	C10H10	Partially hydrogenated PAH	Cyclic unsaturated compounds	0

22.8	Naphthalene, 1-phenyl-	237494	0.6	C16H12	PAH	PAHs	0
23.4	Cycloprop[a]indene, 1,1a,6,6a-tetrahydro-	35852	0.1	C10H10	Fused cyclic unsaturated hydrocarbon	Cyclic unsaturated compounds	0
23.5	2-Methylindene	93571	0.2	C10H10	Alkyl-substituted indene	Cyclic unsaturated compounds	0
23.7	1,4-Dihydronaphthalene	73878	0.2	C10H10	Partially hydrogenated PAH	Cyclic unsaturated compounds	0
24.3	Naphthalene	1646678	4.0	C10H8	PAH	PAHs	0
26.0	Naphthalene, 2-methyl-	71883	0.2	C11H10	PAH	PAHs	0
26.4	1H-Indene, 1-ethylidene-	71367	0.2	C11H10	Alkenyl-substituted indene	Cyclic unsaturated compounds	0
27.3	Biphenyl	1575667	3.8	C12H10	Biphenyl	Oligomers	0
28.1	Diphenylmethane	96690	0.2	C13H12	Diarylmethane	Oligomers	0
28.5	Acenaphthene	51726	0.1	C12H10	Partially hydrogenated PAH	PAHs	0
29.2	1,1-Diphenyl-2-propanol	92025	0.2	C15H16 O	Diaryl alcohol	Oxygen-containing compounds	0.0001507
29.4	Acenaphthylene	84871	0.2	C12H8	PAH	PAHs	0
29.6	(E)-Stilbene	189333	0.5	C14H12	Diarylalkene	Oligomers	0
29.9	cis-Stilbene	93394	0.2	C14H12	Diarylalkene	Oligomers	0
30.0	Bibenzyl	326135	0.8	C14H14	Diarylalkane	Oligomers	0
30.6	Benzene, 1,1'-(1-methyl-1,2-ethanediyl)bis-	252927	0.6	C15H16	Diarylalkane	Oligomers	0
30.6	Benzene, 1,1'-(1,2-dimethyl-1,2-ethanediyl)bis-, (R*,R*)-(.+/-.)-	240417	0.6	C16H18	Diarylalkane	Oligomers	0
31.3	1H-Indene, 1-(phenylmethylene)-	619017	1.5	C16H12	Diaryl-substituted indene	Cyclic unsaturated compounds	0
32.1	1,1'-Biphenyl, 4-ethenyl-	188436	0.5	C14H12	Alkenylbiphenyl	Oligomers	0
32.4	Fluorene	370057	0.9	C13H10	PAH	PAHs	0
32.9	Benzene, 1,1',1'',1'''-(1,2,3,4-cyclobutanetetrayl)tetrakis-	121260	0.3	C28H28	Polyaryl hydrocarbon	Oligomers	0
33.2	Benzene, 1,1'-(1,3-propanediyl)bis-	2181042	5.3	C15H16	Diarylalkane	Oligomers	0
34.0	Benzene, 1,1'-(1,2-ethanediyl)bis[2-methyl-	68269	0.2	C16H18	Alkylated diarylalkane	Oligomers	0

35.3	Naphthalene, 1,2,3,4-tetrahydro-2-phenyl-	8127649	19.6	C16H16	Partially hydrogenated PAH	PAHs	0
35.6	Anthracene, 9,10-dihydro-	1365562	3.3	C14H12	Partially hydrogenated PAH	PAHs	0
39.4	Benzene, 1,1'-(1-butene-1,4-diyl)bis-, (Z)-	276014	0.7	C16H16	Diarylalkene	Oligomers	0
		41391327					0.0053565

Table S13. GC-MS analysis result of oil produced during catalytic pyrolysis of sewage sludge char with PS with rate 1:10.

RT	Name	Area	ω, %	Formula	Category	Group	Oxygen content
4.5	Water	294946	0.8	H2O	Inorganic solvent	Other	0.0071047
12.3	Benzene	3429076	9.2	C6H6	Aromatic hydrocarbon	Light aromatic fraction	0
15.2	Toluene	1737513	4.7	C7H8	Alkylbenzene	Light aromatic fraction	0
17.4	Benzene, 1,3-dimethyl-	474033	1.3	C8H10	Alkylbenzene	Light aromatic fraction	0
18.2	Styrene	12155132	32.7	C8H8	Vinyl aromatic hydrocarbon	Light aromatic fraction	0
19.3	Diglycolic acid, di(3-phenylpropyl) ester	25384	0.1	C22H26 O5	Diester	Oxygen-containing compounds	0.0002159
20.0	.alpha.-Methylstyrene	914488	2.5	C9H10	Vinyl aromatic hydrocarbon	Light aromatic fraction	0
20.2	Fumaric acid, di(3-phenylpropyl) ester	14758	0.0	C20H22 O4	Diester	Oxygen-containing compounds	0
20.2	Benzaldehyde	352243	0.9	C7H6O	Aromatic aldehyde	Oxygen-containing compounds	0.0013568
20.3	Pimelic acid, di(3-phenylpropyl) ester	27326	0.1	C20H24 O4	Diester	Oxygen-containing compounds	0.0001949
20.9	Benzene, cyclopropyl-	102314	0.3	C9H10	Cycloalkylbenz ene	Cyclic unsaturated compounds	0
21.6	3-Methylphenylacetylene	428304	1.2	C9H8	Alkynylalkylbe nzene	Cyclic unsaturated compounds	0

22.3	Acetophenone	42433	0.1	C8H8O	Aromatic ketone	Oxygen-containing compounds	0.0001332
22.6	Naphthalene, 1,2-dihydro-	53724	0.1	C10H10	Partially hydrogenated PAH	Cyclic unsaturated compounds	0
22.8	1H-Indene, 1-(phenylmethylene)-	377962	1.0	C16H12	Diaryl-substituted indene	Cyclic unsaturated compounds	0
22.8	Benzene, 1,4-diethenyl-	46130	0.1	C10H10	Alkenylbenzen e	Light aromatic fraction	0
23.4	Benzene, 1-methyl-4-(1-propynyl)-	36002	0.1	C10H10	Alkynylalkylbe nzene	Cyclic unsaturated compounds	0
23.5	2-Methylindene	85981	0.2	C10H10	Alkyl-substituted indene	Cyclic unsaturated compounds	0
23.7	Cycloprop[a]indene, 1,1a,6,6a-tetrahydro-	66542	0.2	C10H10	Fused cyclic unsaturated hydrocarbon	Cyclic unsaturated compounds	0
24.3	Naphthalene	1818605	4.9	C10H8	PAH	PAHs	0
25.0	Benzeneethanol, .beta.-methylene-	116170	0.3	C10H10 O	Aromatic alcohol	Oxygen-containing compounds	0.0003283
26.0	1H-Indene, 1-ethylidene-	73129	0.2	C11H10	Alkenyl-substituted indene	Cyclic unsaturated compounds	0
26.4	Naphthalene, 2-methyl-	65633	0.2	C11H10	Alkylnaphthale ne	PAHs	0
27.3	Biphenyl	1754631	4.7	C12H10	Biphenyl	Oligomers	0
28.1	Diphenylmethane	114871	0.3	C13H12	Diarylmethane	Oligomers	0
29.2	1,1'-Biphenyl, 4-methyl-	128880	0.3	C13H12	Alkylbiphenyl	Oligomers	0
29.4	Acenaphthylene	103702	0.3	C12H8	PAH	PAHs	0
29.6	Ethylene, 1,1-diphenyl-	218556	0.6	C14H12	Diarylalkene	Oligomers	0
29.9	(E)-Stilbene	148359	0.4	C14H12	Diarylalkene	Oligomers	0
30.1	Bibenzyl	321071	0.9	C14H14	Diarylalkane	Oligomers	0
30.7	Benzene, 1,1'-(1,2-dimethyl-1,2-ethanediyl)bis-, (R*,S*)-	158372	0.4	C16H18	Diarylalkane	Oligomers	0
31.3	Acephenanthrylene, 4,5-dihydro-	609154	1.6	C16H12	Partially hydrogenated PAH	PAHs	0
32.1	1,1'-Biphenyl, 4-ethenyl-	292522	0.8	C14H12	Alkenylbiphen yl	Oligomers	0

32.4	Fluorene	486030	1.3	C13H10	PAH	PAHs	0
32.9	Benzene, 1,1',1'',1'''-(1,2,3,4-cyclobutanetetrayl)tetrakis-	162285	0.4	C28H28	Polyaryl hydrocarbon	Oligomers	0
33.2	Benzene, 1,1'-(1,3-propanediyl)bis-	1805736	4.9	C15H16	Diarylalkane	Oligomers	0
34.0	Benzene, 1,1'-(1-methyl-1,3-propanediyl)bis-	74062	0.2	C16H18	Diarylalkane	Oligomers	0
35.3	Naphthalene, 1,2,3,4-tetrahydro-2-phenyl-	5402061	14.5	C16H16	Partially hydrogenated PAH	PAHs	0
35.6	Anthracene, 9,10-dihydro-	2104642	5.7	C14H12	Partially hydrogenated PAH	PAHs	0
35.8	1,2-Diphenylcyclopropane	350223	0.9	C15H14	Diarylcycloalkane	Oligomers	0
39.4	Benzene, 1,1'-(1-butene-1,4-diyl)bis-, (Z)-	203674	0.5	C16H16	Diarylalkene	Oligomers	0
		37176659					0.0093338

Table S14. GC-MS analysis result of oil produced during catalytic pyrolysis of acid-leached Fe-impregnated sewage sludge char with PS rate 1:1.

RT	Name	Area	ω, %	Formula	Category	Group	Oxygen content
3.50	Nitrogen	88645.7	0.12	N2	Inorganic gas	Other	0
4.50	Water	1376258.5	1.88	H2O	Inorganic solvent	Other	0.0166962
10.03	(S)-(+)-1,2-Propanediol	184098.7	0.25	C3H8O2	Aliphatic diol	Oxygen-containing compounds	0.0010513
12.36	Benzene	11617702.4	15.89	C6H6	Aromatic hydrocarbons	Light aromatic fraction	0
15.26	Toluene	4558485.5	6.24	C7H8	Alkylbenzene	Light aromatic fraction	0
16.21	Propane, 2,2'-[ethylidenebis(oxy)]bis-	240481.9	0.33	C7H16O2	Aliphatic ether	Oxygen-containing compounds	0.0007987
17.51	Ethylbenzene	1433834.4	1.96	C8H10	Alkylbenzene	Light aromatic fraction	0
18.30	Styrene	23110073.8	31.61	C8H8	Aromatic alkene	Light aromatic fraction	0
18.83	Benzene, (1-methylethyl)-	61573.4	0.08	C9H12	Alkylbenzene	Light aromatic fraction	0
19.33	Benzeneethanol, .beta.-ethenyl-	79507.5	0.11	C10H12O	Aromatic unsaturated alcohol	Oxygen-containing compounds	0.0001187

20.05	.alpha.-Methylstyrene	3567370.4	4.88	C9H10	Aromatic alkene	Light aromatic fraction	0
20.25	Pimelic acid, di(3-phenylpropyl) ester	39278.7	0.05	C20H24O4	Aromatic diester	Oxygen-containing compounds	0.0000974
20.31	Benzaldehyde	1114122.2	1.52	C7H6O	Aromatic aldehyde	Oxygen-containing compounds	0.0022915
20.39	Fumaric acid, di(3-phenylpropyl) ester	57627.3	0.08	C20H22O4	Aromatic diester	Oxygen-containing compounds	0.0001569
20.47	Diglycolic acid, di(3-phenylpropyl) ester	20307.6	0.03	C22H26O5	Aromatic diester	Oxygen-containing compounds	0.0000648
21.00	Benzene, 2-propenyl-	316223.5	0.43	C9H10	Alkenylbenzene	Light aromatic fraction	0
21.28	2-Vinylfuran	39425.6	0.05	C6H6O	Heteroaromatic compound	Oxygen-containing compounds	0.0000850
21.28	Phenol	41347.1	0.06	C6H6O	Aromatic phenol	Oxygen-containing compounds	0.0001020
21.31	Benzaldehyde, 4-(1-phenyl-2-propenyloxy)-	28495.5	0.04	C16H14O2	Aromatic aldehyde	Oxygen-containing compounds	0.0000537
21.65	Indene	919542.8	1.26	C9H8	Indene	Cyclic unsaturated compounds	0
21.87	Benzeneacetaldehyde	218845.2	0.30	C8H8O	Aromatic aldehyde	Oxygen-containing compounds	0.0003995
22.32	Acetophenone	233478.7	0.32	C8H8O	Aromatic ketone	Oxygen-containing compounds	0.0004261
22.65	Naphthalene, 1,2-dihydro-	54338	0.07	C10H10	Partially hydrogenated PAH	Cyclic unsaturated compounds	0
22.85	Benzeneacetaldehyde, .alpha.-methyl-	46265.1	0.06	C9H10O	Aromatic aldehyde	Oxygen-containing compounds	0.0000715
22.89	2-Methylindene	21037.4	0.03	C10H10	Alkyl-substituted indene	Cyclic unsaturated compounds	0

23.47	Cycloprop[a]indene, 1,1a,6,6a-tetrahydro-	222532.7	0.30	C10H10	Fused cyclic unsaturated hydrocarbon	Cyclic unsaturated compounds	0
23.62	1H-Indene, 3-methyl-	107915	0.15	C10H10	Alkyl-substituted indene	Cyclic unsaturated compounds	0
23.85	1,4-Dihydronaphthalene	304575	0.42	C10H10	Partially hydrogenated PAH	Cyclic unsaturated compounds	0
24.38	Naphthalene	4219896.7	5.77	C10H8	PAH	PAHs	0
26.14	1H-Indene, 1-ethylidene-	244210.1	0.33	C11H10	Alkenyl-substituted indene	Cyclic unsaturated compounds	0
26.48	Naphthalene, 2-methyl-	257706.2	0.35	C11H10	PAH	PAHs	0
27.40	Biphenyl	3665433.9	5.01	C12H10	Biaryl aromatic hydrocarbon	Oligomers	0
27.49	Benzene, 1,1',1'',1'''-(1,2-ethanediylidene)tetrakis-	60553.8	0.08	C26H20	Polyaryl hydrocarbon	Oligomers	0
28.24	Diphenylmethane	339346	0.46	C13H12	Diarylalkane	Oligomers	0
28.64	Naphthalene, 2-ethenyl-	106425.6	0.15	C12H10	Alkenyl-substituted PAH	PAHs	0
29.32	1,1'-Biphenyl, 4-methyl-	374203.5	0.51	C13H12	Alkyl-substituted biaryl	Oligomers	0
29.51	Acenaphthylene	226433.5	0.31	C12H8	PAH	PAHs	0
29.59	1,1'-Biphenyl, 3-methyl-	155234.3	0.21	C13H12	Alkyl-substituted biaryl	Oligomers	0
29.78	Ethylene, 1,1-diphenyl-	341322.9	0.47	C14H12	Diarylalkene	Oligomers	0
29.78	cis-Stilbene	337145.4	0.46	C14H12	Diarylalkene	Oligomers	0
30.08	Anthracene, 9,10-dihydro-	222595.2	0.30	C14H12	Partially hydrogenated PAH	PAHs	0
30.21	Benzyl sulfone	220416.3	0.30	C14H14 O2S	Aromatic sulfone	Oxygen-containing compounds	0.0003897
30.82	Benzene, 1,1'-(1-methyl-1,2-ethanediyl)bis-	526794.6	0.72	C15H16	Diarylalkane	Oligomers	0
31.01	2,2,6,6-Tetramethylheptane	192533.5	0.26	C11H24	Branched alkane	Other	0
32.33	1,1'-Biphenyl, 4-ethenyl-	364714.6	0.50	C14H12	Alkenylbiphenyl	Oligomers	0
32.61	Fluorene	964018.8	1.32	C13H10	PAH	PAHs	0

33.10	Phenanthrene, 9,10-dihydro-	192068.9	0.26	C14H12	Partially hydrogenated PAH	PAHs	0
33.42	Benzene, 1,1'-(1,3-propanediyl)bis-	2646616.2	3.62	C15H16	Diarylalkane	Oligomers	0
33.68	Benzene, 1,1'-(1,2-dimethyl-1,2-ethenediyl)bis-, (Z)-	647626.7	0.89	C16H16	Diarylalkene	Oligomers	0
34.22	Benzene, 1,1'-(1-methyl-1,3-propanediyl)bis-	87457.1	0.12	C16H18	Diarylalkane	Oligomers	0
35.86	(E)-Stilbene	3171954	4.34	C14H12	Diarylalkene	Oligomers	0
36.04	1,2-Diphenylcyclopropane	1463759.4	2.00	C15H14	Diarylcycloalkane	Oligomers	0
		73107771					0.0228030

Table S15. GC-MS analysis result of oil produced during catalytic pyrolysis of acid-leached Fe-impregnated sewage sludge char with PS rate 1:2.

RT	Name	Area	ω, %	Formula	Category	Group	Oxygen content
4.5	Water	213332	0.8	H2O	Inorganic solvent	Other	0.0071047
12.3	Benzene	1472460	5.2	C6H6	Aromatic hydrocarbon	Light aromatic fraction	0
15.2	Toluene	1012811	3.6	C7H8	Alkylbenzene	Light aromatic fraction	0
17.4	Benzene, 1,3-dimethyl-	548025	1.9	C8H10	Alkylbenzene	Light aromatic fraction	0
18.2	Styrene	12421253	43.9	C8H8	Vinyl aromatic hydrocarbons	Light aromatic fraction	0
19.3	Benzeneethanol, .beta.-ethenyl-	29882	0.1	C10H12O	Aromatic unsaturated alcohol	Oxygen-containing compounds	0.0001080
20.0	.alpha.-Methylstyrene	1758704	6.2	C9H10	Vinyl aromatic hydrocarbons	Light aromatic fraction	0
20.2	Diglycolic acid, di(3-phenylpropyl) ester	13691	0.0	C22H26O5	Aromatic diester	Oxygen-containing compounds	0
20.9	Benzene, cyclopropyl-	108746	0.4	C9H10	Cycloalkylbenzene	Cyclic unsaturated compounds	0
22.8	1H-Indene, 1-(phenylmethylene)-	494269	1.7	C16H12	Diaryl-substituted indene	Cyclic unsaturated compounds	0

24.3	1H-Indene, 2-phenyl-	403321	1.4	C15H12	PAH-related aromatic hydrocarbon	Cyclic unsaturated compounds	0
24.3	Naphthalene	736162	2.6	C10H8	PAH	PAHs	0
24.6	Oxalic acid, hexadecyl hexyl ester	176285	0.6	C24H48O4	Aliphatic diester	Oxygen-containing compounds	0.0009584
26.0	Succinic acid, 2,4,6-trichlorophenyl 2-naphthylmethyl ester	31970	0.1	C21H15Cl3O4	Halogenated aromatic diester	Oxygen-containing compounds	0.0001462
26.4	Naphthalene, 2-methyl-	39945	0.1	C11H10	Alkyl-substituted PAH	PAHs	0
27.3	Biphenyl	727342	2.6	C12H10	Biaryl aromatic hydrocarbon	Oligomers	0
28.1	Benzene, 1,1',1'',1'''-(1,2-ethanediylidene)tetrakis-	51846	0.2	C26H20	Polyaryl hydrocarbon	Oligomers	0
29.2	1,1'-Biphenyl, 3-methyl-	53597	0.2	C13H12	Alkyl-substituted biaryl	Oligomers	0
29.6	(E)-Stilbene	108444	0.4	C14H12	Diarylalkene	Oligomers	0
29.9	cis-Stilbene	50028	0.2	C14H12	Diarylalkene	Oligomers	0
30.0	Bibenzyl	226913	0.8	C14H14	Diarylalkane	Oligomers	0
30.6	Benzene, 1,1'-(1-methyl-1,2-ethanediyl)bis-	225943	0.8	C15H16	Diarylalkane	Oligomers	0
30.6	Benzene, 1,1'-(1,2-dimethyl-1,2-ethanediyl)bis-, (R*,S*)-	230369	0.8	C16H18	Diarylalkane	Oligomers	0
31.3	Naphthalene, 2-phenyl-	1350183	4.8	C16H12	PAH	PAHs	0
32.1	1,1'-Biphenyl, 4-ethenyl-	87635	0.3	C14H12	Alkenylbiphenyl	Oligomers	0
32.4	Fluorene	195578	0.7	C13H10	PAH	PAHs	0
32.9	Benzene, 1,1',1'',1'''-(1,2,3,4-cyclobutanetetrayl)tetrakis-	44941	0.2	C28H28	Polyaryl hydrocarbon	Oligomers	0
33.2	Benzene, 1,1'-(1,3-propanediyl)bis-	1765639	6.2	C15H16	Diarylalkane	Oligomers	0
33.5	1-Propene, 2-(2-methylphenyl)-1-phenyl-, (Z)-	453846	1.6	C16H16	Diarylalkene	Oligomers	0
33.6	Stilbene, .alpha.-methyl-, (E)-	75343	0.3	C15H14	Alkyl-substituted diarylalkene	Oligomers	0
34.0	Benzene, 1,1'-(1-methyl-1,3-propanediyl)bis-	98488	0.3	C16H18	Diarylalkane	Oligomers	0
35.6	Anthracene, 9,10-dihydro-	805316	2.8	C14H12	Hydrogenated PAH	PAHs	0

35.8	1,2-Diphenylcyclopropane	667107	2.4	C15H14	Diarylcycloalkane	Oligomers	0
37.8	1H-Indene, 1-phenyl-	56452	0.2	C15H12	PAH-related aromatic hydrocarbon	Cyclic unsaturated compounds	0
38.0	Benzene, 1,1'-(3-methyl-1-propene-1,3-diyl)bis-	1317249.3	4.7	C16H16	Diarylalkene	Oligomers	0
39.3	Benzene, 1,1'-(1-butene-1,4-diyl)bis-, (Z)-	215477.7	0.8	C16H16	Diarylalkene	Oligomers	0
		28268589					0.0083173

Table S16. GC-MS analysis result of oil produced during catalytic pyrolysis of acid-leached Fe-impregnated sewage sludge char with PS rate 1:4.

RT	Name	Area	ω, %	Formula	Category	Group	Oxygen content
4.5	Water	187438	0.6	H2O	Inorganic solvent	Other	0.0053285
12.3	Benzene	1579474	5.1	C6H6	Aromatic hydrocarbon	Light aromatic fraction	0
15.2	Toluene	923878	3.0	C7H8	Alkylbenzene	Light aromatic fraction	0
17.4	Benzene, (propoxymethyl)-	284591	0.9	C10H14O	Alkoxyalkylbenzene	Oxygen-containing compounds	0.0009718
17.4	Ethylbenzene	462142	1.5	C8H10	Alkylbenzene	Light aromatic fraction	0
18.2	Styrene	12332934	39.5	C8H8	Vinyl aromatic hydrocarbon	Light aromatic fraction	0
20.0	.alpha.-Methylstyrene	1262628	4.0	C9H10	Vinyl aromatic hydrocarbon	Light aromatic fraction	0
20.9	Indane	89494	0.3	C9H10	Partially hydrogenated PAH	Cyclic unsaturated compounds	0
21.6	Indene	300349	1.0	C9H8	Indene	Cyclic unsaturated compounds	0
22.6	2-Methylindene	32837	0.1	C10H10	Alkyl-substituted indene	Cyclic unsaturated compounds	0
22.7	Anthracene, 9-ethenyl-	559575	1.8	C16H12	Alkenyl-substituted PAH	PAHs	0

23.4	Cycloprop[a]indene, 1,1a,6,6a-tetrahydro-	26619	0.1	C10H10	Fused cyclic unsaturated hydrocarbon	Cyclic unsaturated compounds	0
23.7	Naphthalene, 1,2-dihydro-	55498	0.2	C10H10	Partially hydrogenated PAH	Cyclic unsaturated compounds	0
24.3	Naphthalene	1187056	3.8	C10H8	PAH	PAHs	0
26.0	Naphthalene, 2-methyl-	44013	0.1	C11H10	PAH	PAHs	0
26.4	Naphthalene, 1-methyl-	45074	0.1	C11H10	PAH	PAHs	0
27.3	Biphenyl	1082927	3.5	C12H10	Biaryl aromatic hydrocarbon	Oligomers	0
28.1	Benzene, 1,1',1'',1'''-(1,2-ethanediylidene)tetrakis-	74696	0.2	C26H20	Polyaryl hydrocarbon	Oligomers	0
28.5	Acenaphthene	30180	0.1	C12H10	Partially hydrogenated PAH	PAHs	0
29.3	Acenaphthylene	54203	0.2	C12H8	PAH	PAHs	0
29.6	Ethylene, 1,1-diphenyl-	143904	0.5	C14H12	Diarylalkene	Oligomers	0
29.9	(E)-Stilbene	79259	0.3	C14H12	Diarylalkene	Oligomers	0
30.0	Bibenzyl	170170	0.5	C14H14	Diarylalkane	Oligomers	0
30.6	Benzene, 1,1'-(1,2-dimethyl-1,2-ethanediyl)bis-	108914	0.3	C16H18	Diarylalkane	Oligomers	0
30.6	Benzene, 1,1'-(1,2-dimethyl-1,2-ethanediyl)bis-, (R*,S*)-	119715	0.4	C16H18	Diarylalkane	Oligomers	0
31.3	1H-Indene, 1-(phenylmethylene)-	1089818	3.5	C16H12	Diaryl-substituted indene	Oligomers	0
32.1	1,1'-Biphenyl, 4-ethenyl-	165758	0.5	C14H12	Alkenylbiphenyl	Oligomers	0
32.4	Fluorene	305910	1.0	C13H10	PAH	PAHs	0
32.9	Benzene, 1,1',1'',1'''-(1,2,3,4-cyclobutanetetrayl)tetrakis-	86529	0.3	C28H28	Polyaryl hydrocarbon	Oligomers	0
33.2	Benzene, 1,1'-(1,3-propanediyl)bis-	1712873	5.5	C15H16	Diarylalkane	Oligomers	0
34.0	Benzene, 1,1'-(1,2-ethanediyl)bis[2-methyl-	58290	0.2	C16H18	Alkyl-substituted diarylalkane	Oligomers	0
35.3	Naphthalene, 1,2,3,4-tetrahydro-2-phenyl-	4159697	13.3	C16H16	Partially hydrogenated PAH	PAHs	0
35.6	Anthracene, 9,10-dihydro-	1245721	4.0	C14H12	Partially hydrogenated PAH	PAHs	0

35.8	1,2-Diphenylcyclopropane	354991	1.1	C15H14	Diarylcycloalkane	Oligomers	0
38.0	Benzene, 1,1'-(3-methyl-1-propene-1,3-diyl)bis-	643472	2.1	C16H16	Diarylalkene	Oligomers	0
39.4	Benzene, 1,1'-(1-butene-1,4-diyl)bis-, (Z)-	124419	0.4	C16H16	Diarylalkene	Oligomers	0
		31185043					0.0063003

Table S17. GC-MS analysis result of oil produced during catalytic pyrolysis of acid-leached Fe-impregnated sewage sludge char with PS rate 1:10.

RT	Name	Area	ω, %	Formula	Category	Group	Oxygen content
4.5	Water	161823.4	0.3	H2O	Inorganic solvent	Other	0.0026643
12.3	Benzene	1081948.9	2.0	C6H6	Aromatic hydrocarbon	Light aromatic fraction	0
15.2	Toluene	1090947.2	2.0	C7H8	Alkylbenzene	Light aromatic fraction	0
17.4	Ethylbenzene	962147	1.8	C8H10	Alkylbenzene	Light aromatic fraction	0
18.2	Styrene	25512772.5	46.8	C8H8	Vinyl aromatic hydrocarbon	Light aromatic fraction	0
18.8	Benzene, (1-methylethyl)-	53678.4	0.1	C9H12	Alkylbenzene	Light aromatic fraction	0
18.8	1-Hexanone, 5-methyl-1-phenyl-	45302.2	0.1	C13H18O	Aromatic ketone	Oxygen-containing compounds	0.0000841
19.3	Indane	61188.8	0.1	C9H10	Indane	Cyclic unsaturated compounds	0
19.4	Benzeneacetaldehyde	20224.6	0.0	C8H8O	Aromatic aldehyde	Oxygen-containing compounds	0
20.0	.alpha.-Methylstyrene	2422934	4.4	C9H10	Vinyl aromatic hydrocarbon	Light aromatic fraction	0
20.2	Phenacylidene diacetate	39261.6	0.1	C12H12O4	Aromatic ester	Oxygen-containing compounds	0.0002906
20.3	Benzene, 1,1'-(1-ethenyl-1,3-propanediyl)bis-	37313.9	0.1	C17H18	Diarylalkene	Oligomers	0
20.4	Benzeneethanol, .beta.-ethenyl-.alpha.-methyl-	13439.3	0.0	C11H14O	Aromatic unsaturated alcohol	Oxygen-containing compounds	0

20.9	Benzene, cyclopropyl-	205801.7	0.4	C9H10	Cycloalkylbenzene	Light aromatic fraction	0
21.6	Benzene, 1-propynyl-	506667.7	0.9	C9H8	Alkynylbenzene	Light aromatic fraction	0
22.6	1H-Indene, 3-methyl-	48494.7	0.1	C10H10	Alkyl-substituted indene	Cyclic unsaturated compounds	0
22.7	1H-Indene, 1-(phenylmethylene)-	364926	0.7	C16H12	Diaryl-substituted indene	Oligomers	0
23.4	2-Methylindene	50458.9	0.1	C10H10	Alkyl-substituted indene	Cyclic unsaturated compounds	0
23.5	Cycloprop[a]indene, 1,1a,6,6a-tetrahydro-	106595	0.2	C10H10	Fused cyclic unsaturated hydrocarbon	Cyclic unsaturated compounds	0
23.7	Naphthalene, 1,2-dihydro-	88573.5	0.2	C10H10	Partially hydrogenated PAH	Cyclic unsaturated compounds	0
24.3	Naphthalene	1972945.1	3.6	C10H8	PAH	PAHs	0
26.0	Naphthalene, 2-methyl-	92097.1	0.2	C11H10	PAH	PAHs	0
26.4	1H-Indene, 1-ethylidene-	77385.4	0.1	C11H10	Alkenyl-substituted indene	Cyclic unsaturated compounds	0
27.3	Biphenyl	1631510.6	3.0	C12H10	Biaryl aromatic hydrocarbon	Oligomers	0
28.1	1,1'-Biphenyl, 2-methyl-	121126	0.2	C13H12	Alkyl-substituted biaryl	Oligomers	0
28.5	Acenaphthene	55613.8	0.1	C12H10	Partially hydrogenated PAH	PAHs	0
29.2	1,1'-Biphenyl, 4-methyl-	120587.7	0.2	C13H12	Alkyl-substituted biaryl	Oligomers	0
29.3	Acenaphthylene	121572	0.2	C12H8	PAH	PAHs	0
29.6	Ethylene, 1,1-diphenyl-	179013.1	0.3	C14H12	Diarylalkene	Oligomers	0
29.9	Anthracene, 9,10-dihydro-9-(1-methylpropyl)-	56294.2	0.1	C18H20	Partially hydrogenated PAH	PAHs	0
30.0	Bibenzyl	325911.1	0.6	C14H14	Diarylalkane	Oligomers	0
30.6	Benzene, 1,1'-(1,2-dimethyl-1,2-ethanediyl)bis-, (R*,R*)-(.+/-.)-	230674.7	0.4	C16H18	Diarylalkane	Oligomers	0

31.3	Acephenanthrylene, 4,5-dihydro-	719000	1.3	C16H12	Partially hydrogenated PAH	PAHs	0
32.1	1,1'-Biphenyl, 4-ethenyl-	164801.3	0.3	C14H12	Alkenylbiphenyl	Oligomers	0
32.4	Fluorene	369687.7	0.7	C13H10	PAH	PAHs	0
32.9	(E)-Stilbene	86446.6	0.2	C14H12	Diarylalkene	Oligomers	0
33.2	Benzene, 1,1'-(1,3-propanediyl)bis-	3051959.7	5.6	C15H16	Diarylalkane	Oligomers	0
35.3	Naphthalene, 1,2,3,4-tetrahydro-2-phenyl-	9715876	17.8	C16H16	Partially hydrogenated PAH	PAHs	0
35.6	Anthracene, 9,10-dihydro-	1132013.5	2.1	C14H12	Partially hydrogenated PAH	PAHs	0
35.8	1,2-Diphenylcyclopropane	550101.9	1.0	C15H14	Diarylcycloalkane	Oligomers	0
38.04	Benzene, 1,1'-(3-methyl-1-propene-1,3-diyl)bis-	468891.3	0.9	C16H16	Diarylalkene	Oligomers	0
39.35	Benzene, 1,1'-(1-butene-1,4-diyl)bis-, (Z)-	347841	0.6	C16H16	Diarylalkene	Oligomers	0
		54465849.1					0.0030390

Table S18. GC-MS analysis result of oil produced during catalytic pyrolysis of acid-leached Fe-impregnated sewage sludge char after one cycle of use with PS rate 1:4.

RT	Name	Area	ω, %	Formula	Category	Group	Oxygen content
4.5	Water	379022	1.1	H2O	Inorganic solvent	Other	0.97690
12.3	Benzene	1000645	2.8	C6H6	Aromatic hydrocarbon	Light aromatic fraction	0
15.2	Toluene	1066993	3.0	C7H8	Alkylbenzene	Light aromatic fraction	0
17.4	Benzene, 1,3-dimethyl-	497398	1.4	C8H10	Alkylbenzene	Light aromatic fraction	0
18.2	Styrene	13932905	39.2	C8H8	Vinyl aromatic hydrocarbon	Light aromatic fraction	0
19.3	3-Phenyl-1-propanol, acetate	27195	0.1	C11H14O2	Aromatic ester	Oxygen-containing compounds	0.01795
19.3	Tricyclo[3.2.1.0(2,,4)]oct-6-ene, 8-methylene-, (1.alpha.,2.alpha.,4.alpha.,5.alpha.)-	27666	0.1	C9H10	Polycyclic unsaturated hydrocarbon	Cyclic unsaturated compounds	0

20.0	.alpha.-Methylstyrene	1617689	4.5	C9H10	Vinyl aromatic hydrocarbon	Light aromatic fraction	0
20.2	Benzaldehyde, 4-(1-phenyl-2-propenyloxy)-	12899	0.0	C16H14O2	Aromatic aldehyde	Oxygen-containing compounds	0
20.2	Benzaldehyde	111596	0.3	C7H6O	Aromatic aldehyde	Oxygen-containing compounds	0.04523
20.3	Benzeneethanol, .beta.-ethenyl-	20596	0.1	C10H12O	Vinyl-substituted aromatic alcohol	Oxygen-containing compounds	0.01080
20.9	Benzene, cyclopropyl-	113826	0.3	C9H10	Alkylbenzene	Light aromatic fraction	0
21.6	Indene	327868	0.9	C9H8	Indene	Cyclic unsaturated compounds	0
22.6	2-Methylindene	39314	0.1	C10H10	Alkyl-substituted indene	Cyclic unsaturated compounds	0
22.8	Naphthalene, 1-phenyl-	425906	1.2	C16H12	PAH	PAHs	0
23.7	Naphthalene, 1,2-dihydro-	42441	0.1	C10H10	Partially hydrogenated PAH	Cyclic unsaturated compounds	0
24.3	Naphthalene	1292713	3.6	C10H8	PAH	PAHs	0
26.0	Naphthalene, 1-methyl-	52385	0.1	C11H10	PAH	PAHs	0
26.4	Naphthalene, 2-methyl-	45538	0.1	C11H10	PAH	PAHs	0
27.3	Biphenyl	1084996	3.0	C12H10	Biphenyl hydrocarbon	Oligomers	0
28.1	Diphenylmethane	91408	0.3	C13H12	Diaryl alkane	Oligomers	0
29.2	Benzene, 1,1',1'',1'''-(1,2-ethanediylidene)tetrakis-	77618	0.2	C26H20	Polyaryl hydrocarbon	Oligomers	0
29.4	Acenaphthylene	58199	0.2	C12H8	PAH	PAHs	0
29.6	(E)-Stilbene	156097	0.4	C14H12	Diaryl alkene	Oligomers	0
29.9	cis-Stilbene	75752	0.2	C14H12	Diaryl alkene	Oligomers	0
30.1	Bibenzyl	167923	0.5	C14H14	Diaryl alkane	Oligomers	0
30.6	Benzene, 1,1'-(1,2-dimethyl-1,2-ethanediyl)bis-, (R*,S*)-	123040	0.3	C16H18	Diaryl alkane	Oligomers	0
31.3	1H-Indene, 1-(phenylmethylene)-	676328	1.9	C16H12	Diaryl-substituted indene	Oligomers	0
32.1	1,1'-Biphenyl, 4-ethenyl-	160441	0.5	C14H12	Alkenylbiphenyl	Oligomers	0

32.4	Fluorene	324279	0.9	C13H10	PAH	PAHs	0
33.2	Benzene, 1,1'-(1,3-propanediyl)bis-	2283546	6.4	C15H16	Diaryl alkane	Oligomers	0
34.0	Benzene, 1,1'-(1-methyl-1,3-propanediyl)bis-	128152	0.4	C16H18	Diaryl alkane	Oligomers	0
35.3	Naphthalene, 1,2,3,4-tetrahydro-2-phenyl-	6436881	18.1	C16H16	Partially hydrogenated PAH	PAHs	0
35.5	3-Buten-1-one, 1,4-diphenyl-	717640	2.0	C16H14O	Aromatic ketone	Oxygen-containing compounds	0.14395
35.6	Anthracene, 9,10-dihydro-	1282509	3.6	C14H12	Partially hydrogenated PAH	PAHs	0
35.8	1,2-Diphenylcyclopropane	469325.3	1.3	C15H14	Diaryl cycloalkane	Oligomers	0
39.4	Benzene, 1,1'-(1-butene-1,4-diyl)bis-, (Z)-	228133	0.6	C16H16	Diaryl alkene	Oligomers	0
		35576864					1.19483

Table S19. GC-MS analysis result of oil produced during catalytic pyrolysis of acid-leached Fe-impregnated sewage sludge char after two cycles of use with PS rate 1:4.

RT	Name	Area	ω, %	Formula	Category	Group	Oxygen content
4.5	Water	97205	0.2	H2O	Inorganic solvent	Other	0.001776
12.3	Benzene	1560602	3.5	C6H6	Aromatic hydrocarbon	Light aromatic fraction	0
15.2	Toluene	955976	2.1	C7H8	Alkylbenzene	Light aromatic fraction	0
17.4	Benzene, 1,3-dimethyl-	612356	1.4	C8H10	Alkylbenzene	Light aromatic fraction	0
18.2	Styrene	17382324	38.5	C8H8	Vinyl aromatic hydrocarbon	Light aromatic fraction	0
18.8	Benzene, (1-methylethyl)-	32994	0.1	C9H12	Alkylbenzene	Light aromatic fraction	0
19.3	Azetidine, 3-methyl-3-phenyl-	40754	0.1	C10H13N	Nitrogen-containing heterocycle	Oxygen-containing compounds	0
20.0	.alpha.-Methylstyrene	2132700	4.7	C9H10	Vinyl aromatic hydrocarbon	Light aromatic fraction	0
20.2	Phenacylidene diacetate	54648	0.1	C12H12O4	Aromatic ester	Oxygen-containing compounds	0.000291

20.2	Benzaldehyde	50044	0.1	C7H6O	Aromatic aldehyde	Oxygen-containing compounds	0.000151
20.3	Indane	32751	0.1	C9H10	Indane	Cyclic unsaturated compounds	0
20.4	Indane	11292	0.0	C9H10	Indane	Cyclic unsaturated compounds	0
20.9	Benzene, cyclopropyl-	181653	0.4	C9H10	Alkylbenzene	Light aromatic fraction	0
20.9	Benzene, 2-propenyl-	176998	0.4	C9H10	Alkenylbenzene	Light aromatic fraction	0
21.6	Benzene, 1-propynyl-	592143	1.3	C9H8	Alkynylbenzene	Light aromatic fraction	0
22.6	Naphthalene, 1,2-dihydro-	58262	0.1	C10H10	Partially hydrogenated PAH	Cyclic unsaturated compounds	0
22.8	1H-Indene, 1-(phenylmethylene)-	700136	1.6	C16H12	Diaryl-substituted indene	Oligomers	0
23.4	2-Methylindene	67337	0.1	C10H10	Alkyl-substituted indene	Cyclic unsaturated compounds	0
23.5	Benzene, 1-methyl-4-(1-propynyl)-	114486	0.3	C10H10	Alkynylbenzene	Light aromatic fraction	0
23.7	Cycloprop[a]indene, 1,1a,6,6a-tetrahydro-	116700	0.3	C10H10	Fused cyclic unsaturated hydrocarbon	Cyclic unsaturated compounds	0
24.3	Naphthalene	2229448	4.9	C10H8	PAH	PAHs	0
26.0	Naphthalene, 2-methyl-	109594	0.2	C11H10	Alkyl-substituted PAH	PAHs	0
26.4	1H-Indene, 1-ethylidene-	98803	0.2	C11H10	Alkenyl-substituted indene	Cyclic unsaturated compounds	0
27.3	Biphenyl	2310978	5.1	C12H10	Biphenyl hydrocarbon	Oligomers	0
28.5	Acenaphthene	75930	0.2	C12H10	Partially hydrogenated PAH	PAHs	0

29.2	1,1'-Biphenyl, 4-methyl-	193871	0.4	C13H12	Alkyl-substituted biphenyl	Oligomers	0
29.4	Acenaphthylene	146705	0.3	C12H8	PAH	PAHs	0
29.6	Ethylene, 1,1-diphenyl-	239716	0.5	C14H12	Diaryl alkene	Oligomers	0
29.9	(E)-Stilbene	120290	0.3	C14H12	Diaryl alkene	Oligomers	0
30.0	Bibenzyl	485814	1.1	C14H14	Diaryl alkane	Oligomers	0
30.6	Benzene, 1,1'-(1,2-dimethyl-1,2-ethanediyl)bis-, (R*,S*)-	285264	0.6	C16H18	Diaryl alkane	Oligomers	0
31.3	Naphthalene, 2-phenyl-	1146721	2.5	C16H12	PAH	PAHs	0
32.1	1,1'-Biphenyl, 4-ethenyl-	275804	0.6	C14H12	Alkenylbiphenyl	Oligomers	0
32.4	Fluorene	548397	1.2	C13H10	PAH	PAHs	0
32.9	Benzene, 1,1',1'',1'''-(1,2,3,4-cyclobutanetetrayl)tetrakis-	130808	0.3	C28H28	Polyaryl cycloalkane	Oligomers	0
33.2	Benzene, 1,1'-(1,3-propanediyl)bis-	2422220	5.4	C15H16	Diaryl alkane	Oligomers	0
34.0	Benzene, 1,1'-(1,2-ethanediyl)bis[4-methyl-	75267	0.2	C16H18	Alkyl-substituted diaryl alkane	Oligomers	0
35.3	Naphthalene, 1,2,3,4-tetrahydro-2-phenyl-	7046894	15.6	C16H16	Partially hydrogenated PAH	PAHs	0
35.6	Anthracene, 9,10-dihydro-	1863103	4.1	C14H12	Partially hydrogenated PAH	PAHs	0
37.8	1H-Indene, 2-phenyl-	136699	0.3	C15H12	Aryl-substituted indene	Oligomers	0
39.4	Benzene, 1,1'-(1-butene-1,4-diyl)bis-, (Z)-	234654	0.5	C16H16	Diaryl alkene	Oligomers	0
		45148343					0.002218

Table S20. GC-MS analysis result of oil produced during catalytic pyrolysis of acid-leached Fe-impregnated sewage sludge char after three cycles of use with PS rate 1:4.

**RT**	**Name**	**Area**	**ω, %**	**Formula**	**Category**	**Group**	**Oxygen content**
4.5	Water	151818	0.2	H2O	Inorganic solvent	Other	0.177619
12.3	Benzene	1848944	3.0	C6H6	Aromatic hydrocarbon	Light aromatic fraction	0
15.2	Toluene	1359501	2.2	C7H8	Alkylbenzene	Light aromatic fraction	0
17.4	Ethylbenzene	887851	1.5	C8H10	Alkylbenzene	Light aromatic fraction	0

18.2	Styrene	24559052	40.2	C8H8	Vinyl aromatic hydrocarbon	Light aromatic fraction	0
18.8	Benzene, (1-methylethyl)-	43134	0.1	C9H12	Alkylbenzene	Light aromatic fraction	0
19.3	Indane	73845	0.1	C9H10	Indane	Cyclic unsaturated compounds	0
19.3	Benzene, 1,1'-(1-ethenyl-1,3-propanediyl)bis-	81508	0.1	C17H18	Diaryl alkene	Oligomers	0
19.4	Benzene, propyl-	27499	0.0	C9H12	Alkylbenzene	Light aromatic fraction	0
20.0	.alpha.-Methylstyrene	2944735	4.8	C9H10	Vinyl aromatic hydrocarbon	Light aromatic fraction	0
20.2	Diglycolic acid, di(3-phenylpropyl) ester	31282	0.1	C22H26O5	Aromatic ester	Oxygen-containing compounds	0.021594
20.2	Azetidine, 3-methyl-3-phenyl-	33332	0.1	C10H13N	Nitrogen-containing heterocycle	Oxygen-containing compounds	0
20.2	Benzaldehyde	100738	0.2	C7H6O	Aromatic aldehyde	Oxygen-containing compounds	0.030152
20.3	Benzeneethanol, .beta.-ethenyl-	52356	0.1	C10H12O	Vinyl-substituted aromatic alcohol	Oxygen-containing compounds	0.010795
20.4	3-Phenyl-1-propanol, acetate	18130	0.0	C11H14O2	Aromatic ester	Oxygen-containing compounds	0
20.4	Fumaric acid, di(3-phenylpropyl) ester	17233	0.0	C20H22O4	Aromatic ester	Oxygen-containing compounds	0
20.9	Benzene, cyclopropyl-	239426	0.4	C9H10	Alkylbenzene	Light aromatic fraction	0
21.2	Pimelic acid, di(3-phenylpropyl) ester	35355	0.1	C20H24O4	Aromatic ester	Oxygen-containing compounds	0.019487
21.6	Benzene, 1-propynyl-	746010	1.2	C9H8	Alkynylbenzene	Light aromatic fraction	0
21.8	2,5-Dibenzyloxynitrobenzene	36094	0.1	C20H17NO4	Nitroaromatic ether	Oxygen-containing compounds	0.019083

22.6	Benzene, 1,3-diethenyl-	87369	0.1	C10H10	Divinylbenzene	Light aromatic fraction	0
22.8	Naphthalene, 1-phenyl-	261647	0.4	C16H12	Aryl-substituted PAH	PAHs	0
22.8	1H-Indene, 3-methyl-	104751	0.2	C10H10	Alkyl-substituted indene	Cyclic unsaturated compounds	0
23.4	2-Methylindene	94285	0.2	C10H10	Alkyl-substituted indene	Cyclic unsaturated compounds	0
23.5	Cycloprop[a]indene, 1,1a,6,6a-tetrahydro-	108177	0.2	C10H10	Fused cyclic unsaturated hydrocarbon	Cyclic unsaturated compounds	0
23.7	Naphthalene, 1,2-dihydro-	141895	0.2	C10H10	Partially hydrogenated PAH	Cyclic unsaturated compounds	0
24.3	Naphthalene	2787781	4.6	C10H8	PAH	PAHs	0
26.0	1H-Indene, 1-ethylidene-	147937	0.2	C11H10	Alkenyl-substituted indene	Cyclic unsaturated compounds	0
26.4	Naphthalene, 2-methyl-	130905	0.2	C11H10	PAH	PAHs	0
27.3	Biphenyl	2800341	4.6	C12H10	Biphenyl hydrocarbon	Oligomers	0
28.1	Diphenylmethane	209044	0.3	C13H12	Diaryl alkane	Oligomers	0
28.5	Naphthalene, 2-ethenyl-	105330	0.2	C12H10	PAH	PAHs	0
29.2	1,1'-Biphenyl, 4-methyl-	237672	0.4	C13H12	Alkyl-substituted biphenyl	Oligomers	0
29.4	Acenaphthylene	153633	0.3	C12H8	PAH	PAHs	0
29.6	9H-Fluorene, 9-methyl-	244459	0.4	C14H12	PAH	PAHs	0
29.6	(E)-Stilbene	284039	0.5	C14H12	Diaryl alkene	Oligomers	0
29.9	cis-Stilbene	160553	0.3	C14H12	Diaryl alkene	Oligomers	0
30.0	Bibenzyl	652345	1.1	C14H14	Diaryl alkane	Oligomers	0
30.6	Benzene, 1,1'-(1-methyl-1,2-ethanediyl)bis-	420540	0.7	C15H16	Diaryl alkane	Oligomers	0
31.3	1H-Indene, 1-(phenylmethylene)-	453830	0.7	C16H12	Diaryl-substituted indene	Oligomers	0
32.1	1,1'-Biphenyl, 4-ethenyl-	332086	0.5	C14H12	Alkenylbiphenyl	Oligomers	0
32.4	Fluorene	674645	1.1	C13H10	PAH	PAHs	0
32.9	Benzene, 1,1',1'',1'''-(1,2,3,4-cyclobutanetetrayl)tetrakis-	190201	0.3	C28H28	Polyaryl cycloalkane	Oligomers	0

33.2	Benzene, 1,1'-(1,3-propanediyl)bis-	3114849	5.1	C15H16	Diaryl alkane	Oligomers	0
33.6	benzene, 1,1'-(1-methylene-1,2-ethanediyl)bis-	76761	0.1	C15H16	Diaryl alkene	Oxygen-containing compounds	0
34.0	Benzene, 1,1'-(1-methyl-1,3-propanediyl)bis-	131006	0.2	C16H18	Diaryl alkane	Light aromatic fraction	0
35.3	Naphthalene, 1,2,3,4-tetrahydro-2-phenyl-	9560559	15.7	C16H16	Partially hydrogenated PAH	Light aromatic fraction	0
35.5	3-Buten-1-one, 1,4-diphenyl-	755997	1.2	C16H14O	Aromatic ketone	Light aromatic fraction	0.086369
35.6	Anthracene, 9,10-dihydro-	2287393	3.7	C14H12	Partially hydrogenated PAH	Light aromatic fraction	0
35.8	1,2-Diphenylcyclopropane	596013	1.0	C15H14	Diaryl cycloalkane	Light aromatic fraction	0
37.8	1H-Indene, 2-phenyl-	165667	0.3	C15H12	Aryl-substituted indene	Cyclic unsaturated compounds	0
39.3	Benzene, 1,1'-(1-butene-1,4-diyl)bis-, (Z)-	322433	0.5	C16H16	Diaryl alkene	Oligomers	0
		61081984					0.365099

Table S21. GC-MS analysis result of oil produced during catalytic pyrolysis of acid-leached Fe-impregnated sewage sludge char after regenerated with PS rate 1:4.

RT	Name	Area	ω, %	Formula	Category	Group	Oxygen content
4.46	Water	134696.9	0.54	H2O	Inorganic solvent	Other	0.47957
12.28	Benzene	2046742.7	8.18	C6H6	Aromatic hydrocarbon	Light aromatic fraction	0
15.17	Toluene	642172	2.57	C7H8	Alkylbenzene	Light aromatic fraction	0
17.42	Ethylbenzene	231936.5	0.93	C8H10	Alkylbenzene	Light aromatic fraction	0
18.20	Styrene	6994694.2	27.95	C8H8	Vinyl aromatic hydrocarbon	Light aromatic fraction	0
19.24	Benzaldehyde, 4-(1-phenyl-2-propenyloxy)-	9176.4	0.04	C16H14O2	Aromatic aldehyde	Oxygen-containing compounds	0.005371
19.96	.alpha.-Methylstyrene	915654.4	3.66	C9H10	Vinyl aromatic hydrocarbon	Light aromatic fraction	0

20.16	Diglycolic acid, di(3-phenylpropyl) ester	6586.4	0.03	C22H26O5	Aromatic ester	Oxygen-containing compounds	0.006478
20.91	Benzeneethanol, .beta.-ethenyl-	61497.2	0.25	C10H12O	Vinyl-substituted aromatic alcohol	Oxygen-containing compounds	0.026988
21.55	Indene	200188.4	0.80	C9H8	Indene	Cyclic unsaturated compounds	0
22.71	1H-Indene, 1-(phenylmethylene)-	356158.2	1.42	C16H12	Diaryl-substituted indene	Oligomers	0
23.37	2-Methylindene	28889.7	0.12	C10H10	Alkyl-substituted indene	Cyclic unsaturated compounds	0
23.52	Benzene, 1-methyl-4-(1-propynyl)-	87852.9	0.35	C10H10	Alkynylbenzene	Light aromatic fraction	0
23.72	Naphthalene, 1,2-dihydro-	29338.4	0.12	C10H10	Partially hydrogenated PAH	Cyclic unsaturated compounds	0
24.21	1H-Indene, 2-phenyl-	311797.1	1.25	C15H12	Aryl-substituted indene	Cyclic unsaturated compounds	0
24.27	Naphthalene	1050407.2	4.20	C10H8	PAH	PAHs	0
26.03	Naphthalene, 2-methyl-	43954.1	0.18	C11H10	PAH	PAHs	0
26.36	Naphthalene, 1-methyl-	44970	0.18	C11H10	PAH	PAHs	0
27.26	Biphenyl	1091191.3	4.36	C12H10	Biphenyl hydrocarbon	Oligomers	0
28.09	Diphenylmethane	74910.1	0.30	C13H12	Diaryl alkane	Oligomers	0
28.49	Acenaphthene	35765.6	0.14	C12H10	Partially hydrogenated PAH	PAHs	0
29.17	1,1-Diphenyl-2-propanol	82521.2	0.33	C15H16O	Diaryl alcohol	Oxygen-containing compounds	0.02487
29.35	Acenaphthylene	57696.6	0.23	C12H8	PAH	PAHs	0
29.61	(E)-Stilbene	121969.9	0.49	C14H12	Diaryl alkene	Oligomers	0
29.91	Anthracene, 9,10-dihydro-9-(1-methylpropyl)-	58582.1	0.23	C18H20	Partially hydrogenated PAH	PAHs	0
30.03	Bibenzyl	196947	0.79	C14H14	Diaryl alkane	Oligomers	0

30.63	Benzene, 1,1'-(1,2-dimethyl-1,2-ethanediyl)bis-, (R*,S*)-	141934.3	0.57	C16H18	Diaryl alkane	Oligomers	0
31.27	Acephenanthrylene, 4,5-dihydro-	848557.8	3.39	C16H12	Partially hydrogenated PAH	PAHs	0
32.12	1,1'-Biphenyl, 4-ethenyl-	130554	0.52	C14H12	Alkenylbiphenyl	Oligomers	0
32.39	Fluorene	374719.3	1.50	C13H10	PAH	PAHs	0
32.87	Phenanthrene, 9,10-dihydro-	81182.1	0.32	C14H12	Partially hydrogenated PAH	PAHs	0
33.18	Benzene, 1,1'-(1,3-propanediyl)bis-	1829283.4	7.31	C15H16	Diaryl alkane	Oligomers	0
33.97	Benzene, 1,1'-(1-methyl-1,3-propanediyl)bis-	79978.9	0.32	C16H18	Diaryl alkane	Light aromatic fraction	0
35.26	Naphthalene, 1,2,3,4-tetrahydro-2-phenyl-	4129191.4	16.50	C16H16	Partially hydrogenated PAH	Light aromatic fraction	0
35.60	Anthracene, 9,10-dihydro-	1264106.8	5.05	C14H12	Partially hydrogenated PAH	Light aromatic fraction	0
35.77	1,2-Diphenylcyclopropane	351843.9	1.41	C15H14	Diaryl cycloalkane	Light aromatic fraction	0
37.76	Anthracene, 9-methyl-	71117.7	0.28	C15H12	Alkyl-substituted PAH	PAHs	0
38.03	Benzene, 1,1'-(3-methyl-1-propene-1,3-diyl)bis-	688423.6	2.75	C16H16	Diaryl alkene	Oligomers	0
39.33	Benzene, 1,1'-(1-butene-1,4-diyl)bis-, (Z)-	115521.4	0.46	C16H16	Diaryl alkene	Oligomers	0
		25022711.1					0.543278

Table S22. One-way ANOVA and Tukey's HSD analysis of PyGas yield and composition presented in Figures 7 and 8. Values are mean ± SD from three independent experiments (n = 3). Different lowercase letters within the same row indicate statistically significant differences according to Tukey's HSD test (p < 0.05); values sharing at least one letter are not significantly different.

Component	ANOVA $\boldsymbol{p}$	PS	1:1	1:2	1:4	1:10
		Figure 7a — SSA/PS: PyGas component yields, wt.% of PS feed				
H_2	1.11×10^{-8}	1.040 ± 0.160 b	1.360 ± 0.100 a	0.230 ± 0.030 c	0.090 ± 0.030 c	0.090 ± 0.040 c
CO	3.26×10^{-7}	2.720 ± 0.210 b	5.290 ± 0.790 a	1.320 ± 0.120 c	0.940 ± 0.250 c	0.790 ± 0.190 c
CH_4	1.88×10^{-7}	4.630 ± 0.630 a	4.670 ± 0.490 a	2.630 ± 0.190 b	0.800 ± 0.210 c	0.800 ± 0.110 c
CO_2	3.21×10^{-10}	1.370 ± 0.210 bc	7.760 ± 0.590 a	1.580 ± 0.270 b	0.720 ± 0.110 c	0.740 ± 0.070 c

C_2H_4	1.33×10^{-7}	2.500 ± 0.310 a	2.740 ± 0.370 a	0.800 ± 0.090 b	0.310 ± 0.080 b	0.430 ± 0.060 b
C_2H_6	2.56×10^{-5}	0.350 ± 0.040 a	0.460 ± 0.090 a	0.320 ± 0.080 a	0.060 ± 0.020 b	0.080 ± 0.030 b
Total PyGas	3.56×10^{-9}	12.600 ± 1.120 b	15.600 ± 1.180 a	6.870 ± 0.370 c	2.920 ± 0.230 d	2.930 ± 0.170 d

Figure 7b — SSC/PS: PyGas component yields, wt.% of PS feed

Component	ANOVA p	PS	1:1	1:2	1:4	1:10
H_2	3.33×10^{-9}	1.040 ± 0.160 **b**	2.570 ± 0.220 **a**	0.270 ± 0.050 **c**	0.140 ± 0.020 **c**	0.710 ± 0.070 **b**
CO	1.74×10^{-8}	2.720 ± 0.210 **b**	9.180 ± 0.830 **a**	1.940 ± 0.610 **b**	0.490 ± 0.110 **c**	2.610 ± 0.490 **b**
CH_4	2.01×10^{-7}	4.630 ± 0.630 **b**	7.050 ± 0.610 **a**	2.050 ± 0.640 **c**	0.970 ± 0.100 **c**	2.000 ± 0.180 **c**
CO_2	4.21×10^{-10}	1.370 ± 0.210 **bc**	11.020 ± 0.920 **a**	1.990 ± 0.380 **b**	0.530 ± 0.110 **c**	1.690 ± 0.130 **bc**
C_2H_4	4.52×10^{-6}	2.500 ± 0.310 **b**	3.710 ± 0.340 **a**	1.120 ± 0.210 **c**	0.780 ± 0.350 **c**	2.080 ± 0.380 **b**
C_2H_6	1.52×10^{-3}	0.350 ± 0.040 **ab**	0.480 ± 0.030 **a**	0.170 ± 0.040 **bc**	0.100 ± 0.170 **c**	0.240 ± 0.020 **bc**
Total PyGas	1.12×10^{-6}	12.600 ± 1.120 **b**	15.800 ± 1.960 **a**	7.540 ± 0.880 **c**	3.020 ± 0.510 **d**	9.310 ± 0.810 **c**

Figure 7c — Fe-LSSC/PS: PyGas component yields, wt.% of PS feed

Component	ANOVA p	PS	1:1	1:2	1:4	1:10
H_2	1.58×10^{-5}	1.040 ± 0.160 **b**	2.400 ± 0.490 **a**	0.790 ± 0.070 **b**	0.750 ± 0.090 **b**	0.520 ± 0.070 **b**
CO	1.49×10^{-7}	2.720 ± 0.210 **c**	4.600 ± 0.500 **b**	1.190 ± 0.100 **cd**	0.900 ± 0.240 **d**	8.560 ± 1.220 **a**
CH_4	1.46×10^{-9}	4.630 ± 0.630 **b**	7.790 ± 0.540 **a**	0.700 ± 0.070 **d**	1.820 ± 0.130 **c**	0.370 ± 0.040 **d**
CO_2	5.19×10^{-8}	1.370 ± 0.210 **bc**	8.040 ± 1.120 **a**	0.640 ± 0.100 **c**	0.660 ± 0.120 **c**	2.390 ± 0.370 **b**
C_2H_2	5.24×10^{-8}	0.000 ± 0.000 **b**	0.000 ± 0.000 **b**	0.000 ± 0.000 **b**	0.000 ± 0.000 **b**	0.230 ± 0.040 **a**
C_2H_4	2.35×10^{-7}	2.500 ± 0.310 **b**	5.150 ± 0.830 **a**	0.640 ± 0.080 **cd**	1.460 ± 0.220 **bc**	0.000 ± 0.000 **d**
C_2H_6	3.86×10^{-10}	0.350 ± 0.040 **b**	0.000 ± 0.000 **c**	0.120 ± 0.030 **c**	0.150 ± 0.040 **c**	1.330 ± 0.110 **a**
C_3H_6	5.29×10^{-8}	0.000 ± 0.000 **b**	0.000 ± 0.000 **b**	0.000 ± 0.000 **b**	0.000 ± 0.000 **b**	0.230 ± 0.040 **a**
Total PyGas	4.12×10^{-8}	12.600 ± 1.120 **b**	27.980 ± 2.810 **a**	4.100 ± 0.670 **c**	5.730 ± 1.160 **c**	13.630 ± 1.370 **b**

Figure 7d — Fe-LSSC reuse/regeneration

Component	ANOVA p	PS	Reuse 1	Reuse 2	Reuse 3	Regenerated
H_2	6.48×10^{-7}	1.040 ± 0.160 **b**	0.720 ± 0.130 **b**	0.140 ± 0.030 **c**	0.160 ± 0.020 **c**	1.680 ± 0.250 **a**
CO	9.73×10^{-8}	2.720 ± 0.210 **a**	1.360 ± 0.160 **c**	0.480 ± 0.060 **d**	0.750 ± 0.130 **d**	2.170 ± 0.250 **b**
CH_4	3.58×10^{-6}	4.630 ± 0.630 **a**	2.130 ± 0.340 **b**	1.010 ± 0.140 **c**	1.850 ± 0.240 **bc**	2.580 ± 0.280 **b**
CO_2	4.57×10^{-6}	1.370 ± 0.210 **a**	0.860 ± 0.120 **b**	0.280 ± 0.040 **c**	0.500 ± 0.080 **c**	1.130 ± 0.100 **ab**
C_2H_4	5.24×10^{-6}	2.500 ± 0.310 **ab**	1.970 ± 0.200 **b**	0.620 ± 0.140 **c**	0.970 ± 0.170 **c**	2.900 ± 0.440 **a**
C_2H_6	1.71×10^{-5}	0.350 ± 0.040 **a**	0.270 ± 0.020 **a**	0.090 ± 0.030 **b**	0.090 ± 0.040 **b**	0.320 ± 0.060 **a**
Total PyGas	2.23×10^{-7}	12.600 ± 1.120 **a**	7.310 ± 0.600 **b**	2.630 ± 0.400 **c**	4.320 ± 0.460 **c**	10.780 ± 1.270 **a**

Figure 8a — SSA/PS: PyGas composition, vol.%

Component	ANOVA p	PS	1:1	1:2	1:4	1:10
H_2	2.75×10^{-7}	49.660 ± 2.920 **a**	46.580 ± 2.360 **a**	28.450 ± 1.580 **b**	27.340 ± 2.640 **b**	28.070 ± 1.640 **b**
CO	4.41×10^{-6}	9.300 ± 0.760 **d**	12.990 ± 1.800 **c**	11.710 ± 0.980 **cd**	21.440 ± 0.710 **a**	17.760 ± 1.970 **b**
CH_4	1.17×10^{-6}	27.890 ± 0.640 **b**	20.200 ± 1.280 **c**	40.920 ± 2.310 **a**	32.090 ± 2.110 **b**	31.620 ± 2.100 **b**
CO_2	6.35×10^{-6}	2.960 ± 0.230 **c**	12.050 ± 0.720 **a**	8.820 ± 0.980 **b**	10.400 ± 1.510 **ab**	10.590 ± 1.180 **ab**
C_2H_4	0.0264	9.070 ± 1.620 **ab**	7.130 ± 0.380 **b**	7.490 ± 1.540 **ab**	7.390 ± 0.820 **ab**	10.270 ± 0.670 **a**
C_2H_6	1.81×10^{-4}	1.110 ± 0.070 **b**	1.040 ± 0.130 **b**	2.600 ± 0.360 **a**	1.330 ± 0.230 **b**	1.690 ± 0.390 **b**
H_2 yield, g/g PS	1.11×10^{-8}	0.010 ± 0.002 **b**	0.014 ± 0.001 **a**	0.002 ± 0.000 **c**	0.001 ± 0.000 **c**	0.001 ± 0.000 **c**

Figure 8b — SSC/PS: PyGas composition, vol.%						
Component	ANOVA p	PS	1:1	1:2	1:4	1:10
H_2	4.05×10^{-6}	49.660 ± 2.920 **a**	52.010 ± 3.710 **a**	31.590 ± 1.810 **b**	35.800 ± 1.790 **b**	50.510 ± 2.240 **a**
CO	6.00×10^{-4}	9.300 ± 0.760 **b**	13.380 ± 2.850 **a**	16.320 ± 0.590 **a**	9.130 ± 0.590 **b**	13.420 ± 1.270 **a**
CH_4	2.04×10^{-5}	27.890 ± 0.640 **a**	18.080 ± 3.630 **b**	30.240 ± 0.680 **a**	31.720 ± 0.520 **a**	18.120 ± 3.030 **b**
CO_2	1.85×10^{-6}	2.960 ± 0.230 **c**	10.150 ± 0.910 **a**	10.590 ± 0.930 **a**	6.210 ± 0.560 **b**	5.490 ± 1.130 **b**
C_2H_4	6.61×10^{-5}	9.070 ± 1.620 **bc**	5.730 ± 1.260 **c**	9.940 ± 1.410 **b**	15.390 ± 1.210 **a**	11.340 ± 0.970 **b**
C_2H_6	3.98×10^{-6}	1.110 ± 0.070 **b**	0.650 ± 0.110 **c**	1.320 ± 0.140 **b**	1.750 ± 0.090 **a**	1.130 ± 0.120 **b**
H_2 yield, g/g PS	3.33×10^{-9}	0.010 ± 0.002 **b**	0.026 ± 0.002 **a**	0.003 ± 0.001 **c**	0.001 ± 0.000 **c**	0.007 ± 0.001 **b**
Figure 8c — Fe-LSSC/PS: PyGas composition, vol.%						
Compo-nent	ANOVA p	PS	1:1	1:2	1:4	1:10
H_2	9.60×10^{-7}	49.660 ± 2.920 **c**	53.610 ± 3.260 **c**	75.280 ± 3.430 **a**	62.810 ± 4.640 **b**	36.820 ± 2.240 **d**
CO	1.50×10^{-11}	9.300 ± 0.760 **b**	7.390 ± 0.140 **bc**	8.120 ± 1.460 **bc**	5.390 ± 1.200 **c**	43.680 ± 1.850 **a**
CH_4	5.44×10^{-8}	27.890 ± 0.640 **a**	22.050 ± 1.710 **b**	8.430 ± 0.690 **c**	19.230 ± 3.390 **b**	3.370 ± 0.330 **d**
CO_2	3.13×10^{-8}	2.960 ± 0.230 **b**	8.170 ± 0.930 **a**	2.760 ± 0.220 **b**	2.490 ± 0.260 **b**	7.720 ± 0.280 **a**
C_2H_2	2.19×10^{-7}	0.000 ± 0.000 **b**	0.000 ± 0.000 **b**	0.000 ± 0.000 **b**	0.000 ± 0.000 **b**	1.340 ± 0.270 **a**
C_2H_4	1.74×10^{-5}	9.070 ± 1.620 **a**	8.770 ± 2.120 **a**	4.640 ± 0.700 **b**	9.270 ± 0.880 **a**	0.000 ± 0.000 **c**
C_2H_6	1.79×10^{-8}	1.110 ± 0.070 **b**	0.000 ± 0.000 **c**	0.780 ± 0.260 **bc**	0.820 ± 0.210 **bc**	6.310 ± 0.820 **a**
C_3H_6	1.82×10^{-8}	0.000 ± 0.000 **b**	0.000 ± 0.000 **b**	0.000 ± 0.000 **b**	0.000 ± 0.000 **b**	0.770 ± 0.120 **a**
H_2 yield, g/g PS	1.58×10^{-5}	0.010 ± 0.002 **b**	0.024 ± 0.005 **a**	0.008 ± 0.001 **b**	0.007 ± 0.001 **b**	0.005 ± 0.001 **b**
Figure 8d — Fe-LSSC reuse/regeneration: PyGas composition, vol.%						
Compo-nent	ANOVA p	PS	Reuse 1	Reuse 2	Reuse 3	Regenerated
H_2	7.46×10^{-7}	49.660 ± 2.920 **b**	55.770 ± 2.840 **b**	38.290 ± 4.190 **c**	29.360 ± 3.460 **c**	68.330 ± 3.750 **a**
CO	1.37×10^{-3}	9.300 ± 0.760 **ab**	7.520 ± 1.150 **bc**	9.240 ± 0.200 **ab**	9.740 ± 0.470 **a**	6.370 ± 0.890 **c**
CH_4	1.84×10^{-6}	27.890 ± 0.640 **bc**	20.740 ± 1.400 **cd**	34.620 ± 3.180 **ab**	42.300 ± 4.610 **a**	13.320 ± 2.690 **d**
CO_2	0.0140	2.960 ± 0.230 **ab**	3.010 ± 0.280 **ab**	3.480 ± 0.650 **ab**	4.110 ± 0.910 **a**	2.100 ± 0.370 **b**
C_2H_4	4.72×10^{-4}	9.070 ± 1.620 **b**	11.560 ± 0.670 **ab**	12.720 ± 0.870 **a**	13.430 ± 0.670 **a**	9.010 ± 0.580 **b**
C_2H_6	0.0243	1.110 ± 0.070 **ab**	1.400 ± 0.390 **ab**	1.650 ± 0.220 **a**	1.060 ± 0.310 **ab**	0.870 ± 0.110 **b**
H_2 yield, g/g PS	6.48×10^{-7}	0.010 ± 0.002 **b**	0.007 ± 0.001 **b**	0.001 ± 0.000 **c**	0.002 ± 0.000 **c**	0.017 ± 0.003 **a**

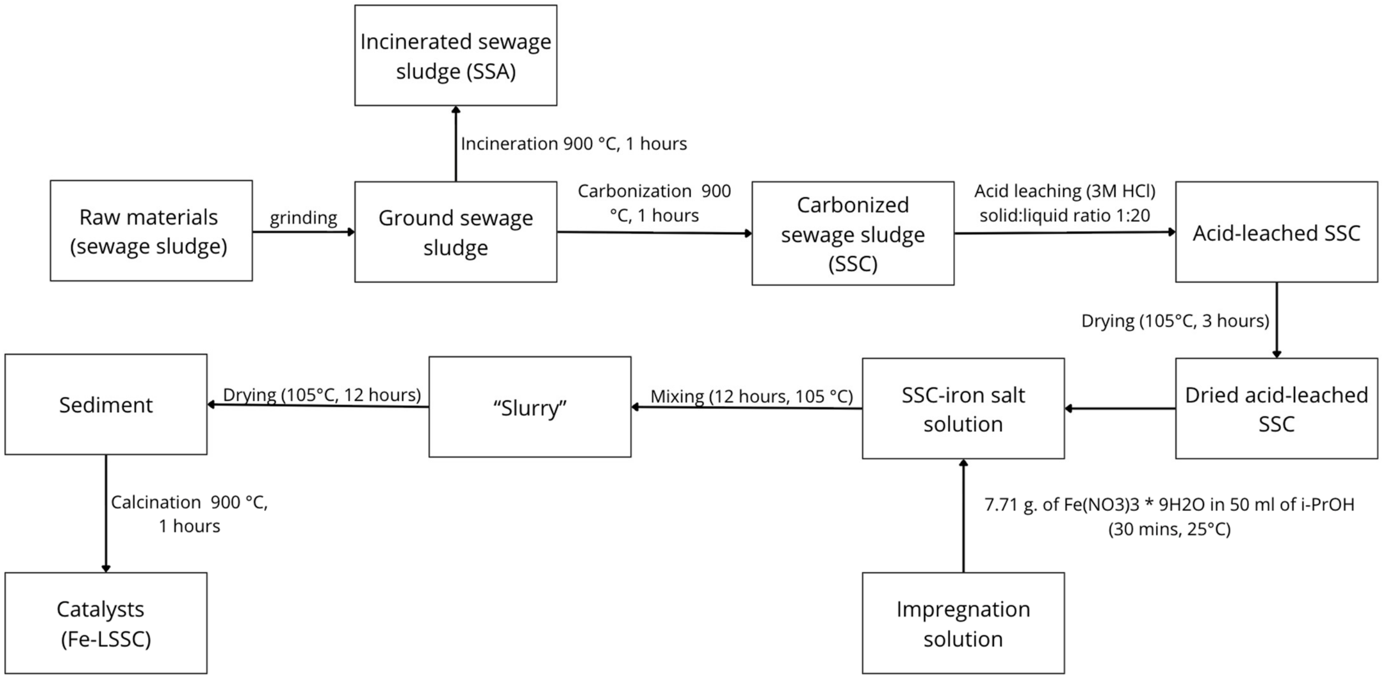

Figure S1. Preparation process of a sewage sludge-derived catalyst.

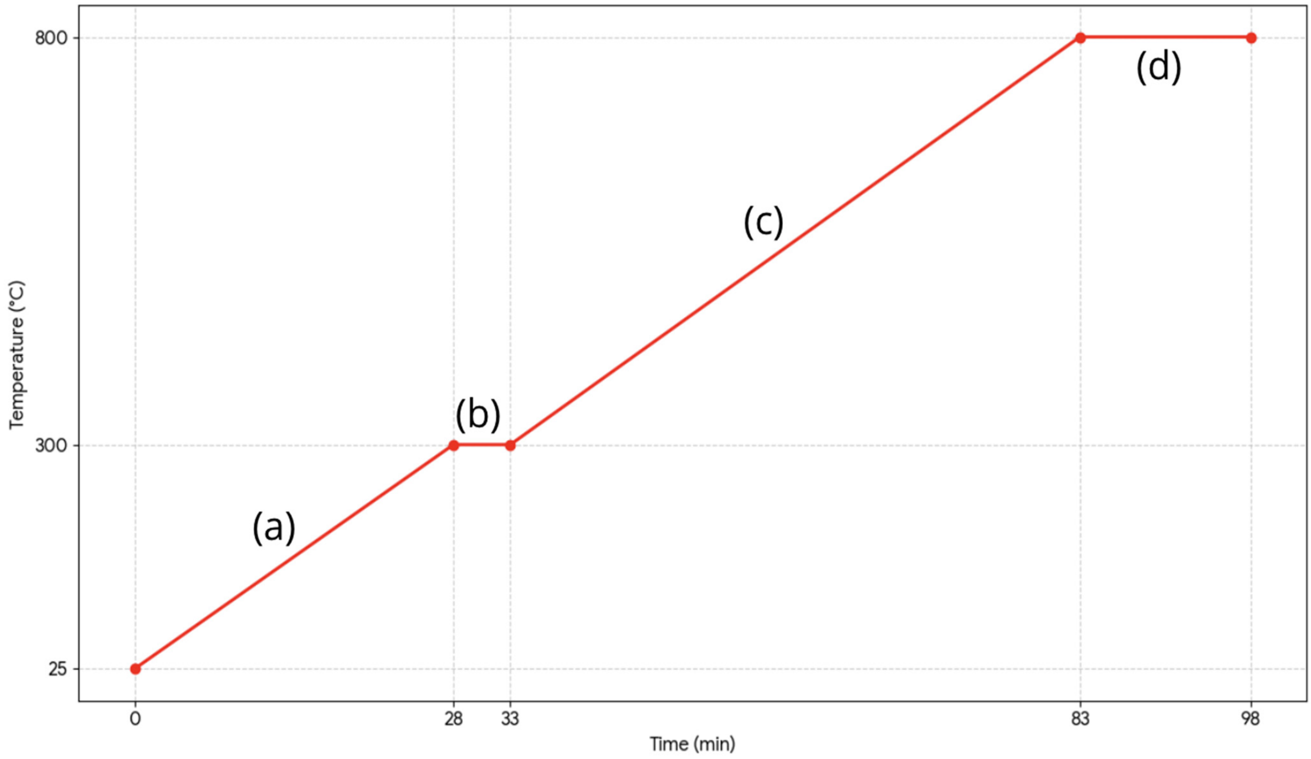

Figure S2. Heating program used for the slow pyrolysis process.

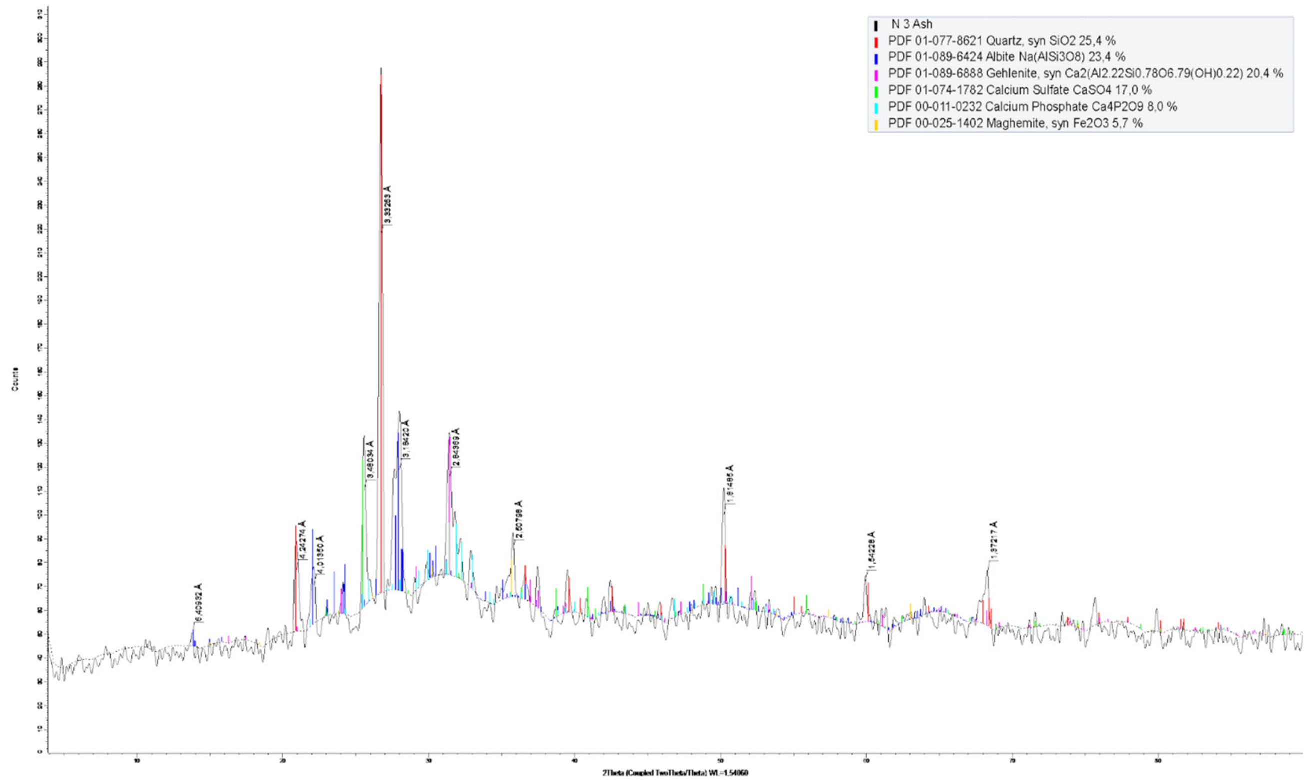
N 3 Ash
PDF 01-077-8621 Quartz, syn SiO2 25,4 %
PDF 01-089-6424 Albite Na(AlSi3O8) 23,4 %
PDF 01-089-6888 Gehlenite, syn Ca2(Al2.22Si0.78O6.79(OH)0.22) 20,4 %
PDF 01-074-1782 Calcium Sulfate CaSO4 17,0 %
PDF 00-011-0232 Calcium Phosphate Ca4P2O9 8,0 %
PDF 00-025-1402 Maghemite, syn Fe2O3 5,7 %

(**a**)

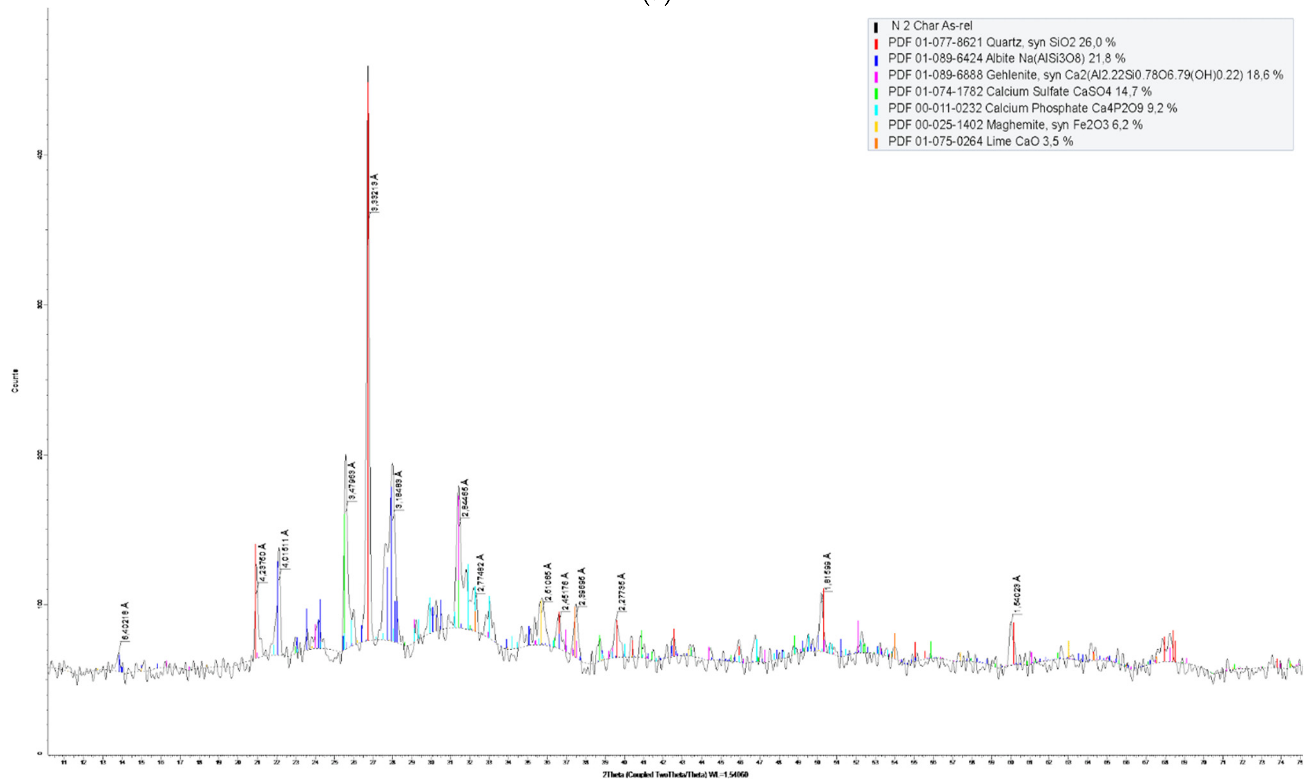
N 2 Char As-rel
PDF 01-077-8621 Quartz, syn SiO2 26,0 %
PDF 01-089-6424 Albite Na(AlSi3O8) 21,8 %
PDF 01-089-6888 Gehlenite, syn Ca2(Al2.22Si0.78O6.79(OH)0.22) 18,6 %
PDF 01-074-1782 Calcium Sulfate CaSO4 14,7 %
PDF 00-011-0232 Calcium Phosphate Ca4P2O9 9,2 %
PDF 00-025-1402 Maghemite, syn Fe2O3 6,2 %
PDF 01-075-0264 Lime CaO 3,5 %

(**b**)

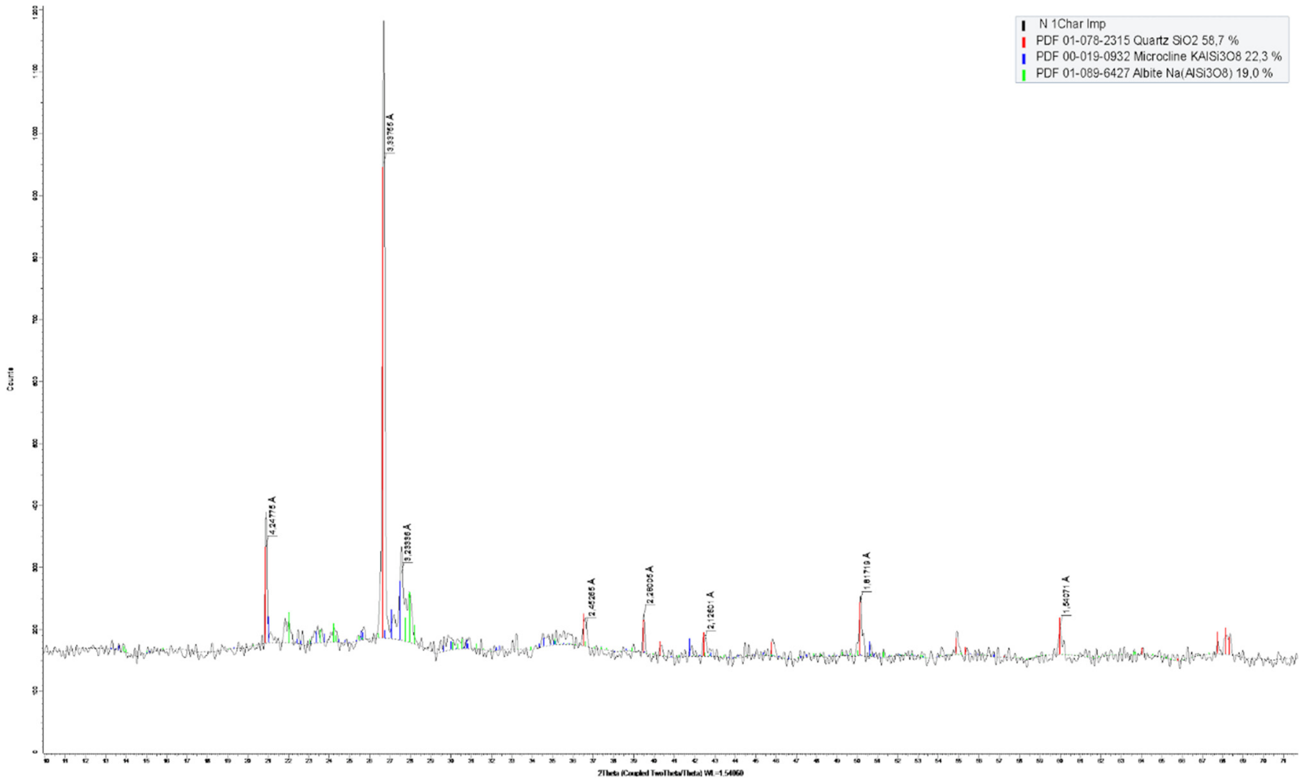

(**c**)

Figure S3. XRD analysis of sewage sludge-derived catalysts: (**a**) Sewage sludge ash, wt%. (**b**) Sewage sludge char, wt%. (**c**) Acid-leached Fe-impregnated sewage sludge char, wt%.

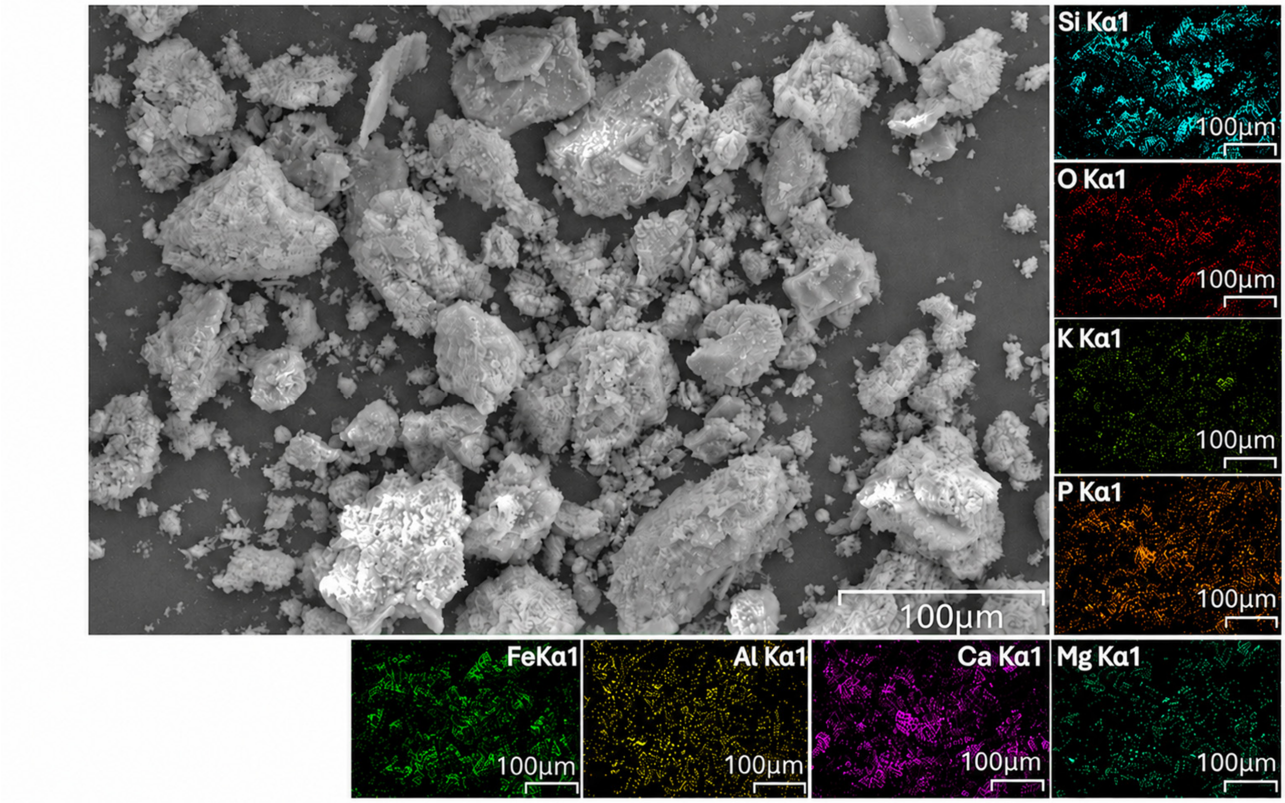

(**a**)

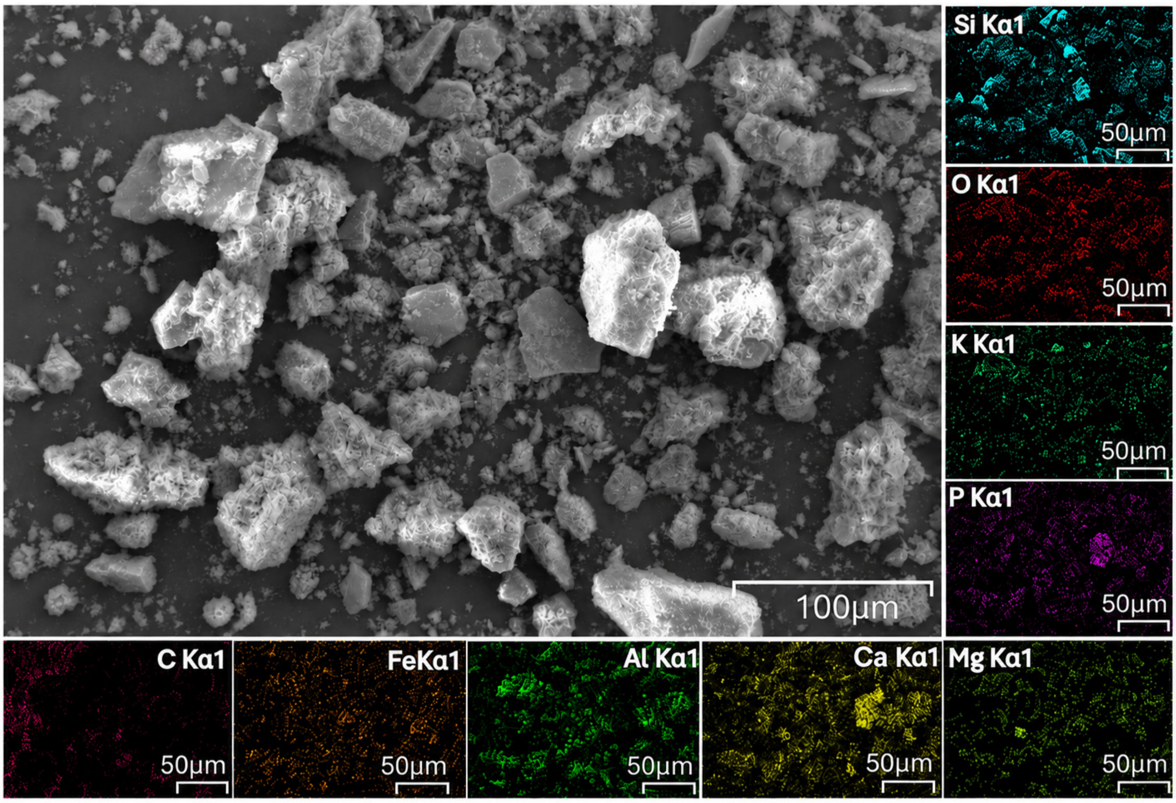

(**b**)

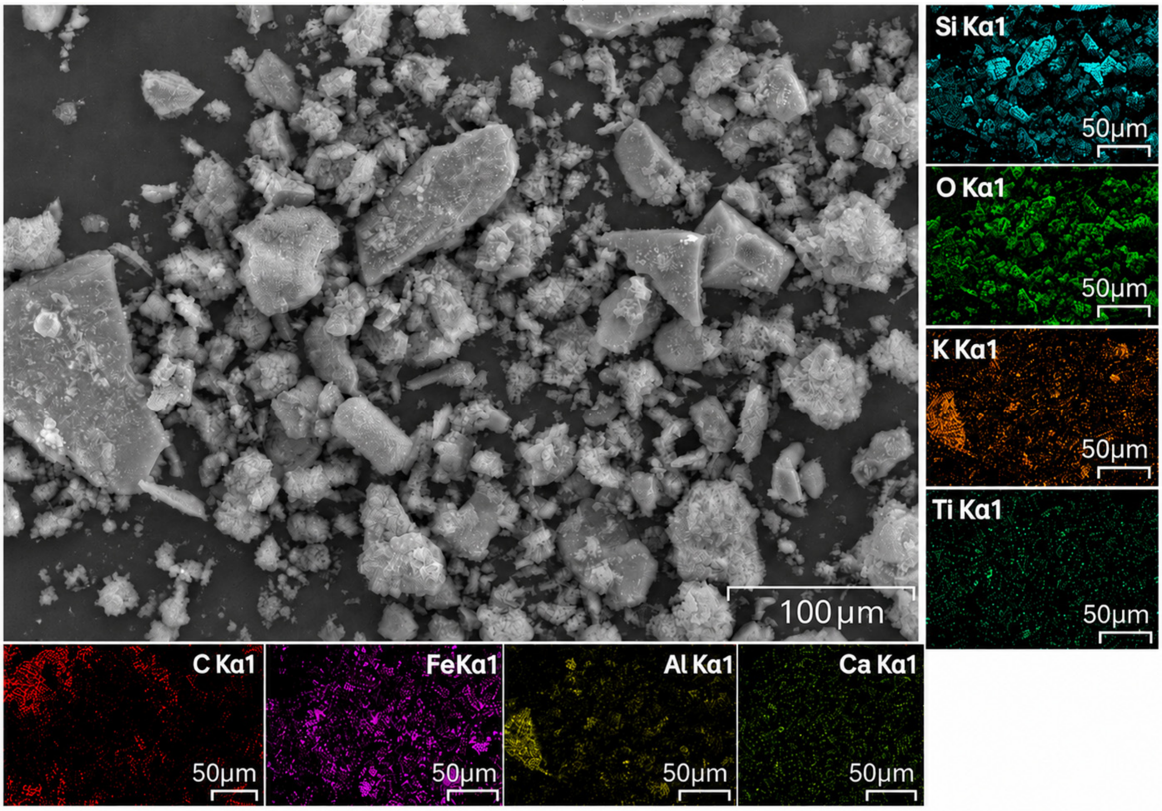

(**c**)

Figure S4. SEM-EDX analysis of sewage sludge-derived catalysts: (**a**) Sewage sludge ash. (**b**) Sewage sludge char. (**c**) Acid-leached Fe-impregnated sewage sludge char.
